# Synthesis and Biological Evaluation of 3-Alkyl-1,5-Diaryl-1*H*-Pyrazoles as Rigid Analogues of Combretastatin A-4 with Potent Antiproliferative Activity

**DOI:** 10.1371/journal.pone.0128710

**Published:** 2015-06-10

**Authors:** Qile Xu, Huan Qi, Maolin Sun, Daiying Zuo, Xuewei Jiang, Zhiyong Wen, Zhiwei Wang, Yingliang Wu, Weige Zhang

**Affiliations:** 1 Department Key Laboratory of Structure-Based Drug Design and Discovery Ministry of Education, Shenyang Pharmaceutical University, Shenyang, China; 2 Department of Pharmacology, Shenyang Pharmaceutical University, Shenyang, China; Aligarh Muslim University, INDIA

## Abstract

A series of novel 3-alkyl-1,5-diaryl-1*H*-pyrazoles were synthesized as combretastatin A-4 (CA-4) analogues and evaluated for antiproliferative activity against three human cancer cell lines (SGC-7901, A549 and HT-1080). Most of the target compounds displayed moderate to potent antiproliferative activity, and **7k** was found to be the most potent compound. Structure-activity relationships indicated that compounds with a trimethoxyphenyl A-ring at the N-1 position of the pyrazole skeleton were more potent than those with the A-ring at the C-5 position. Tubulin polymerization and immunostaining experiments revealed that **7k** potently inhibited tubulin polymerization and disrupted tubulin microtubule dynamics in a manner similar to CA-4. Computational modelling demonstrated that the binding of **7k** to the colchicine binding site on microtubules may involve a similar mode as CA-4.

## Introduction

Microtubules are an essential component of the cytoskeleton and have many cellular functions, including regulation of motility, cell division, organelle transport, maintenance of cell morphology and signal transduction [1]. Their importance in mitosis and cell division make microtubules an obvious and important target for the design and development of antitumour drugs [2]. Microtubule-targeting agents can be grouped into two classes: those that inhibit the polymerization process (microtubule destabilising agents) and those that promote the polymerization of tubulin (microtubule stabilising agents) [3]. Both classes disrupt microtubule/tubulin dynamics by binding to the protein tubulin, an α,β-heterodimer that forms the core of the microtubule and arrests cells during mitosis, leading to cell death. There are at least four characterised binding sites on tubulin: the taxane site, the laulimalide site, the vinca site and the colchicine site [4]. Many compounds that bind tubulin, such as paclitaxel, vinblastine and eribulin, are in clinical use for chemotherapy, and these drugs act by binding to the taxane site or the vinca site. While there are no clinically approved anticancer agents that bind to the colchicine site on tubulin, a number of effective inhibitors of tubulin are currently being investigated in clinical studies [5].

Combretastatin A-4 (CA-4, **1**) is one of the most effective inhibitors of tubulin found to bind to the colchicine site and whose impressive vascular disrupting activity leads to its clinical development [6]. CA-4 (**1**) was first isolated by Pettit et al. from the bark of the South African willow tree *Combretum caffrum* in 1989 [7], and it exhibits potent antiproliferative activity against a broad spectrum of human cancer lines, including those that show multidrug resistance [8]. Unfortunately, the *cis* double bond of **1** readily isomerizes to the more stable *trans* isomer under external stimuli, such as light or heat, resulting in complete loss of cytotoxicity [9]. Therefore, to retain the appropriate configuration of the two adjacent aryl groups required for bioactivity, researchers have synthesized a wide variety of *cis*-restricted analogues of **1** [4,10,11]. Structure-activity relationship (SAR) studies indicated that the common key structural feature of these analogues was two *cis*-orientated aromatic rings, the A-ring and B-ring, with the A-ring preferred as a 3,4,5-trimethoxy-substituted phenyl group and the B-ring preferred as a *p*-methoxy-substituted phenyl group [12].

**Fig 1:**
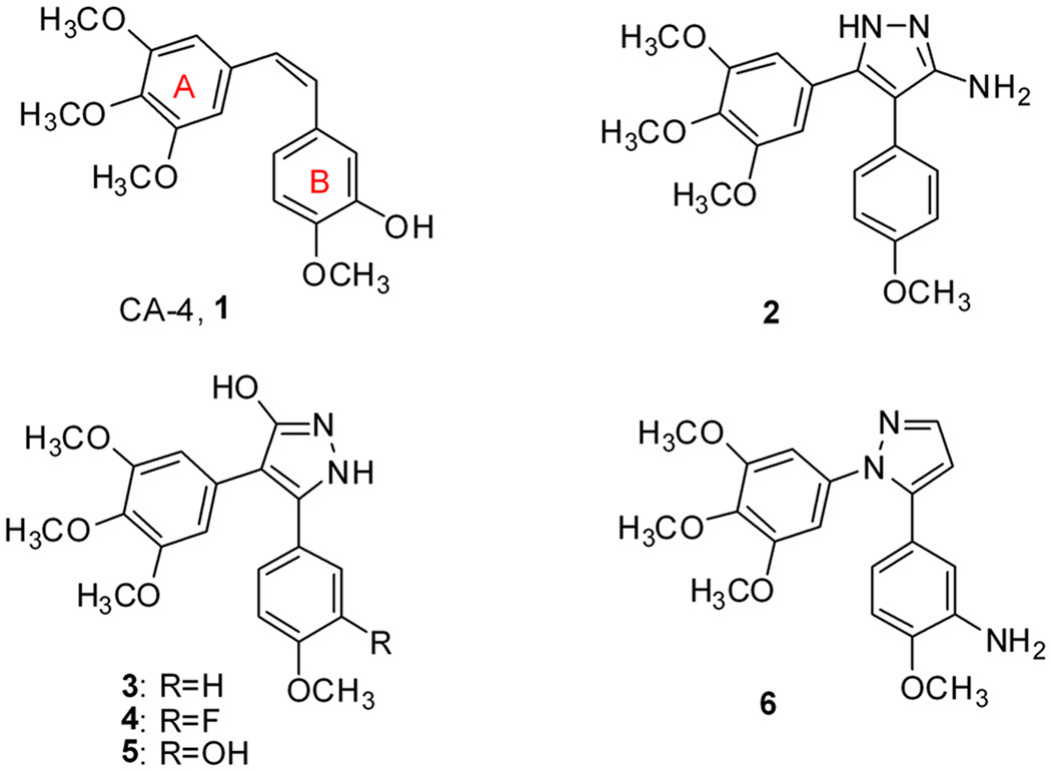
Structures of CA-4 and some pyrazole derivatives.

**Fig 2:**
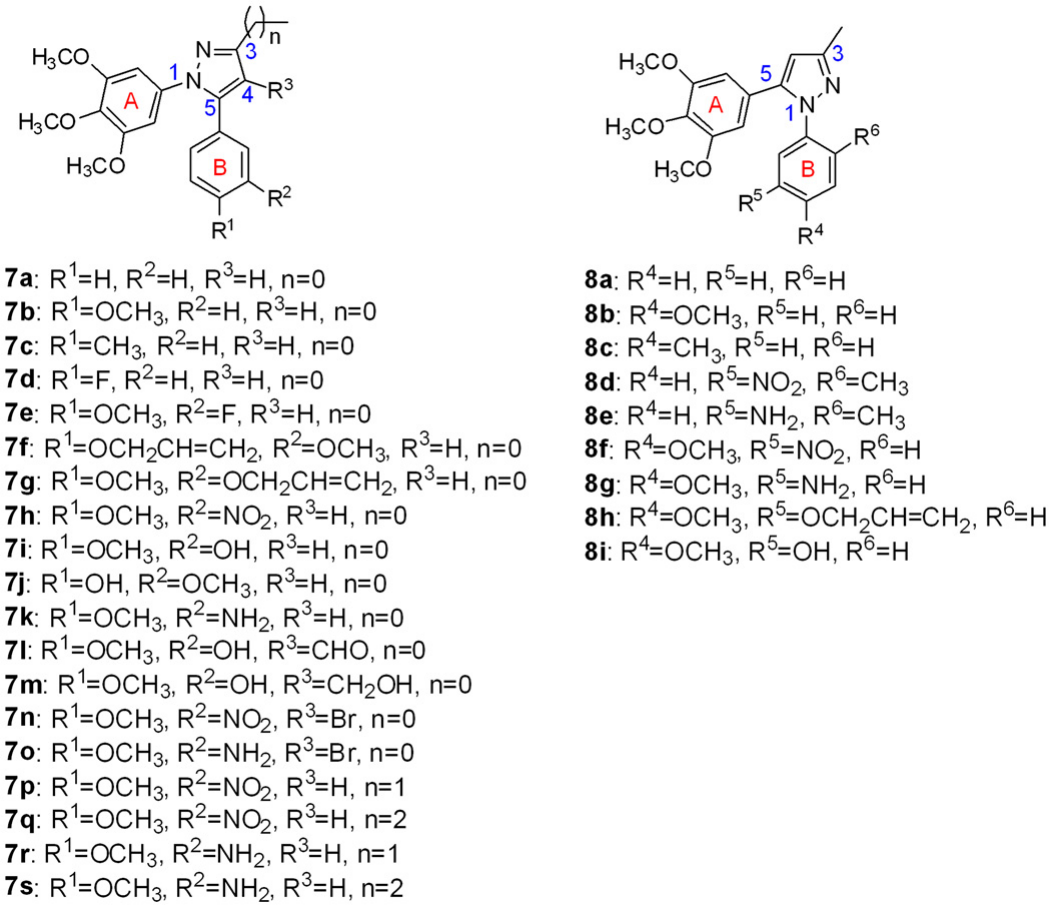
Structures of the synthesized pyrazole derivatives.

To date, a variety of five-membered heterocyclic analogues of **1**, such as oxazoles [13], isoxazoles [14], imidazoles and triazoles [15,16], have been reported as *cis*-restricted, biologically active analogues of CA-4. Because pyrazoles play an important role among biologically active heterocyclics [17–19], several pyrazole-based CA-4 analogues (Fig 1) such as 3-substituted-4,5-diaryl-pyrazoles (**2**, **3**, **4** and **5**) and 1,5-diaryl-pyrazole (**6**) were synthesized and evaluated [15,20,21]. Herein, we present the synthesis and antiproliferative activity of 4-(un)substituted-3-alkyl-1,5-diaryl-pyrazoles (**7a**-**s**) with the A-ring on the 1-position and 3-methyl-1,5-diaryl-pyrazoles (**8a**-**i**) with the A-ring on the 5-position, as analogues of combretastatin A-4 (Fig 2). The representative compounds **7i** and **7k** were investigated for their inhibition of tubulin polymerization. In addition, computational modelling studies have been performed to understand the interaction between tubulin and the most active inhibitor **7k**.

## Results and Discussion

### Chemistry

**Fig 3:**
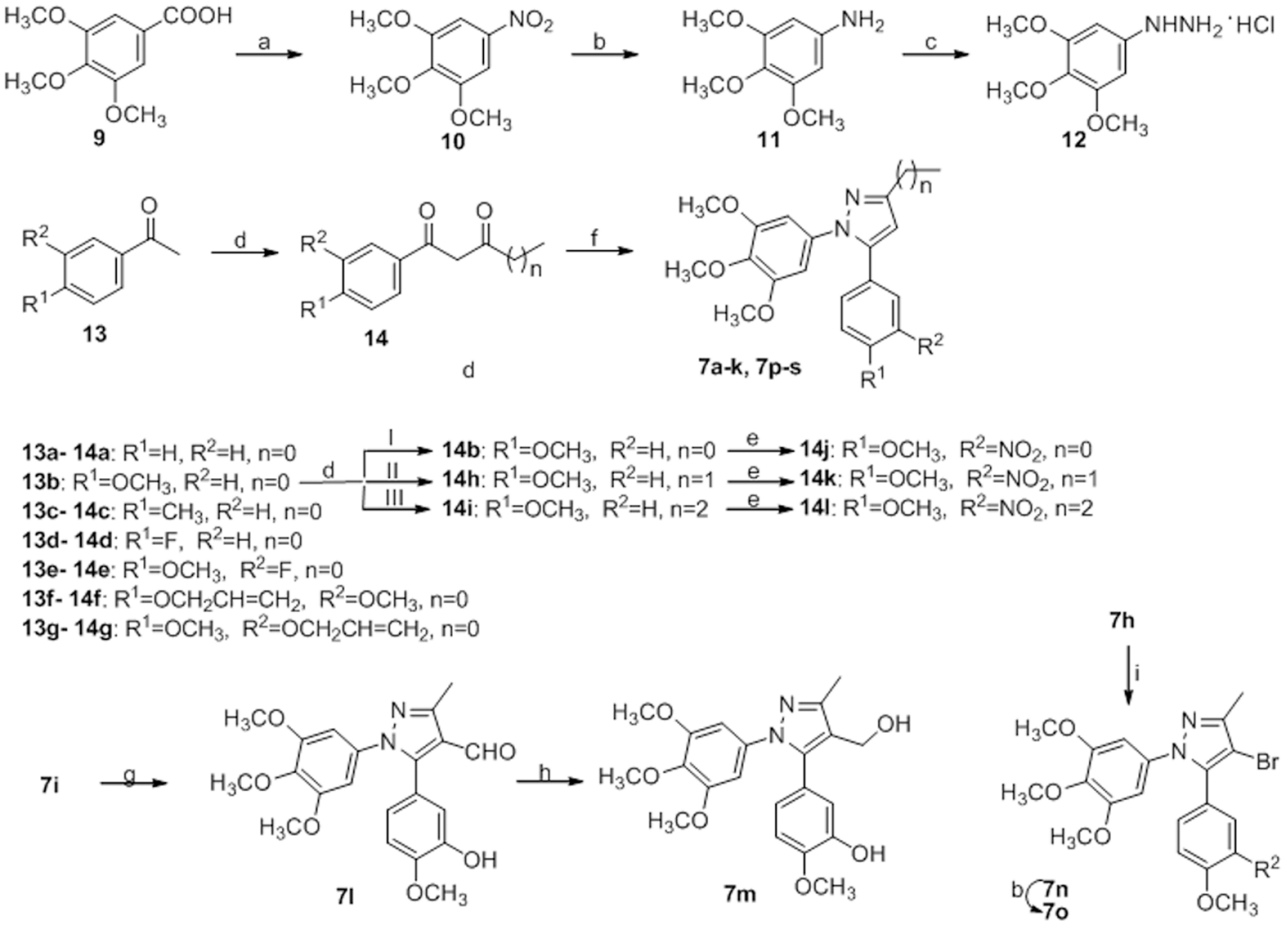
Reagents and conditions. (a) HNO_3_, AcOH, 40°C, 2 h; (b) NH_2_NH_2_·H_2_O, FeCl_3_, activated carbon, MeOH, reflux, 1 h; (c) i) NaNO_2_, HCl, H_2_O, 0–4°C; ii) SnCl_2_·2H_2_O, HCl; (d) I) ethyl acetate, Na, reflux, 1 h; II) ethyl propionate, Na, reflux, 1h; III) ethyl butanoate, Na, reflux, 1 h; (e) KNO_3_, H_2_SO_4_, 0°C, 3 h; (f) **12**, NaOAc, EtOH, reflux, 2 h; (g) POCl_3_, DMF, 0–60°C, 3 h; (h) NaBH_4_, MeOH, r.t., 2 h; (i) NBS, CCl_4_, 1.5 h.

The synthesis of target pyrazole derivatives **7a**-**s** were performed according to Fig 3. 3,4,5-Trimethoxy-benzoic acid (**9**) was treated with concentrated nitric acid in acetic acid at 40°C to yield 1,2,3-trimethoxy-5-nitrobenzene (**10**), which was then reduced to amine **11** with hydrazine, ferric chloride hexahydrate and activated carbon in methanol at 65°C [22,23]. 3,4,5-Trimethoxy-phenylhydrazine hydrochloride (**12**) was prepared from **11** *via* diazotization with sodium nitrite, followed by reduction with stannous chloride dihydrate [24]. Compounds **14a**-**g** were prepared *via* a Claisen condensation between **13a**-**g** and ethyl acetate, ethyl propionate or ethyl butanoate in the presence of sodium [25]. After that compounds **14b**, **14h**, **14i** were nitrated to give compounds **14j-l**. Subsequently, target compounds **7a-h** and **7p-q** were prepared in excellent yield by treating 1-benzoylacetone **14** with compound **12** and sodium acetate trihydrate in ethanol under reflux [26]. Dealkylation of compounds **7f** and **7g** with titanium tetrachloride afforded, respectively the phenol derivatives, **7j** and **7i** [27]. Reduction of the nitro group of **7h**, **7p** and **7q** in a mixture of hydrazine hydrate, ferric chloride hexahydrate and activated carbon in methanol provided the corresponding **7k**, **7r** and **7s**. Finally, **7i** was formylated according to the Vilsmeier-Haack protocol with phosphorus oxychloride and dimethylformamide, and the resulting aldehyde **7l** was reduced to the alcohol **7m** using sodium borohydride in methanol at room temperature [28]. A mixture of **7h** and NBS in CCl_4_ was stirred at room temperature to give **7n** followed by reduction of the nitro group to form the desired compound **7o** [29].

**Fig 4:**
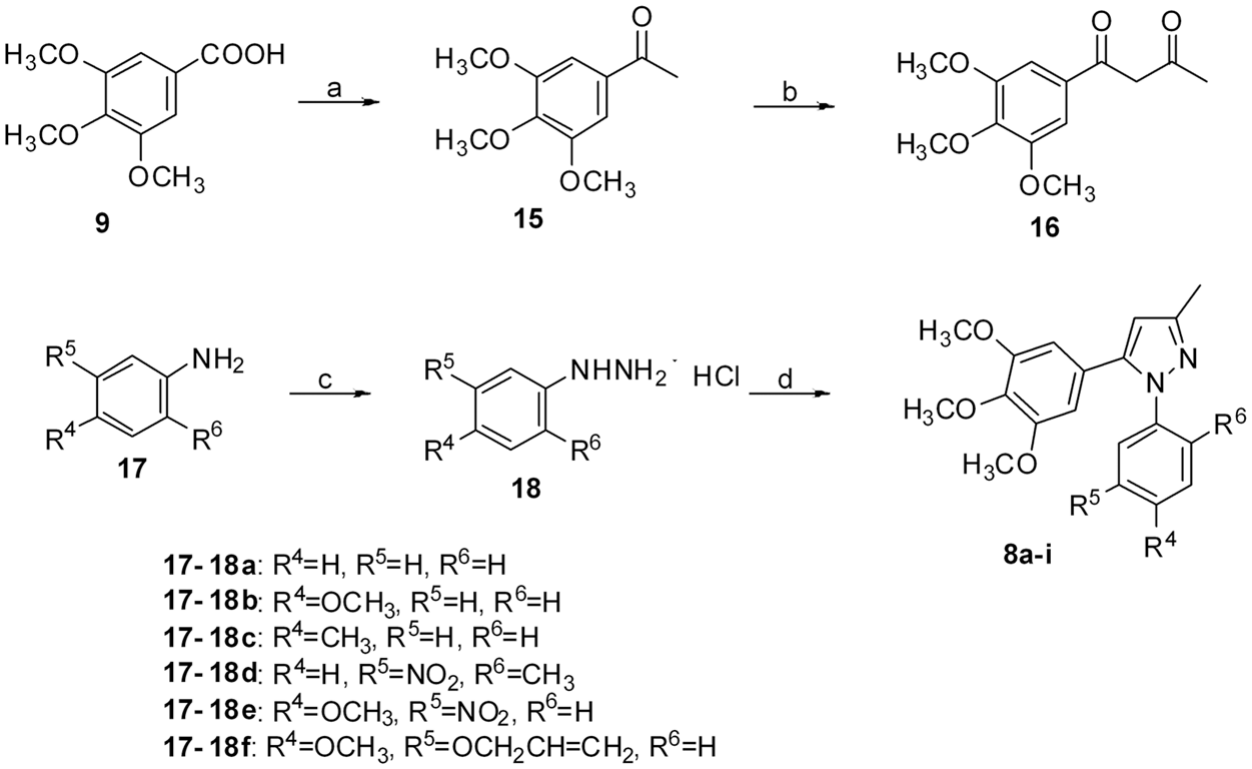
Reagents and conditions. (a) MeLi, THF, -20°C, 3 h; (b) ethyl acetate, Na, reflux, 1 h; (c) i) NaNO_2_, HCl, H_2_O, 0–4°C; ii) SnCl_2_·2H_2_O, HCl; (d) **16**, NaOAc, EtOH, reflux, 2 h.

The target compounds **8a**-**i** were prepared as shown in Fig 4. 3,4,5-Trimethoxyphenyl-ethanone (**15**) [30]. The synthesis of **16**, **18** and **8a**-**i** was accomplished according to the chemistry described above with the corresponding starting materials.

### Biological activities

#### In vitro antiproliferative activities

All of the target compounds, **7a-s** and **8a-i**, along with the positive control, CA-4 (**1**), were evaluated for their antiproliferative activity against three human cancer cell lines: fibrosarcoma cell line HT-1080, gastric adenocarcinoma cell line SGC-7901 and pulmonary adenocarcinoma cell line A549. The majority of synthesized compounds displayed antiproliferative activity with IC_50_ values in the sub-micromolar to micromolar range. The IC_50_ values of the compounds are shown in Table 1.

**Table 1:** Antiproliferative activity of compounds 7a-s, 8a-i and CA-4 (1).

	Antiproliferative activity (IC_50_± SD, μM) ^a^		
Compd	HT-1080	SGC-7901	A549
**7a**	>100	>100	>100
**7b**	1.01 ± 0.12	0.73 ± 0.09	15.2 ± 2.2
**7c**	1.23 ± 0.15	0.53 ± 0.20	2.35 ± 0.33
**7d**	>100	20.7 ± 1.2	>100
**7e**	5.52 ± 0.26	3.05 ± 0.33	2.84 ± 0.52
**7f**	>100	23.6 ± 2.3	>100
**7g**	>100	6.45 ± 0.22	>100
**7h**	>100	5.22 ± 0.19	>100
**7i**	0.93 ± 0.13	0.275 ± 0.020	1.12 ± 0.12
**7j**	>100	25.9 ± 2.3	>100
**7k**	0.122 ± 0.013	0.076 ± 0.009	0.096 ± 0.011
**7l**	23.8 ± 1.8	16.4 ± 1.5	45.7 ± 3.3
**7m**	7.11 ± 0.23	8.42 ± 0.32	9.04 ± 0.25
**7n**	>100	>100	>100
**7o**	15.9 ± 3.2	14.3 ± 3.1	79.1 ± 2.0
**7p**	>100	>100	>100
**7q**	>100	>100	>100
**7r**	8.16 ± 0.12	4.62 ± 0.09	3.55 ± 0.09
**7s**	23.2 ± 0.11	35.7 ± 0.08	56.6 ± 1.0
**8a**	>100	>100	>100
**8b**	28.2 ± 1.9	19.0 ± 2.0	9.39 ± 0.21
**8c**	15.6 ± 1.5	11.8 ± 1.2	16.2 ± 1.0
**8d**	>100	31.1 ± 2.5	48.6 ± 1.1
**8e**	11.9 ± 1.9	2.43 ± 0.09	28.2 ± 1.7
**8f**	>100	>100	>100
**8g**	24.7 ± 2.3	21.2 ± 1.6	21.4 ± 1.2
**8h**	>100	>100	>100
**8i**	15.0 ± 0.9	13.7 ± 1.2	21.8 ± 2.1
**1^b^**	0.023 ± 0.012	0.016 ± 0.010	0.035 ± 0.009

^a^ IC_50_: Concentration of the compound (μM) producing 50% cell growth inhibition after 72 h of drug exposure, as determined by the MTT assay. Each experiment was carried out in triplicate. ^b^ Used as a positive control.

As given in Table 1, compound **7k** exhibited the most potent antiproliferative activity, with IC_50_ values between 0.076 and 0.12 μM against the three cancer cell lines. CA-4 (**1**), the positive control, gave IC_50_ values of 0.016–0.035 μM. Compounds **7b**, **7c** and **7i** also significantly inhibited the growth of two or three cell lines at sub-micromolar concentrations. A comparison of the IC_50_ values for **7a**-**k** and **8a**-**i** revealed that compounds with a trimethoxyphenyl A-ring at the N-1 position of the pyrazole skeleton were more potent than those with the A-ring at the C-5 position. With the A-ring tethered to the N-1 position of the pyrazole ring, analogues monosubstituted at the *para* position of the B-ring (**7b**, **7c** and **7d**) were found to have slightly increased activity compared to the unsubstituted compound (**7a**). To study the effect of disubstitution on the B-ring, alkoxy, amino and hydroxy groups were introduced in the *para* and *meta* positions (**7e**-**k**). Interestingly, the 3-amino-4-methoxy-substituted compound **7k** exhibited the most potent antiproliferative activity; however, the 3-hydroxy-4-methoxy-substituted compound **7i**, which most closely resembles CA-4 (**1**), displayed only moderate activity. Furthermore, introduction of a formyl (**7l**), hydroxymethyl (**7m**) or bromo (**7o**) group at the C-4 position of the pyrazole skeleton resulted in reduced activity compared to the corresponding compounds (**7i** and **7k**). Additionally, replacement of the methyl group (**7k**) with an ethyl (**7r**) or a propyl group (**7s**) at C-3 position of the pyrazole ring tended to great reduce the activity. These data indicate that a large group (such as an ethyl or a propyl group) at C-3 position of the pyrazole skeleton was not tolerated in this series.

#### Inhibition of tubulin polymerization activity

**Fig 5:**
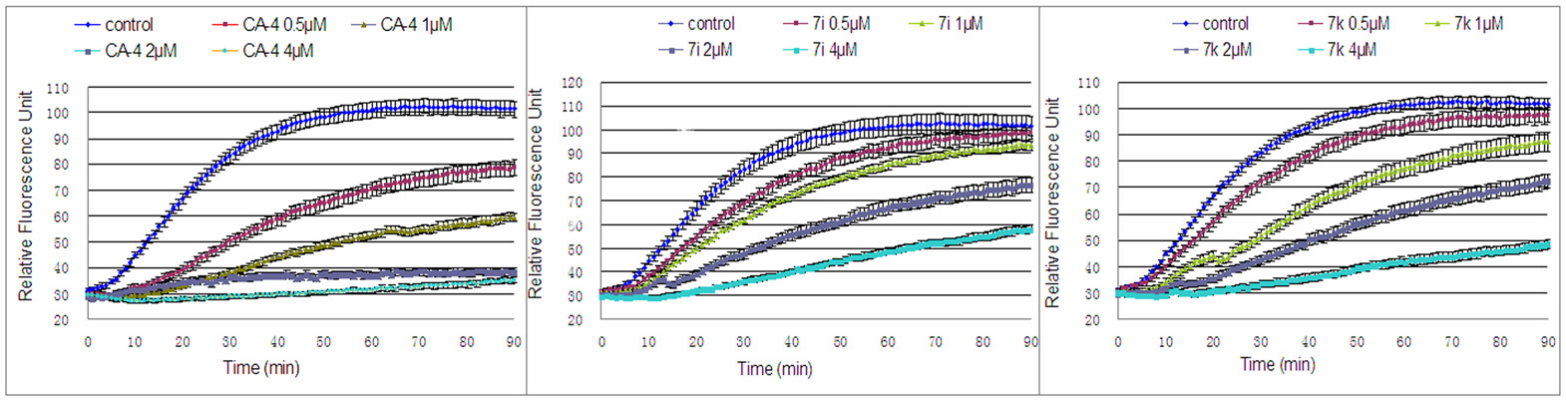
Dose-response effects of CA-4 (1), 7i and 7k on the inhibition of tubulin polymerization. Purified bovine tubulin and GTP were mixed in a 96-well plate. The reaction was initiated by warming the solution from 4°C to 37°C. DMSO was used as a vehicle control. The effect on the assembly of tubulin was monitored using a plate reader at 1 min intervals for 90 min at 37°C.

To investigate whether the antiproliferative activity of the target compounds was related to their interaction with tubulin, the most active compound **7k** and the moderately active **7i** were evaluated for their inhibition of tubulin polymerization in a cell-free in vitro assay. The assay used the polymerization suppressor **1** as a positive control. As shown in Fig 5, compounds **7i** and **7k**, as well as **1**, inhibited tubulin polymerization in a dose-dependent manner. Compound **7k** (IC_50_ = 1.7 μM) was slightly less active than the reference compound **1** (IC_50_ = 0.9 μM). Compound **7i**, which exhibited less antiproliferative activity than **7k**, also showed lower potency as an inhibitor of tubulin polymerization (IC_50_ = 2.5 μM). An excellent correlation was observed between the antiproliferative activity and the inhibition of tubulin polymerization for **7i** and **7k**, indicating that the molecular target of this series of combretastatin A-4 analogues was most likely tubulin.

#### Immunofluorenscence studies

**Fig 6:**
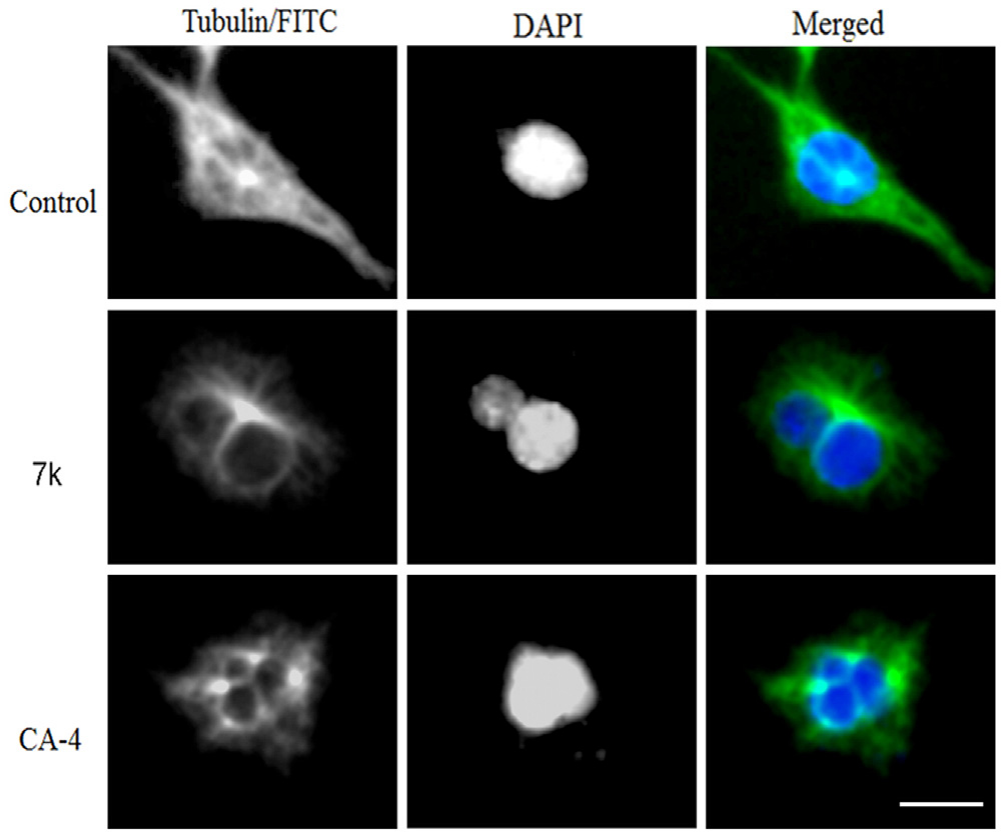
Immunostaining of tubulin assembly in HT-1080 cells. HT-1080 cells were treated with **7k** (0.18 μM) for 48 h (scale bar = 10 μm). The left, middle and right panels represent tubulin assembly stained with FITC, DAPI and a merge of the corresponding left and middle panels, respectively. Images were taken using a confocal microscope.

**Fig 7:**
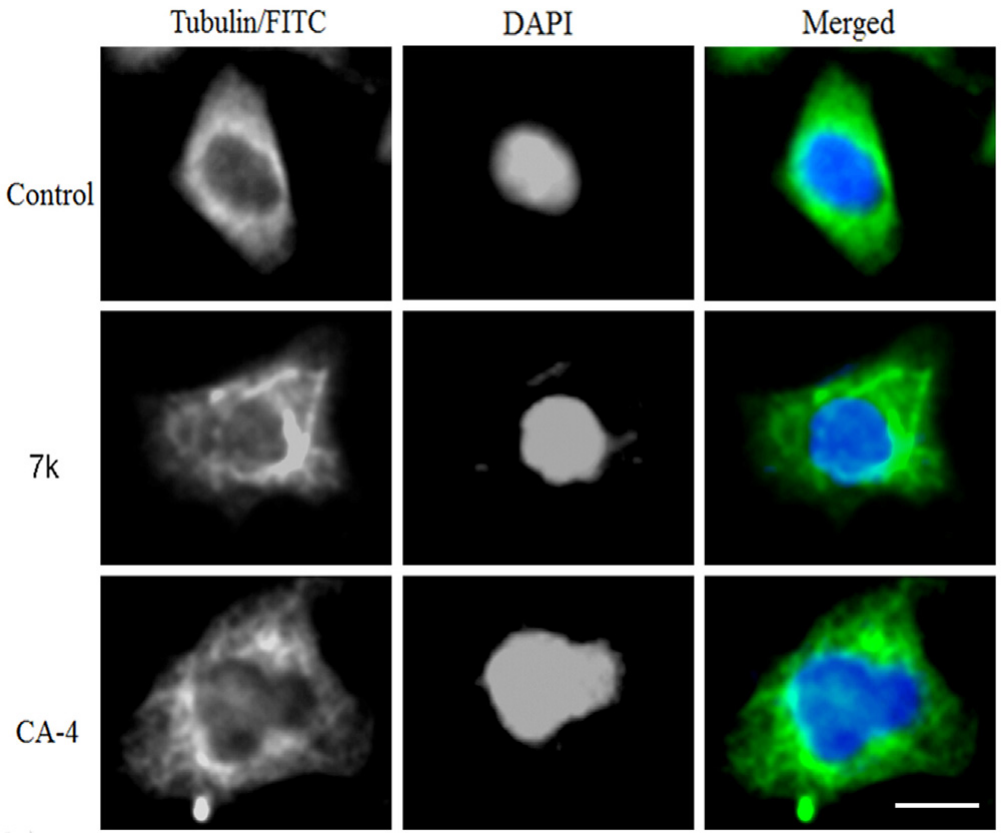
Immunostaining of tubulin assembly in SGC-7901 cells. SGC-7901 cells were treated with **7k** (0.14 μM) for 48 h (scale bar = 10 μm). The left, middle and right panels represent the tubulin assembly stained with FITC, DAPI and a merge of the corresponding left and middle panels, respectively. Images were taken using a confocal microscope.

To demonstrate the effect of inhibition of tubulin polymerization in cells, we investigated microtubule structure and distribution in cultured HT-1080 and SGC-7901 cells. Microtubules were labelled using the indirect immuno-fluorescence method and were analysed by fluorescence microscopy. Cells treated with DMSO (control cells) stained with a tubulin monoclonal antibody demonstrated a well-organised microtubule network throughout the cells (Figs 6 and 7). In contrast, cells treated with **1** and **7k** (at their respective 2-fold IC_50_ concentrations, respectively) showed a disruption of the tubulin network and the appearance of a bundle at a pole of the cell (Figs 6 and 7). Taken together, these results confirm that tubulin is the molecular target of compound **7k**. Comparing the tubulin inhibition with the corresponding antiproliferative activity revealed a correlation, and it also suggests that tubulin is the target for this series of combretastatin A-4 analogues.

### Molecular modelling

**Fig 8:**
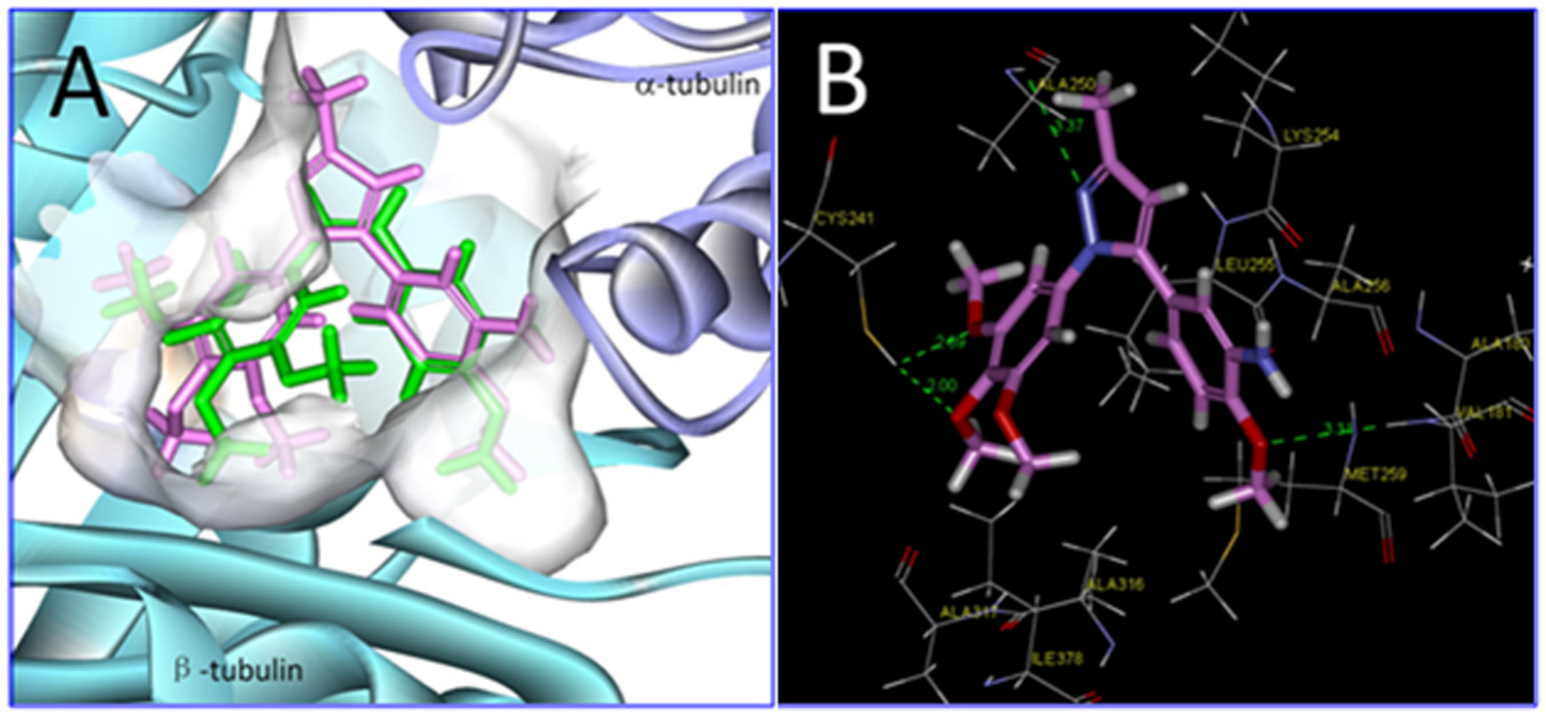
(A) tubulin is displayed as a flat ribbon with *α*-tubulin coloured lavender and *β*-tubulin coloured cyan. Superimposition of the presumptive conformation of active **7k** (pink model) with that of **1** (green model). The structures were docked into the colchicine binding site of tubulin (PDB ID: 1SA0). (B) the amino acids of tubulin within 5.0 Å of colchicine are shown as lines models. Hydrogen bonds (distance: <3.5 Å) are shown as dotted line.

To further expand the understanding of our experimental findings, molecular modelling studies on **7k** and **1** were conducted using CDOCKER within the Discovery Studio 3.0 programme package. Compounds **7k** and **1** were docked into the colchicine binding site of tubulin (PDB ID: 1SA0). In the CDOCKER protocol run results, we report the energy as a score, so the higher (more positive) value indicates a more favorable binding. The “-CDOCKER Interaction Energy” are 48.63 and 46.83 kcal/mol for CA-4 and **7k** respectively. The binding energies correlate with the experimental IC_50_ values of CA-4 and **7k**. These studies also showed that **7k**, the most active compound, can occupy the colchicine binding site of tubulin, as can **1** (Fig 8A). Indeed, the trimethoxyphenyl moiety in the A-rings of **1** and **7k** was buried in the *β* subunit binding cavity. The thiol group of Cys *β*241 formed a hydrogen bond with the oxygen atom of the *para* methoxy group, and formed another hydrogen bond to the oxygen atom of the *meta* methoxy group (Fig 8B). Several amino acids of *β*-tubulin formed hydrophobic interactions with the trimethoxyphenyl moiety of **7k**. The methoxy oxygen atom in ring B of **7k** formed a hydrogen bond with the main chain nitrogen atom of Val *α*181. Additionally, there is direct bond between compound **7k** and the Ala *β*250 residue. Thus, the results of this docking study are in good agreement with the potent antiproliferative activity of **7k** and its ability to inhibit tubulin polymerization.

## Conclusions

In summary, a series of 3-alkyl-1,5-diaryl-1*H*-pyrazoles were synthesized and evaluated for antiproliferative activity and tubulin polymerization inhibition. The majority of synthesized compounds displayed moderate to potent antiproliferative activity. Structure-activity relationships indicated that compounds with a 3,4,5-trimethoxyphenyl A-ring at the N-1 position of the pyrazole skeleton were more potent than those with the A-ring at the C-5 position. Among the synthesized compounds, **7k** showed the most potent antiproliferative activity against SGC-7901 cells with an IC_50_ value of 0.076 μM. Consistent with its antiproliferative activity, **7k** also exhibited potent antitubulin activity (IC_50_ = 1.7 μM), similar to that of CA-4 (**1**). In addition, **7k** also strongly affected cell morphology and microtubule networking comparable to CA-4 (**1**). Molecular docking analysis of the binding conformation of **7k** into the tubulin colchicine binding site showed hydrogen bonds and hydrophobic interactions with protein residues, which may be responsible for the antitubulin polymerization and antiproliferative activities. These results may be helpful for the future design of structurally related tubulin inhibitors.

## Experimental Section

### Synthesis

Unless otherwise noted, all of the materials were obtained from commercially available sources and were used without purification. The progress of reactions was monitored by TLC using silica gel plates (250 μm, F-254) under UV light. The purification of products was performed using column chromatography (60 Å, 200–300 mesh, Qingdao Ocean Chemicals) or thin layer chromatography on silica gel plates (0.25 mm layer, Qingdao Ocean Chemicals) with the designated solvents. Melting points were measured on a hot stage microscope (X-4, Beijing Taike Ltd.) and are uncorrected. Mass spectra (MS) were measured on an Agilent 1100-sl mass spectrometer with an electrospray ionisation source. NMR spectra were recorded on a Bruker AVANCE 400 (^1^H, 400 MHz; ^13^C, 100 MHz) in CDCl_3_ (S1 File). Chemical shifts are expressed as parts per million downfield from tetramethylsilane. Splitting patterns have been designated as follows: s (singlet), d (doublet), dd (doublet of doublets), t (triplet) and m (multiplet).

#### 1,2,3-Trimethoxy-5-nitrobenzene (10)

A flask immersed in a room temperature oil bath was charged with **9** (50 g, 0.235 mol) and acetic acid (150 mL) and heated to 40°C. Over a 10 min period, HNO_3_ (70%, 33 mL) was added dropwise with stirring. The deep orange solution was stirred for an additional 120 min. The reaction was quenched upon addition of 200 g of ice. A yellow precipitate formed, which was filtered and washed with H_2_O. The crude product was recrystallized from methanol to give pure **10.** Yield: 66%.

#### 3,4,5-Trimethoxyaniline (11)

A mixture of 1,2,3-trimethoxy-5-nitrobenzene **10** (32.7 g, 0.153 mol), activated carbon (3.27 g, 50–200 mesh, Fisher Scientific Co.), ferric chloride hexahydrate (3.27 g) and methanol (250 mL) was refluxed for 10 min with stirring. Hydrazine hydrate (80%, 65 mL) was added over 30 min to the boiling solution. The mixture was stirred under reflux for an additional 1 h, cooled and evaporated. The resulting slurry was dissolved in dichloromethane (250 mL), washed with water (2 × 100 mL) and dried (Na_2_SO_4_). Evaporation of the solvent gave amine **11**, which was used without further purification. Yield: 95%.

#### 1-(3,4,5-Trimethoxyphenyl)butane-1,3-dione (16)

Methyl-lithium (1.3M in diethyl ether, 39.8 mL, 51.8 mmol) was added dropwise to a solution of 3,4,5-trimethoxybenzoic acid **9** (5 g, 23.6 mmol) in anhydrous THF (50 mL) at -20°C under a nitrogen atmosphere. The mixture was stirred at -20°C for 1 h, warmed to room temperature and stirred for an additional 3 h. The solvent was removed, and the residue was dissolved in excess CH_2_Cl_2_. The solution was washed with H_2_SO_4_ (0.1N, 80 mL) and deionised water (3 × 30 mL), dried over anhydrous Na_2_SO_4_, filtered and dried under vacuum. The product **15** was used without further purification. Yield: 91%. A mixture of 3,4,5-trimethoxyacetophenone **15** (4.5 g, 21.4 mmol) in freshly purified anhydrous ethyl acetate (50 mL) was added to sodium sand (0.99 g, 42.8 mmol) under a nitrogen atmosphere. The mixture was stirred, and upon the initiation of an exothermic reaction, the remainder of the ketone solution was added dropwise at such a rate as to maintain refluxing. Stirring and refluxing were continued for another 120 min. The reaction mixture was diluted with water (100 mL) and extracted with ethyl acetate (3 × 100 mL). The combined organic phases were washed with water, dried over Na_2_SO_4_, filtered and concentrated under vacuum. The crude product was purified by chromatography on silica gel to afford the desired **16.** Yield: 90%; white solid; m.p. 86–87°C; ^1^H-NMR (400 MHz, CDCl_3_): δ 2.20 (3H, s), 3.93 (9H, s), 6.12 (1H, s), 7.13 (2H, s), minor tautomer inter alia 2.29 (3H, s), 3.93 (9H, s), 4.07 (2H, s), 7.22 (2H, s).

#### 1-Phenylbutane-1,3-dione (14a)

This compound was obtained from acetophenone **13a** by following the procedure described above for 16. Yield: 87%; yellow solid; m.p. 53–54°C; ^1^H-NMR (400 MHz, CDCl_3_): δ 2.20 (3H, s), 6.18 (1H, s), 7.44–7.52 (3H, m), 7.87–7.88 (2H, d, *J* = 7.26 Hz), 16.15 (1H, s), minor tautomer inter alia 2.30 (3H, s), 4.10 (2H, s), 7.44–7.52 (3H, m), 7.93–7.95 (2H, d, *J* = 7.26 Hz).

#### 1-(4-Methoxyphenyl)butane-1,3-dione (14b)

This compound was obtained from 1-(4-methoxyphenyl)ethanone **13b** by following the procedure described above for **16**. Yield: 88%; orange-red solid; m.p. 49–50°C; ^1^H-NMR (400 MHz, CDCl_3_): δ 2.17 (3H, s), 3.86 (3H, s), 6.11 (1H, s), 6.94 (2H, d, *J* = 8.37 Hz), 7.86 (2H, d, *J* = 8.37Hz), minor tautomer inter alia 2.28 (3H, s), 3.86 (3H, s) 4.04 (2H, s), 6.94 (2H, d, *J* = 8.37 Hz).

#### 1-P-tolylbutane-1,3-dione (14c)

This compound was obtained from 1-p-tolylethanone **13c** by following the procedure described above for **16**. Yield: 83%; yellow solid; m.p. 54–55°C; ^1^H-NMR (400 MHz, CDCl_3_): δ 2.16 (3H, s), 2.38 (3H, s), 6.14 (1H, s), 7.22 (2H, d, *J* = 7.24 Hz), 7.76 (2H, d, *J* = 7.24 Hz), minor tautomer inter alia 2.26 (3H, s), 2.38 (3H, s), 4.04 (2H, s), 7.22 (2H, d, *J* = 7.24 Hz), 7.82 (2H, d, *J* = 7.24 Hz).

#### 1-(4-Fluorophenyl)butane-1,3-dione (14d)

This compound was obtained from 1-(4-fluorophenyl)ethanone **13d** by following the procedure described above for 16. Yield: 79%; orange-red solid; m.p. 47–48°C; ^1^H-NMR (400 MHz, CDCl_3_): δ 2.19 (3H, s), 6.13 (1H, s), 7.10–7.15 (2H, m), 7.86–7.92 (2H, m), 16.15 (1H, s), minor tautomer inter alia 2.30 (3H, s), 4.08 (2H, s), 7.10–7.15 (2H, m), 7.94–8.02 (2H, m).

#### 1-(3-Fluoro-4-methoxyphenyl)butane-1,3-dione (14e)

This compound was obtained from 1-(3-fluoro-4-methoxyphenyl) ethanone **13e** by following the procedure described above for **16**. Yield: 78%; pale yellow solid; m.p. 107–108°C; ^1^H-NMR (400 MHz, CDCl_3_): δ 2.18 (3H, s), 3.95 (3H, s), 6.08 (1H, s), 6.97–7.02 (1H, m), 7.61–7.68 (2H, m), minor tautomer inter alia 2.29 (3H, s), 3.95 (3H, s), 4.04 (2H, s), 6.97–7.02 (1H, m), 7.61–7.68 (2H, m).

#### 1-(4-(Allyloxy)-3-methoxyphenyl)butane-1,3-dione (14f)

To a solution of 1-(4-hydroxy-3-methoxyphenyl)ethanone (0.20 g, 1.22 mmol) in dry acetone (20 mL) was added anhydrous K_2_CO_3_ (0.67 g, 4.88 mmol) over 10 min, followed by addition of allyl bromide (0.17 g, 1.34 mmol), and the mixture was stirred under reflux for an additional 3 h. The mixture was cooled and evaporated, and the resulting slurry was dissolved in dichloromethane (30 mL), washed with water (2 × 15 mL), and dried over Na_2_SO_4_. Evaporation of the solvent gave **13f** as colourless oil. The intermediate **14f** was obtained from 1-(4-(allyloxy)-3-methoxyphenyl)-ethanone **13f** by following the procedure described above for **16**. Yield: 73%; pale yellow solid; m.p. 51–52°C; ^1^H-NMR (400 MHz, CDCl_3_): δ 2.16 (3H, s), 3.92 (3H, s), 4.67 (2H, d, *J* = 5.19 Hz), 5.32 (1H, d, *J* = 10.52 Hz), 5.43 (1H, d, *J* = 16.50 Hz), 6.03–6.12 (2H, m), 6.88 (1H, d, *J* = 8.34 Hz), 7.45–7.52 (2H, m), 16.25 (1H, s), minor tautomer inter alia 2.28 (3H, s), 3.93 (3H, s), 4.05 (2H, s), 4.67 (2H, d, *J* = 5.19 Hz), 5.32 (1H, d, *J* = 10.52 Hz), 5.43 (1H, d, *J* = 16.50 Hz), 6.03–6.12 (1H, m), 6.88 (1H, d, *J* = 8.34 Hz), 7.45–7.52 (2H, m).

#### 1-(3-(Allyloxy)-4-methoxyphenyl)butane-1,3-dione (14g)

This compound was obtained from 1-(3-hydroxy-4-methoxyphenyl) ethanone by following the procedure described above for **14f**. Yield: 75%; orange-red solid; m.p. 59–60°C; ^1^H-NMR (400 MHz, CDCl_3_): δ 2.17 (3H, s), 3.93 (3H, s), 4.67 (2H, d, *J* = 5.02 Hz), 5.32 (1H, d, *J* = 10.32 Hz), 5.43 (1H, d, *J* = 16.56 Hz), 6.06–6.14 (2H, m), 6.90 (1H, d, *J* = 8.27 Hz), 7.47–7.59 (2H, m), minor tautomer inter alia 2.29 (3H, s), 3.93 (3H, s), 4.04 (2H, s) 4.67 (2H, d, *J* = 5.02 Hz), 5.32 (1H, d, *J* = 10.32 Hz), 5.43 (1H, d, *J* = 16.56 Hz), 6.06–6.14 (1H, m), 6.90 (1H, d, *J* = 8.27 Hz), 7.47–7.59 (2H, m).

#### 1-(4-Methoxy-3-nitrophenyl)butane-1,3-dione (14j)

1-(4-Methoxyphenyl)butane-1,3-dione (5.56 g, 0.192 mol) was dissolved in 100 mL of coned. sulfuric acid at laboratory temperature and the solution cooled to 0°C. A solid of KNO_3_ (3.27 g, 0.101 mol) was sollowly added with 20 min. The deep orange solution was stirred for an additional 160 min. The reaction was quenched upon addition of 200 g of ice. A yellow precipitate formed, which was filtered and washed with H_2_O. The crude product was recrystallized from methanol to give pure **14j.** Yield: 85%; brown solid; m.p. 109–110°C; ^1^H-NMR (400 MHz, CDCl_3_): δ 2.21 (3H, s), 4.04 (3H, s), 6.14 (1H, s), 7.16 (1H, d, *J* = 8.76 Hz), 8.10 (1H, d, *J* = 8.76 Hz), 8.36 (1H, s), 16.06 (1H, s).

#### 3-Methyl-5-phenyl-1-(3,4,5-trimethoxyphenyl)-1*H*-pyrazole (7a)

(3,4,5-Trimethoxyphenyl)hydrazine hydrochloride (0.14 g, 0.617 mmol), 1-benzoylacetone (0.10 g, 0.617 mmol) and NaOAc (0.05 g, 0.074 mmol) were combined in EtOH (50 mL). The reaction was refluxed for 1 h, cooled to room temperature concentrated in vacuo. The resulting slurry was dissolved in dichloromethane (50 mL), washed with water (2 × 30 mL), dried over Na_2_SO_4_, filtered and concentrated under vacuum. The crude product was purified by chromatography on silica gel to afford the desired 5-phenyl-1-(3,4,5-trimethoxyphenyl)- 3-methyl-1H-pyrazole **7a**. Yield: 74%; yellow solid; m.p. 77–78°C; ^1^H-NMR (400 MHz, CDCl_3_): δ 2.41 (3H, s), 3.66 (6H, s), 3.83 (3H, s), 6.32 (1H, s), 6.50 (2H, s), 7.24–7.28 (2H, m), 7.31–7.32 (3H, m); ^13^C-NMR (100 MHz, CDCl_3_): δ 13.5, 56.0 (2C), 60.9, 102.7 (2C), 107.6, 128.2, 128.4 (2C), 128.7 (2C), 130.8, 135.6, 137.0, 143.8, 149.2, 153.0 (2C); ESI-MS: m/z = 325.1[M+H]^+^, 649.3[2M+H]^+^, 671.3 [2M+Na]^+^.

#### 3-Methyl-5-(4-methoxyphenyl)-1-(3,4,5-trimethoxy-phenyl)-1*H*-pyrazole (7b)

The title compound was obtained according to the procedure described above for **7a**. Yield: 76%; pale yellow solid; m.p. 118–119°C; ^1^H-NMR (400 MHz, CDCl_3_): δ 2.42 (3H, s), 3.70 (6H, s), 3.80 (3H, s), 3.83 (3H, s), 6.27 (1H, s), 6.52 (2H, s), 6.85 (2H, d, *J* = 8.60 Hz), 7.18 (2H, d, *J* = 8.60 Hz); ^13^C-NMR (100 MHz, CDCl_3_): δ 13.6, 55.3, 56.0 (2C), 61.0, 102.9 (2C), 107.0, 113.8 (2C), 123.2, 129.9 (2C), 135.9, 136.9, 143.6, 149.2, 153.0 (2C), 159.5; ESI-MS: m/z = 355.1 [M+H]^+^, 731.3 [2M+Na]^+^.

#### 3-Methyl-5-(4-methylphenyl)-1-(3,4,5-trimethoxy-phenyl)-1*H*-pyrazole (7c)

The title compound was obtained according to the procedure described above for **7a**. Yield: 79%; brown liquid; ^1^H-NMR (400 MHz, CDCl_3_): δ 2.33 (3H, s), 2.38 (3H, s), 3.67 (6H, s), 3.83 (3H, s), 6.27 (1H, s), 6.50 (2H, s), 7.11 (2H, d, *J* = 8.28 Hz), 7.14 (2H, d, *J* = 8.28 Hz); ^13^C-NMR (100 MHz, CDCl_3_): δ 13.5, 21.1, 56.0 (2C), 60.9, 102.8 (2C), 107.3, 127.9, 128.5 (2C), 129.0 (2C), 135.9, 137.0, 138.0, 143.8, 149.1, 153.0 (2C); ESI-MS: m/z = 339.2 [M+H]^+^, 677.3 [2M+H]^+^, 699.3 [2M+Na]^+^.

#### 3-Methyl-5-(4-fluorophenyl)-1-(3,4,5-trimethoxyphenyl)-1*H*-pyrazole (7d)

The title compound was obtained according to the procedure described above for **7a**. Yield: 83%; yellow solid; m.p. 104–105°C; ^1^H-NMR (400 MHz, CDCl_3_): δ 2.38 (3H, s), 3.69 (6H, s), 3.83 (3H, s), 6.28 (1H, s), 6.47 (2H, s), 6.98–7.04 (2H, m), 7.21–7.25 (2H, m); ^13^C-NMR (100 MHz, CDCl_3_): δ 13.4, 56.1 (2C), 61.0, 102.9 (2C), 107.6, 115.5 (2C, d, *J* = 21.9 Hz), 126.7, 130.5 (2C, d, *J* = 8.3 Hz), 135.3, 137.3, 142.8, 149.2, 153.2 (2C), 161.6 (d, *J* = 249.0 Hz); ESI-MS: m/z = 343.1 [M+H]^+^, 707.3 [2M+Na]^+^.

#### 3-Methyl-5-(3-fluoro-4-methoxyphenyl)-1-(3,4,5-trimethoxyphenyl)-1*H*-pyrazole (7e)

The title compound was obtained according to the procedure described above for **7a**. Yield: 78%; brown solid; m.p. 86–87°C; ^1^H-NMR (400 MHz, CDCl3): δ 2.38 (3H, s), 3.71 (6H, s), 3.84 (3H, s), 3.88 (3H, s), 6.27 (1H, s), 6.50 (2H, s), 6.86–6.90 (1H, m), 6.93–6.95 (1H, m), 7.00–7.03 (1H, m); ^13^C-NMR (100 MHz, CDCl_3_): δ 13.5, 56.1 (2C), 56.2, 60.9, 103.0 (2C), 107.3, 113.1, 116.3 (d, *J* = 19.54 Hz), 123.5 (d, *J* = 6.73 Hz), 124.7 (d, *J* = 3.56 Hz), 135.7, 137.3, 142.3, 147.5 (d, *J* = 10.23 Hz), 149.2, 151.6 (d, *J* = 246.92 Hz), 153.2 (2C); ESI-MS: m/z = 373.2 [M+H]^+^, 745.3 [2M+H]^+^.

#### 3-Methyl-5-(4-allyloxy-3-methoxyphenyl)-1-(3,4,5-trimethoxyphenyl)-1*H*-pyrazole (7f)

The title compound was obtained according to the procedure described above for **7a**. Yield: 80%; white solid; m.p. 78–79°C; ^1^H-NMR (400 MHz, CDCl_3_): δ 2.42 (3H, s), 3.70 (9H, d, *J* = 2.26 Hz), 3.83 (3H, s), 4.60–4.62 (2H, m), 5.28–5.31 (1H, m), 5.37–5.42 (1H, m), 6.01–6.12 (1H, m), 6.31 (1H, s), 6.54 (2H, s), 6.73 (1H, s), 6.82 (2H, s); ^13^C-NMR (100 MHz, CDCl_3_): δ 12.9, 55.9, 56.3 (2C), 61.0, 69.8, 103.3 (2C), 107.1, 112.1, 113.1, 118.3, 120.9, 121.5, 122.1, 132.7, 133.9, 137.8, 144.5, 148.6, 149.2, 153.3 (2C); ESI-MS: m/z = 411.2 [M+H]^+^, 821.4 [2M+H]^+^, 843.4 [2M+Na]^+^.

#### 3-Methyl-5-(3-allyloxy-4-methoxyphenyl)-1-(3,4,5-trimethoxyphenyl)-1*H*-pyrazole (7g)

The title compound was obtained according to the procedure described above for **7a**. Yield: 85%; brown solid; m.p. 79–80°C; ^1^H-NMR (400 MHz, CDCl_3_): δ 2.42 (3H, s), 3.72 (6H, s), 3.85 (3H, s), 3.90 (3H, s), 4.45 (2H, d, *J* = 5.37 Hz), 5.20–5.23 (1H, m), 5.26–5.30 (1H, m), 5.88–5.97 (1H, m), 6.29 (1H, s), 6.53 (2H, s), 6.74 (1H, d, *J* = 1.80 Hz), 6.84 (1H, d, *J* = 8.18 Hz), 6.89 (1H, dd, *J* = 8.18 Hz, *J* = 1.80 Hz); ^13^C-NMR (100 MHz, CDCl_3_): δ 13.4, 56.0, 56.1 (2C), 60.9, 69.8, 103.0 (2C), 107.0, 111.4, 114.0, 118.0, 121.7, 122.9, 132.8, 135.6, 137.2, 143.7, 147.6, 149.1, 149.5, 153.1 (2C); ESI-MS: m/z = 411.2 [M+H]^+^, 821.4 [2M+H]^+^, 843.4 [2M+Na]^+^.

#### 3-Methyl-5-(4-methoxy-3-nitrophenyl)-1-(3,4,5-trimethoxyphenyl)-1*H*-pyrazole (7h)

The title compound was obtained according to the procedure described above for **7a**. Yield: 75%; yellow solid; m.p. 145–146°C; ^1^H-NMR (400 MHz, CDCl_3_): δ 2.41 (3H, s), 3.75 (6H, s), 3.86 (3H, s), 3.97 (3H, s), 6.37 (1H, s), 6.52 (2H, s), 7.01 (1H, d, *J* = 8.82 Hz), 7.33 (1H, dd, *J* = 8.82 Hz, *J* = 2.26 Hz), 7.86(1H, d, *J* = 2.26Hz); ^13^C-NMR (100 MHz, CDCl_3_): δ 13.3, 56.3 (2C), 56.7, 61.0, 103.3 (2C), 107.6, 113.5, 122.8, 125.5, 133.9, 134.8, 137.9, 139.4, 141.2, 149.3, 152.6, 153.4 (2C); ESI-MS: m/z = 400.1 [M+H]^+^, 422.1 [M+Na]^+^, 799.3 [2M+H]^+^, 821.3 [2M+Na]^+^.

#### 3-Methyl-5-(3-hydroxy-4-methoxyphenyl)-1-(3,4,5-trimethoxyphenyl)-1*H*-pyrazole (7i)

TiCl_4_ (0.5 mL) was added to a solution of **7g** (0.2 g, 0.49 mmol) in CH_2_Cl_2_ (20 mL) and the reaction mixture was stirred for 0.5 h at -20°C. The reaction mixture was warmed to room temperature and concentrated in vacuo. The resulting slurry was dissolved in dichloromethane (30 mL), washed with water (2 × 15 mL), dried over Na_2_SO_4_, filtered and concentrated under vacuum. The crude product was purified by chromatography on silica gel to give **7i**. Yield: 93%; pale yellow solid; m.p. 130–131°C; ^1^H-NMR (400 MHz, CDCl_3_): δ 2.37 (3H, s), 3.69 (6H, s), 3.82 (3H, s), 3.86 (3H, s), 6.23 (1H, s), 6.52 (2H, s), 6.68 (1H, dd, *J* = 8.32 Hz, *J* = 2.00 Hz), 6.76 (1H, d, *J* = 8.32 Hz), 6.87 (1H, d, *J* = 2.00 Hz); ^13^C-NMR (100 MHz, CDCl_3_): δ 13.5, 56.0, 56.1 (2C), 60.9, 103.0 (2C), 107.2, 110.5, 115.0, 120.7, 123.8, 135.7, 137.1, 143.6, 145.5, 146.7, 149.1, 153.0 (2C); ESI-MS: m/z = 371.2 [M+H]^+^, 741.3 [2M+H]^+^, 763.3 [2M+Na]^+^.

#### 3-Methyl-5-(4-hydroxy-3-methoxyphenyl)-1-(3,4,5-trimethoxyphenyl)-1*H*-pyrazole (7j)

The title compound was obtained according to the procedure described above for **7i**. Yield: 91%; white solid; m.p. 197–198°C; ^1^H-NMR (400 MHz, CDCl_3_): δ 2.40 (3H, s), 3.71 (9H, s), 3.82 (3H, s), 6.28 (1H, s), 6.53 (2H, s), 6.68 (1H, d, *J* = 1.61 Hz), 6.81 (1H, dd, *J* = 8.28 Hz, *J* = 1.61 Hz), 6.87 (1H, d, *J* = 8.28 Hz); ^13^C-NMR (100 MHz, CDCl_3_): δ 13.5, 55.9, 56.2 (2C), 61.0, 103.0 (2C), 107.0, 111.3, 114.5, 122.1, 122.4, 135.6, 137.2, 143.9, 145.9, 146.3, 149.1, 153.1 (2C); ESI-MS: m/z = 371.2 [M+H]^+^, 741.3 [2M+H]^+^, 763.3 [2M+Na]^+^.

#### 3-Methyl-5-(3-amino-4-methoxyphenyl)-1-(3,4,5-trimethoxyphenyl)-1*H*-pyrazole (7k)

The title compound was obtained according to the procedure described above for **11**. Yield: 95%; pale yellow solid; m.p. 129–130°C; ^1^H-NMR (400 MHz, CDCl_3_): δ 2.36 (3H, s), 3.70 (6H, s), 3.82 (3H, s), 3.83 (3H, s), 3.91 (2H, s), 6.21 (1H, s), 6.54 (2H, s), 6.58 (1H, dd, *J* = 8.28 Hz, *J* = 1.94 Hz), 6.65 (1H, d, *J* = 1.94 Hz), 6.69 (1H, d, *J* = 8.28 Hz); ^13^C-NMR (100 MHz, CDCl_3_): δ 13.5, 55.5, 56.0 (2C), 60.9, 102.7 (2C), 107.1, 110.0, 114.9, 119.0, 123.5, 136.0, 136.1, 136.8, 144.0, 147.2, 149.0, 152.9 (2C); ESI-MS: m/z = 370.1 [M+H]^+^, 739.3 [2M+H]^+^, 761.3 [2M+Na]^+^.

#### 4-Formyl-3-methyl-5-(3-hydroxy-4-methoxyphenyl)-1-(3,4,5-trimethoxyphenyl)-1*H*-pyrazole (7l)

DMF (8 mL) was cooled in an ice bath, and POCl_3_ (1 mL, 10.8 mmol) was added dropwise over 10 min. The mixture was stirred for 30 min at 0°C, followed by dropwise addition of a solution of **7i** (0.06 g, 0.17 mmol) in DMF (2 mL) over 15 min. The ice bath was removed, and the mixture was stirred at 60°C for 3 h. The reaction was quenched upon addition of 30 g of ice. The resulting mixture was extracted with EtOAc (3 × 30 mL), and the extracts were dried over Na_2_SO_4_, concentrated in vacuo and purified by chromatography on silica gel to afford the desired **7l**. Yield: 81%; pale yellow solid; m.p. 97–98°C; ^1^H-NMR (400 MHz, CDCl_3_): δ 2.61 (3H, s), 3.68 (6H, s), 3.82 (3H, s), 3.92 (3H, s), 6.49 (2H, s), 6.80 (1H, dd, *J* = 8.39 Hz, *J* = 1.94 Hz), 6.87–6.89 (2H, m), 9.75 (1H, s); ^13^C-NMR (100 MHz, CDCl_3_): δ 12.7, 55.1 (3C), 60.0, 101.8 (2C), 109.7, 115.4, 118.7, 119.5, 121.6, 133.3, 136.6, 144.9, 146.9, 147.8, 150.0, 152.1 (2C), 185.4; ESI-MS: m/z = 399.1 [M+H]^+^, 421.1 [M+Na]^+^, 699.3 [2M+H]^+^, 761.3 [2M+Na]^+^.

#### 4-Hydroxymethyl-3-methyl-5-(3-hydroxy-4-methoxy-phenyl)-1-(3,4,5-trimethoxyphenyl)-1*H*-pyrazole (7m)

NaBH_4_ (0.02 g, 0.63 mmol) was added with stirring to a suspension of the aldehyde **7l** (0.05 g, 0.13 mmol) in MeOH (20 mL) and the reaction mixture was stirred for 2 h at room temperature. The reaction mixture was concentrated in vacuo. The resulting slurry was dissolved in dichloromethane (40 mL), washed with water (2 × 20 mL), dried over Na_2_SO_4_, filtered and concentrated under vacuum. The crude product was purified by chromatography on silica gel to afford the desired **7m**. Yield: 96%; white solid; m.p. 166–167°C; ^1^H-NMR (400 MHz, CDCl_3_): δ 2.44 (3H, s), 3.67 (6H, s), 3.80 (3H, s), 3.91 (3H, s), 4.52 (2H, s), 6.46 (2H, s), 6.75 (1H, d, *J* = 7.70 Hz), 6.83 (1H, d, *J* = 7.70Hz), 6.88 (1H, s); ^13^C-NMR (100 MHz, CDCl_3_): δ 11.8, 55.0, 56.0 (2C), 58.4, 60.9, 102.5 (2C), 110.6, 116.1, 118.5, 122.1, 123.1, 135.6, 136.9, 142.0, 145.6, 146.9, 148.8, 153.0 (2C); ESI-MS: m/z = 401.1 [M+H]^+^, 801.2 [2M+H]^+^, 823.2 [2M+Na]^+^.

#### 4-Bromo-3-methyl-5-(4-methoxy-3-nitrophenyl)-1-(3,4,5-trimethoxyphenyl)-1*H*-pyrazole (7n)

A mixture of **7h** (0.08 g, 0.20 mmol) and NBS (0.04 g, 0.02 mmol) in CCl_4_ (4 mL) was stirred at room temperature for 1.5 h. The progress of the reaction was monitored by thin layer chromatography (TLC). The by-product succinimide was filtered and washed with CCl_4_ (5 mL). The filtrate was washed with water (2 × 10 mL) and dried over MgSO_4_. The solvent was removed under reduced pressure. The crude product was purified by chromatography on silica gel to afford the desired **7n**. Yield: 88%; pale yellow solid; m.p. 182–183°C; ^1^H-NMR (400 MHz, CDCl_3_): δ 2.39 (3H, s), 3.71 (6H, s), 3.83 (3H, s), 3.99 (3H, s), 6.44 (2H, s), 7.08 (1H, d, *J* = 8.62 Hz), 7.41 (1H, dd, *J* = 8.62 Hz, *J* = 2.36 Hz), 7.92 (1H, d, *J* = 2.36 Hz); ^13^C-NMR (100 MHz, CDCl_3_): δ 11.3, 55.3 (2C), 55.7, 60.0, 96.2, 102.0 (2C), 112.6, 120.1, 126.0, 133.5, 134.3, 137.0, 137.4, 138.4, 147.4, 152.0, 152.4 (2C); ESI-MS: m/z = 478.1 [M+H]^+^.

#### 4-Bromo-3-methyl-5-(3-amino-4-methoxyphenyl)-1-(3,4,5-trimethoxyphenyl)-1*H*-pyrazole (7o)

The title compound was obtained according to the procedure described above for **11**. Yield: 92%; yellow solid; m.p. 128–129°C; ^1^H-NMR (400 MHz, CDCl_3_): δ 2.36 (3H, s), 3.67 (6H, s), 3.81 (3H, s), 3.85 (3H, s), 6.47 (2H, s), 6.66 (1H, d, *J* = 8.60 Hz), 6.71 (1H, s), 6.77 (1H, d, *J* = 8.60 Hz); ^13^C-NMR (100 MHz, CDCl_3_): δ 12.5, 55.5, 56.0 (2C), 60.9, 96.6, 102.1 (2C), 110.1, 115.9, 120.4, 121.9, 135.8, 136.2, 136.9, 141.1, 147.6, 148.0, 152.9 (2C); ESI-MS: m/z = 448.1 [M+H]^+^, 470.1 [M+Na]^+^.

#### 3-Ethyl-5-(4-methoxy-3-nitrophenyl)-1-(3,4,5-trimethoxyphenyl)-1*H*-pyrazole (7p)

The title compound was obtained according to the procedure described above for **7a**. Yield: 85%; yellow solid; m.p. 151–152°C; ^1^H-NMR (400 MHz, CDCl_3_): δ 1.34 (3H, t), 2.75 (2H, q), 3.73 (6H, s), 3.84 (3H, s), 3.96 (3H, s), 6.38 (1H, s), 6.50 (2H, s), 7.00 (1H, d, *J* = 8.85 Hz), 7.31 (1H, dd, *J* = 8.85 Hz, *J* = 2.21 Hz), 7.86(1H, d, *J* = 2.21Hz); ^13^C-NMR (100 MHz, CDCl_3_): δ 12.8, 20.4, 55.2 (2C), 55.7, 60.0, 102.4 (2C), 105.1, 112.4, 122.2, 124.5, 132.9, 134.3, 136.7, 138.4, 139.9, 151.4, 152.4 (2C), 154.5; ESI-MS: m/z = 414.1 [M+H]^+^, 827.3 [2M+H]^+^.

#### 3-Propyl-5-(4-methoxy-3-nitrophenyl)-1-(3,4,5-trimethoxyphenyl)-1*H*-pyrazole (7q)

The title compound was obtained according to the procedure described above for **7a**. Yield: 75%; yellow solid; m.p. 149–150°C; ^1^H-NMR (400 MHz, CDCl_3_): δ 1.03 (3H, t), 1.69–1.78 (2H, m), 2.69 (2H, t), 3.72 (6H, s), 3.84 (3H, s), 3.95 (3H, s), 6.36 (1H, s), 6.50 (2H, s), 7.00 (1H, d, *J* = 11.05 Hz), 7.31 (1H, dd, *J* = 11.05 Hz, *J* = 2.37 Hz), 7.84(1H, d, *J* = 2.37Hz); ^13^C-NMR (100 MHz, CDCl_3_): δ 13.0, 21.8, 29.2, 55.2 (2C), 55.7, 60.0, 102.4 (2C), 105.6, 112.4, 122.2, 124.5, 132.9, 134.3, 136.7, 138.4, 139.8, 151.4, 152.4 (2C), 153.2; ESI-MS: m/z = 428.1 [M+H]^+^.

#### 3-Ethyl-5-(3-amino-4-methoxyphenyl)-1-(3,4,5-trimethoxyphenyl)-1*H*-pyrazole (7r)

The title compound was obtained according to the procedure described above for **11**. Yield: 95%; pale yellow solid; m.p. 114–115°C; ^1^H-NMR (400 MHz, CDCl_3_): δ 1.32 (3H, t), 2.74 (2H, q), 3.70 (6H, s), 3.82 (3H, s), 3.84 (3H, s), 6.24 (1H, s), 6.54 (2H, s), 6.58 (1H, dd, *J* = 8.37 Hz, *J* = 2.05 Hz), 6.64 (1H, d, *J* = 2.05 Hz), 6.69 (1H, d, *J* = 8.37 Hz); ^13^C-NMR (100 MHz, CDCl_3_): δ 13.9, 21.5, 55.6, 56.1 (2C), 61.0, 102.8 (2C), 105.5, 110.1, 115.3, 119.4, 123.6, 135.5, 136.1, 136.8, 143.9, 147.4, 153.0 (2C), 15/5.1; ESI-MS: m/z = 384.2 [M+H]^+^, 767.4 [2M+H]^+^, 789.3 [2M+Na]^+^.

#### 3-Propyl-5-(3-amino-4-methoxyphenyl)-1-(3,4,5-trimethoxyphenyl)-1*H*-pyrazole (7s)

The title compound was obtained according to the procedure described above for **11**. Yield: 90%; pale yellow solid; m.p. 125–126°C; ^1^H-NMR (400 MHz, CDCl_3_): δ 1.03 (3H, t), 1.64–1.80(2H, m), 2.68 (2H, t), 3.69 (6H, s), 3.82 (3H, s), 3.83 (3H, s), 4.35 (2H, s), 6.23 (1H, s), 6.54 (2H, s), 6.58 (1H, dd, *J* = 8.30 Hz, *J* = 2.10 Hz), 6.64 (1H, d, *J* = 2.10 Hz), 6.69 (1H, d, *J* = 8.30 Hz); ^13^C-NMR (100 MHz, CDCl_3_): δ 14.1, 22.9, 30.4, 55.5, 56.1 (2C), 61.0, 102.8 (2C), 106.0, 110.1, 115.0, 119.1, 123.7, 136.0, 136.1, 136.8, 143.8, 147.2, 152.9 (2C), 153.8; ESI-MS: m/z = 398.1 [M+H]^+^.

#### 3-Methyl-1-phenyl-5-(3,4,5-trimethoxyphenyl)-1*H*-pyrazole (8a)

The title compound was obtained according to the procedure described above for **7a**. Yield: 74%; yellow solid; m.p. 88–89°C; ^1^H-NMR (400 MHz, CDCl_3_): δ 2.38 (3H, s), 3.63 (6H, s), 3.84 (3H, s), 6.32 (1H, s), 6.40 (2H, s), 7.27–7.52 (5H, m); ^13^C-NMR (100 MHz, CDCl_3_): δ 13.4, 55.9 (2C), 60.8, 105.8 (2C), 106.0, 107.2, 125.5 (2C), 127.5, 128.9 (2C), 138.0, 139.8, 143.8, 149.1, 153.0 (2C); ESI-MS: m/z = 325. 1 [M+H]^+^.

#### 3-Methyl-1-(4-methoxyphenyl)-5-(3,4,5-trimethoxy-phenyl)-1*H*-pyrazole (8b)

The title compound was obtained according to the procedure described above for **7a**. Yield: 78%; brown solid; m.p. 67–68°C; ^1^H-NMR (400 MHz, CDCl_3_): δ 2.41 (3H, s), 3.67 (6H, s), 3.80 (3H, s), 3.85 (3H, s), 6.31 (1H, s), 6.42 (2H, s), 6.88 (2H, d, *J* = 8.60 Hz), 7.24 (2H, d, *J* = 8.60 Hz); ^13^C-NMR (100 MHz, CDCl_3_): δ 13.5, 55.5, 55.9 (2C), 60.9, 105.9 (2C), 106.6, 114.0 (2C), 125.8, 126.9 (2C), 133.2, 137.9, 143.7, 148.8, 153.0 (2C), 158.8; ESI-MS: m/z = 355.1 [M+H]^+^, 731.3 [2M+Na]^+^.

#### 3-Methyl-1-(4-methylphenyl)-5-(3,4,5-trimethoxy-phenyl)-1*H*-pyrazole (8c)

The title compound was obtained according to the procedure described above for **7a**. Yield: 73%; yellow solid; m.p. 70–71°C; ^1^H-NMR (400 MHz, CDCl_3_): δ 2.33 (3H, s), 2.37 (3H, s), 3.64 (6H, s), 3.84 (3H, s), 6.29 (1H, s), 6.40 (2H, s), 7.14 (4H, m); ^13^C-NMR (100 MHz, CDCl_3_): δ 12.5, 20.0, 54.9 (2C), 59.9, 105.0 (2C), 105.9, 124.3 (2C), 124.9, 128.4 (2C), 136.2, 136.7, 136.9, 142.6, 148.0, 151.9 (2C); ESI-MS: m/z = 339.2 [M+H]^+^.

#### 3-Methyl-1-(2-methyl-5-nitrophenyl)-5-(3,4,5-trimethoxyphenyl)-1*H*-pyrazole (8d)

The title compound was obtained according to the procedure described above for **7a**. Yield: 71%; pale yellow solid; m.p. 102–103°C; ^1^H-NMR (400 MHz, CDCl_3_): δ 2.09 (3H, s), 2.39 (3H, s), 3.63 (6H, s), 3.81 (3H, s), 6.33 (2H, s), 6.39 (1H, s), 7.42 (1H, d, *J* = 8.44 Hz), 8.17 (1H, dd, *J* = 8.44 Hz, *J* = 2.36 Hz), 8.23 (1H, d, *J* = 2.36 Hz); ^13^C-NMR (100 MHz, CDCl_3_): δ 12.4, 17.1, 55.0 (2C), 59.9, 104.2 (2C), 105.3, 122.6, 122.7, 130.8, 137.5, 138.8, 138.9, 143.1, 144.5, 145.5, 149.0, 152.3 (2C); ESI-MS: m/z = 384.2 [M+H]^+^, 406.1 [M+Na]^+^, 422.1 [M+K]^+^, 789.3 [2M+Na]^+^.

#### 3-Methyl-1-(5-amino-2-methylphenyl)-5-(3,4,5-trimethoxyphenyl)-1*H*-pyrazole (8e)

The title compound was obtained according to the procedure described above for **11**. Yield: 90%; pale yellow solid; m.p. 69–70°C; ^1^H-NMR (400 MHz, CDCl_3_): δ 1.78 (3H, s), 2.37 (3H,s), 3.64 (6H, s), 3.82 (3H, s), 6.34 (1H, s), 6.43 (2H, s), 6.68 (2H, m), 6.98 (1H, d, *J* = 8.37 Hz); ^13^C-NMR (100 MHz, CDCl_3_): δ 13.5, 16.5, 55.9 (2C), 60.9, 104.8 (2C), 104.9, 115.3, 116.5, 125.6, 126.0, 131.5, 137.8, 140.3, 144.2, 148.9, 152.9 (2C); ESI-MS: m/z = 354.2 [M+H]^+^.

#### 3-Methyl-1-(4-methoxy-3-nitrophenyl)-5-(3,4,5-trimethoxyphenyl)-1*H*-pyrazole (8f)

The title compound was obtained according to the procedure described above for **7a**. Yield: 78%; pale yellow solid; m.p. 118–119°C; ^1^H-NMR (400 MHz, CDCl_3_): δ 2.37 (3H, s), 3.73 (6H, s), 3.87 (3H, s), 3.97 (3H, s), 6.31 (1H, s), 6.43 (2H, s),7.02 (1H, d, *J* = 8.79 Hz), 7.41 (1H, dd, *J* = 8.79 Hz, *J* = 2.61 Hz), 7.92 (1H, d, *J* = 2.61 Hz); ^13^C-NMR (100 MHz, CDCl_3_): δ 13.5, 56.2 (2C), 56.8, 60.9, 106.2 (2C), 108.0, 113.5, 122.0, 125.4, 130.1, 132.7, 138.5, 139.1, 143.8, 150.0, 151.4, 153.3 (2C).

#### 3-Methyl-1-(3-amino-4-methoxyphenyl)-5-(3,4,5-trimethoxyphenyl)-1*H*-pyrazole (8g)

The title compound was obtained according to the procedure described above for **11**. Yield: 89%; white solid; m.p. 56–57°C; ^1^H-NMR (400 MHz, CDCl_3_): δ 2.35 (3H, s), 3.68 (6H, s), 3.82 (3H, s), 3.84 (3H, s), 3.96 (2H, s), 6.27 (1H, s), 6.45 (2H, s), 6.54 (1H, dd, *J* = 8.52 Hz, *J* = 2.16 Hz), 6.68 (1H, d, *J* = 8.52 Hz), 6.80 (1H, d, *J* = 2.16 Hz); ^13^C-NMR (100 MHz, CDCl_3_): δ 13.4, 55.7, 55.9 (2C), 60.8, 105.8 (2C), 106.4, 109.9, 112.4, 115.7, 126.0, 133.6, 136.3, 137.8, 143.5, 146.6, 148.6, 152.9 (2C); ESI-MS: m/z = 370.1 [M+H]^+^, 739.3 [2M+H]^+^, 761.3 [2M+Na]^+^.

#### 3-Methyl-1-(3-allyloxy-4-methoxyphenyl)-5-(3,4,5-trimethoxyphenyl)-1*H*-pyrazole (8h)

The title compound was obtained according to the procedure described above for **7a**. Yield: 77%; brown solid; m.p. 98–99°C; ^1^H-NMR (400 MHz, CDCl_3_): δ 2.40 (3H, s), 3.68 (6H, s), 3.85 (3H, s), 3.87(3H, s), 4.50 (2H, d, *J* = 5.07 Hz), 5.18–5.21 (1H, m), 5.27–5.32 (1H, m), 5.90–6.00 (1H, m), 6.31 (1H, s), 6.42 (2H, s), 6.81–6.87 (3H, m); ^13^C-NMR (100 MHz, CDCl_3_): δ 13.5, 56.0 (2C), 56.2, 60.9, 69.9, 106.0 (2C), 106.8, 111.2, 111.3, 118.1 (2C), 126.0, 132.6, 133.2, 137.9, 143.6, 147.9, 148.7, 148.9, 153.0 (2C).

#### 3-Methyl-1-(3-hydroxy-4-methoxyphenyl)-5-(3,4,5-trimethoxyphenyl)-1*H*-pyrazole (8i)

The title compound was obtained according to the procedure described above for **7i**. Yield: 90%; grey solid; m.p. 167–168°C; ^1^H-NMR (400 MHz, CDCl_3_): δ 2.36 (3H, s), 3.66 (6H, s), 3.80 (3H, s), 3.84 (3H, s), 6.29 (1H, s), 6.44 (2H, s), 6.67 (1H, dd, *J* = 8.59 Hz, *J* = 2.26 Hz), 6.72 (1H, d, *J* = 8.59 Hz), 6.99 (1H, d, *J* = 2.26 Hz); ^13^C-NMR (100 MHz, CDCl_3_): δ 13.4, 56.0 (3C), 60.9, 105.9 (2C), 106.5, 110.5, 113.1, 117.4, 125.8, 133.4, 137.9, 143.8, 146.1, 146.6, 148.8, 152.9 (2C); ESI-MS: m/z = 371.2 [M+H]^+^, 741.3 [2M+H]^+^, 763.3 [2M+Na]^+^.

### Biology

#### Cell line and culture conditions

Pulmonary adenocarcinoma cell lines (A549) and gastric adenocarcinoma cell lines (SGC-7901) were provided as gifts (purchased from the Cell Resource Center of Shanghai Institutes for Biological Sciences) from the China-Japan Research Institute of Medical and Pharmaceutical Sciences, Shenyang Pharmaceutical University. Fibrosarcoma cell lines (HT-1080) were purchased from the Cell Resource Center of Shanghai Institutes for Biological Sciences. All cells were maintained in DMEM containing 1% antibiotics (100 units of penicillin and 10 mg streptomycin per mL in 0.9% normal saline) and 10% foetal bovine serum in a humidified atmosphere containing 5% CO_2_ at 37°C.

#### MTT assay

Antiproliferative activities of CA-4 and the target compounds were measured using a colourimetric assay using 3-(4,5-dimethylthiazol-2yl-)-2,5-diphenyltetrazolium bromide (MTT). Approximately 3 × 10^4^ cells were seeded in a 96-well plate. After 24 h of incubation at 37°C, cells were exposed to compounds of differing concentrations (0.0008 μg/mL, 0.004 μg/mL, 0.02 μg/mL, 0.10 μg/mL, 0.50 μg/mL, 2.5 μg/mL, 12.5 μg/mL, 62.5 μg/mL) for 72 h. After treatment, cells were washed with 1X PBS followed by addition of 100 μL of 0.05% MTT reagent to each well, followed by incubation for 4 h at 37°C. After incubation, the supernatant from each well was carefully removed and the formazan crystals were dissolved in 100 μL of DMSO. The colour density was measured spectrophotometrically at 490 nm using a microplate reader (SpectraMax Plus384, Molecular Devices Corp., USA). The percentage of cell growth inhibition was calculated as follow: inhibition ratio % = (A_control_—A_treated_) / A_control_ × 100%. The 50% inhibitory concentration (IC_50_) was defined as the concentration that reduced the absorbance of the untreated wells by 50% of the vehicle in the MTT assay.

#### Tubulin assembly assay

The effect of compounds **7i** and **7k** on the polymerization of purified brain tubulin was determined employing a fluorescence-based tubulin polymerization assay kit (Cat. #BK011P, Cytoskeleton, Inc., USA) according to the manufacturer’s protocol. Tubulin was re-suspended in ice-cold G-PEM buffer (80 mM PIPES, 2 mM MgCl_2_, 0.5 mM EGTA, 1 mM GTP, 20% (v/v) glycerol) and added to wells on a 96-well plate containing the designated concentration of drugs or vehicle. Samples were mixed well, and tubulin assembly was monitored (emission wavelength: 450 nm; excitation wavelength: 360 nm) at 1 min intervals for 90 min at 37°C using a plate reader (FASCalibur, BD Biosciences, USA). IC_50_ values were calculated at 80 min using SPSS software in three experiments.

#### Immunostaining of tubulin assembly and DAPI nuclear staining

Immunostaining was performed to detect microtubule-associated tubulin protein after exposure to CA-4 and compound **7k**. The SGC-7901 and HT-1080 cells were seeded at a density of 1 × 10^4^ per well on a 24-well plate and grown for 24 h. Cells were treated with vehicle or 2-fold IC_50_ concentrations of CA-4 or **7k** for 48 h. The control and treated cells were fixed in acetone:methanol (1:1) for 30 min at -20°C, washed with 1X PBS, incubated for 30 min in 1X PBS containing 2% BSA, and then 0.1% Triton X-100. The primary tubulin antibody (Cell Signaling Technology, MA, USA) was diluted (1:100) with 2% BSA in 1X PBS and incubated overnight at 4°C. The cells were washed with 1X PBS to remove unbound primary antibody, and then cells were incubated for 3 h at 37°C with FITC-conjugated anti-mouse secondary antibody diluted 1:1000 with 2% BSA in PBS. The cells were washed with 1X PBS to remove unbound secondary antibody, the nucleus was stained with 4,6-diamino-2-phenolindole dihydrochloride (DAPI), and immunofluorescence was detected using an Olympus inverted fluorescence microscope.

### Docking study

The initial coordinates for tubulin were taken from the crystal structure of tubulin in complex with colchicine (PDB ID: 1SA0) obtained from the Protein Data Bank. Molecular docking was performed using the Discovery Studio 3.0 software package’s CDOCKER protocol with the default settings. The protein was prepared by removing all of the ions and substructures present and then adding hydrogen atoms. In the docking process, the active site was defined along with the CA-4 (**1**) complex. Compound **7k** was docked into the active site and the docking simulations were performed using the Discovery studio 3.0 programme.

## Supporting Information

S1 File Contents: ^1^H and ^13^C NMR spectra of all target compounds and representative intermediates. Supporting Information include the NMR (^1^H and ^13^C) spectra of the synthesized pyrazole derivatives and representative intermediates.
(PDF)

## References

[1] Jordan MA, Wilson L (2004) Microtubules as a target for anticancer drugs. Nat Rev Cancer 4: 253–265. 15057285 10.1038/nrc1317

[2] Dumontet C, Jordan MA (2010) Microtubule-binding agents: a dynamic field of cancer therapeutics. Nat Rev Drug Discov 9: 790–803. 10.1038/nrd3253 20885410 PMC3194401

[3] Amos LA (2004) Microtubule structure and its stabilization. Org Biomol Chem 2: 2153–2160. 15280946 10.1039/b403634d

[4] Lu Y, Chen JJ, Xiao M, Li W, Miller DD (2012) An overview of tubulin inhibitors that interact with the colchicine binding site. Pharm Res 29: 2943–2971. 10.1007/s11095-012-0828-z 22814904 PMC3667160

[5] Kanthou C, Tozer GM (2009) Microtubule depolymerizing vascular disrupting agents: Novel therapeutic agents for oncology and other pathologies. Int J Exp Pathol 90: 284–294. 10.1111/j.1365-2613.2009.00651.x 19563611 PMC2697551

[6] Tron GC, Pirali T, Sorba G, Pagliai F, Busacca S, Genazzani AA (2006) Medicinal chemistry of combretastatin A4: present and future directions. J Med Chem 49: 3033–3044. 16722619 10.1021/jm0512903

[7] Pettit GR, Singh SB, Hamel E, Lin CM, Alberts DS, Garcia-Kendall D (1989) Isolation and structure of the strong cellgrowth and tubulin inhibitor combretastatin A-4. Experientia 45: 209–211. 2920809 10.1007/BF01954881

[8] McGown AT, Fox BW (1990) Differential cytotoxicity of Combretastatins A1 and A4 in two daunorubicin-resistant P388 cell lines. Cancer Chem Pharm 26: 79–81. 10.1007/BF02940301 2322992

[9] Pettit GR, Toki BE, Herald DL, Boyd MR, Hamel E, Pettit RK, et al (1999) Antineoplastic agents. 410. Asymmetric hydroxylation of trans-combretastatin A-4. J Med Chem 42: 1459–1465. 10212132 10.1021/jm9807149

[10] Lee L, Davis R, Vanderham J, Hills P, Mackay H, Brown T, et al (2008) 1,2,3,4-Tetrahydro-2-thioxopyrimidine analogs of combretastatin-A4. Eur J Med Chem 43: 2011–2015. 10.1016/j.ejmech.2007.11.030 18226429

[11] Blanch NM, Chabot GG, Quentin L, Scherman D, Bourg S, Dauzonne D (2012) In vitro and in vivo biological evaluation of new 4,5-disubstituted 1,2,3-triazoles as cis-constrained analogs of combretastatin A4. Eur J Med Chem 54: 22–32. 10.1016/j.ejmech.2012.04.017 22647220

[12] Tron GC, Pagliai F, Del GE, Genazzani AA, Sorba G (2005) Synthesis and cytotoxic evaluation of combretafurazans. J Med Chem 48: 3260–3268. 15857132 10.1021/jm049096o

[13] Biersack B, Effenberger K, Knauer S, Ocker M, Schobert R (2010) Ru(h6-arene) complexes of combretastatin-analogous oxazoles with enhanced anti-tumoral impact. Eur J Med Chem 45: 4890–4896. 10.1016/j.ejmech.2010.07.061 20727621

[14] Kaffy J, Pontikis R, Carrez D, Croisy A, Monneret C, Florent JC (2006) Isoxazole-type derivatives related to combretastatin A-4, synthesis and biological evaluation. Bioorg Med Chem 14: 4067–4077. 16510288 10.1016/j.bmc.2006.02.001

[15] Wang L, Woods KW, Li Q, Barr KJ, McCroskey RW, Hannick SM, et al (2002) Potent, orally active heterocycle-based combretastatin A-4 analogues: synthesis, structure-activity relationship, pharmacokinetics, and in vivo antitumor activity evaluation. J Med Chem 45: 1697–1711. 11931625 10.1021/jm010523x

[16] Zhang Q, Peng Y, Wang XI, Keenan SM, Arora S, Welsh WJ (2007) Highly potent triazole-based tubulin polymerization inhibitors. J Med Chem 50: 749–754. 17249649 10.1021/jm061142s PMC2694353

[17] Kumara V, Kaur K, Gupta GK, Sharmac AK (2013) Pyrazole containing natural products: Synthetic preview and biological significance. Eur J Med Chem 69: 735–753. 10.1016/j.ejmech.2013.08.053 24099993

[18] Liu JJ, Zhao M, Zhang X, Zhao X, Zhu HL (2013) Pyrazole Derivatives as Antitumor, Anti-Inflammatory and Antibacterial Agents. Mini-Reviews in Med Chem 10: 1957–1966. 10.2174/13895575113139990078 23937232

[19] Li M; Zhao BX (2014) Progress of the synthesis of condensed pyrazole derivatives (from 2010 to mid-2013). Eur J Med Chem 85: 311–340. 10.1016/j.ejmech.2014.07.102 25104650

[20] Tsyganov DV, Konyushkin LD, Karmanova IB, Firgang SI, Strelenko YA, Semenova MN, et al (2013) cis-Restricted 3-aminopyrazole analogues of combretastatins: synthesis from plant polyalkoxybenzenes and biological evaluation in the cytotoxicity and phenotypic sea urchin embryo assays. J Nat Prod 76: 1485–1491. 10.1021/np400310m 23924236

[21] Burja B, Cimbora-Zovko T, Tomic S, Jelušic T, Kocevar M, Polanc S, et al (2010) Pyrazolone-fused combretastatins and their precursors: synthesis, cytotoxicity, antitubulin activity and molecular modeling studies. Bioorg Med Chem 18: 2375–2387. 10.1016/j.bmc.2010.03.006 20338766

[22] Gong JX, Huang K, Wang F, Yang L, Feng Y, Li H, et al (2009) Preparation of two sets of 5,6,7-trioxygenated dihydroflavonol derivatives as free radical scavengers and neuronal cell protectors to oxidative damage. Bioorg Med Chem 17: 3414–3425. 10.1016/j.bmc.2009.03.032 19362850

[23] Clive DLJ, Angoh G, Bennett SM (1987) Radical spirocyclization: synthesis of an appropriately oxygenated spiro compound related to the antitumor antibiotic Fredericamycin A. J Org Chem 52: 1339–1342.

[24] Ferlin MG, Marzano C, Via LD, Chilin A, Zagotto G, Guiotto A, et al (2005) New water soluble pyrroloquinoline derivatives as new potential anticancer agents. Bioorg Med Chem Lett 13: 4733–4739. 15936202 10.1016/j.bmc.2005.04.080

[25] Fehnel EA (1958) Quinoline analogs of podophyllotoxin. I. preliminary experiments. syntheses of some 4-phenylquinoline derivatives. J Org Chem 23: 432–434.

[26] Genin MJ, Biles C, Keiser BJ, Poppe SM, Swaney SM, Tarpley WG, et al (2000) Novel 1,5-diphenylpyrazole nonnucleoside HIV-1 reverse transcriptase inhibitors with enhanced activity versus the delavirdine-resistant P236L mutant: lead identification and SAR of 3- and 4-substituted derivatives. J Med Chem 43: 1034–1040. 10715167 10.1021/jm990383f

[27] Kadam SM, Nayak SK, Banerji A (1992) Low-valent titanium: a new approach to deprotection of allyl and benzyl groups. Tetrahedron Lett 33: 5129–5132.

[28] Frey RR, Curtin ML, Albert DH, Glaser KB, Pease LJ, Soni NB, et al (2008) 7-Aminopyrazolo[1,5- a] pyrimidines as potent multitargeted receptor tyrosine kinase inhibitors. J Med Chem 51: 3777–3787. 10.1021/jm701397k 18557606

[29] Zhao ZG, Wang ZX (2007) Halogenation of pyrazoles using N-Halosuccinimides in CCl4 and in water. Synth Commun 37: 137–147.

[30] Serrette AG, Lai CK, Swager TM (1994) Complementary shapes in columnar liquid crystals: structural control in homo- and heteronuclear bimetallic assemblies. Chem Mater 6: 2252–2268.

