# Supplementary material for: Synthesis and Biological Evaluation of 3-Alkyl-1,5-Diaryl-1H-Pyrazoles as Rigid Analogues of Combretastatin A-4 with Potent Antiproliferative Activity
Source: PLoS One. 2015 Jun 10;10(6):e0128710. doi: 10.1371/journal.pone.0128710 (PMC4462585; doi:10.1371/journal.pone.0128710)

Contents: ^{1}H and ^{8}C NMR spectra of all target compounds and representative intermediates.

Target compounds:

3-Methyl-5-phenyl-1-(3,4,5-trimethoxyphenyl)-1*H*-pyrazole(7a)

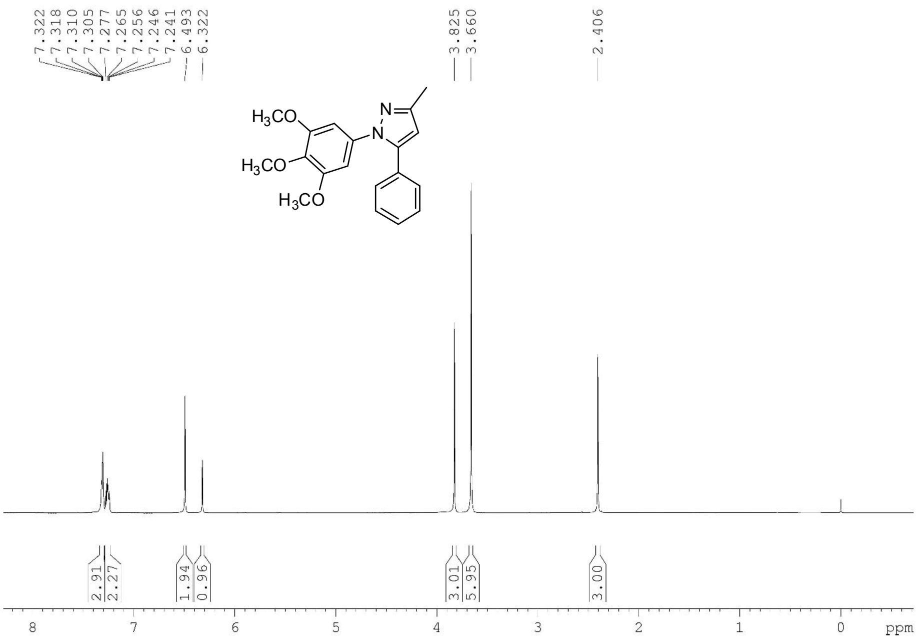

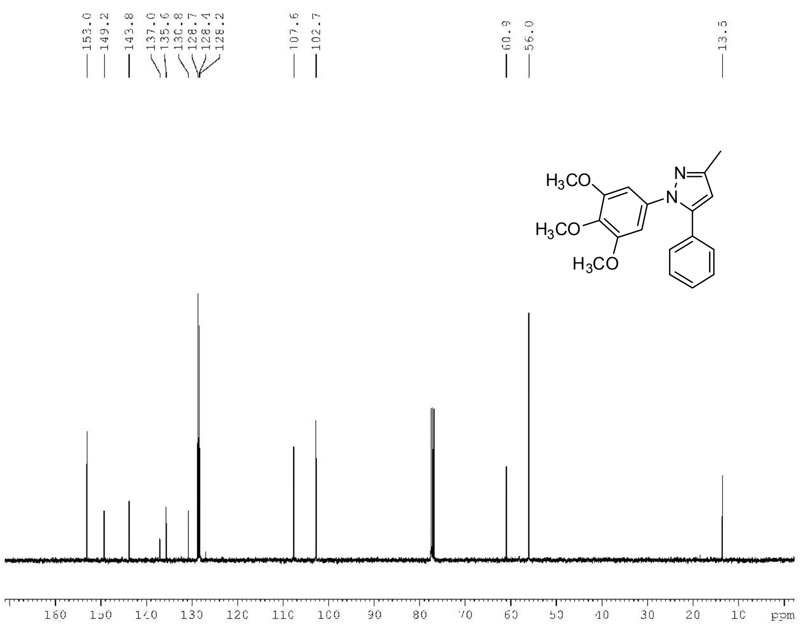

3-Methyl-5-(4-methoxyphenyl)-1-(3,4,5-trimethoxyphenyl)-1*H*-pyrazole(7b)

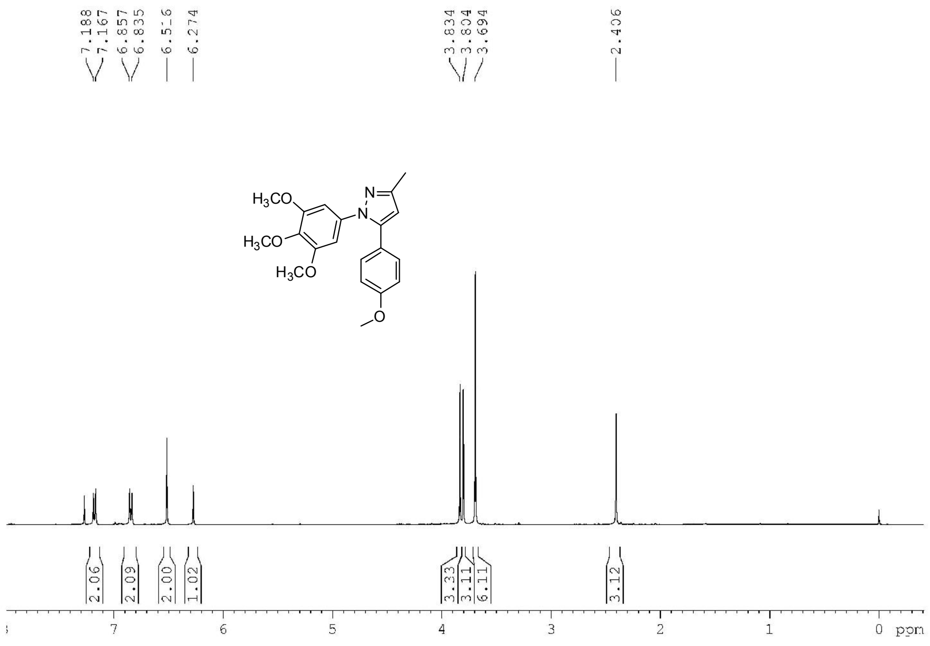

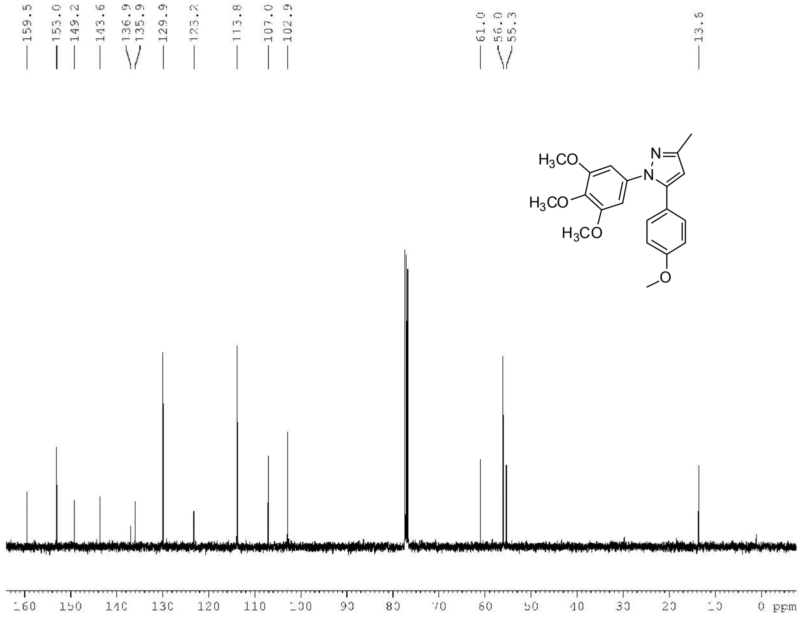

3-Methyl-5-(4-methylphenyl)-1-(3,4,5-trimethoxyphenyl)-1*H*-pyrazole(7c)

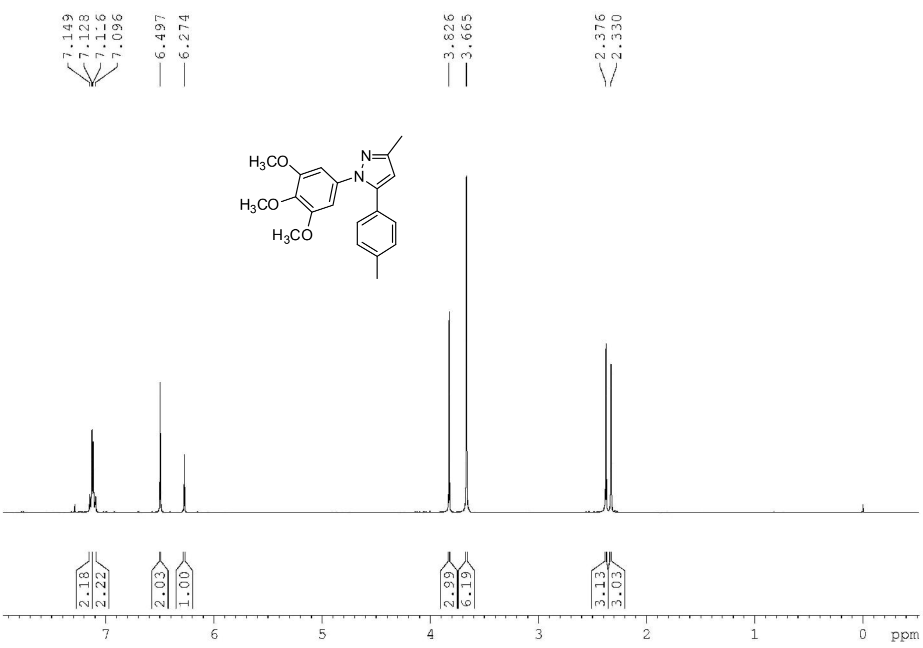

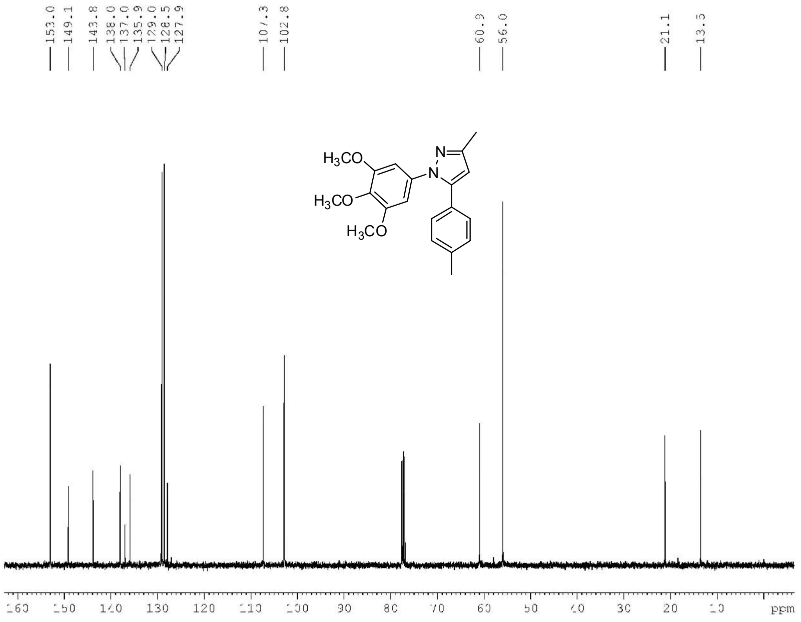

3-Methyl-5-(4-fluorophenyl)-1-(3,4,5-trimethoxyphenyl)-1*H*-pyrazole (7d)

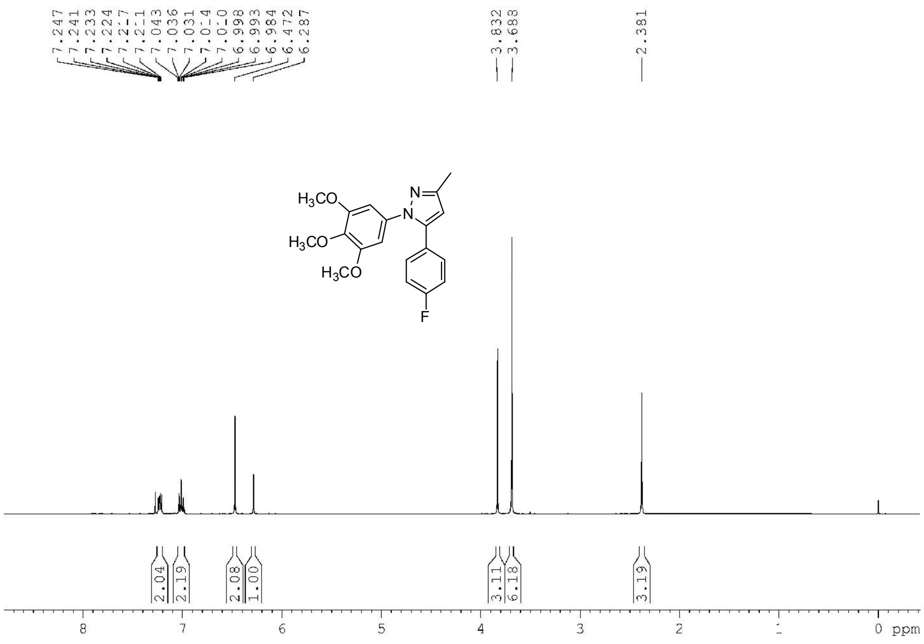

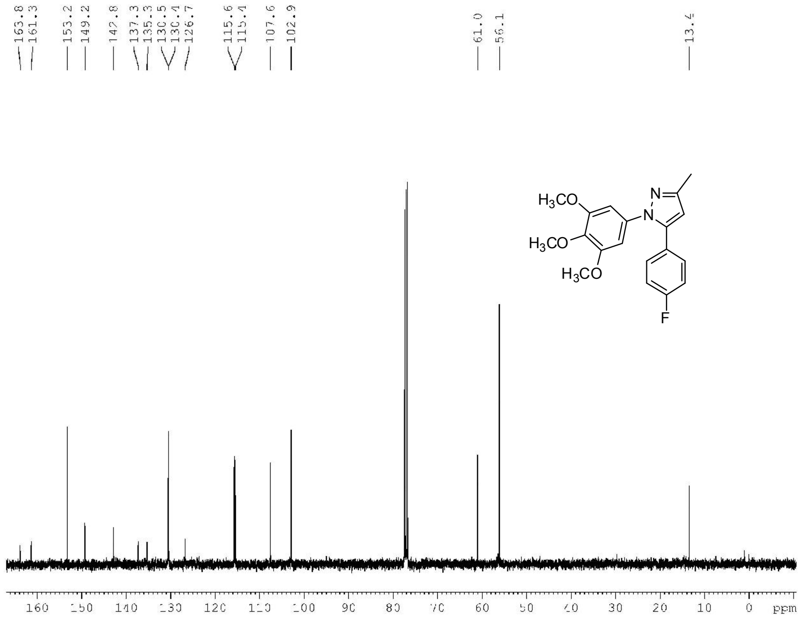

3-Methyl-5-(3-fluoro-4-methoxyphenyl)-1-(3,4,5-trimethoxyphenyl)-1*H*-pyrazole (7e)

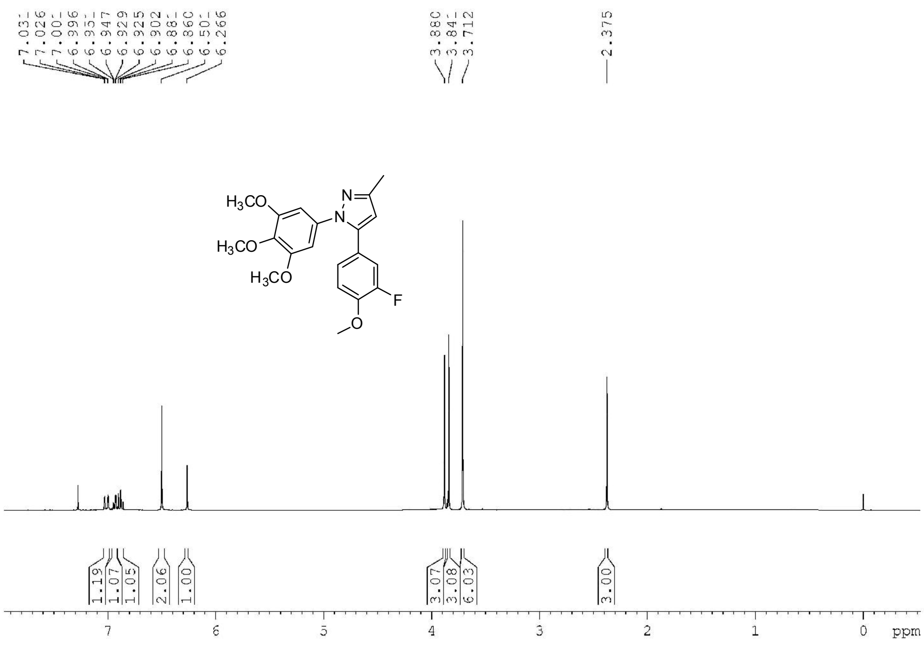

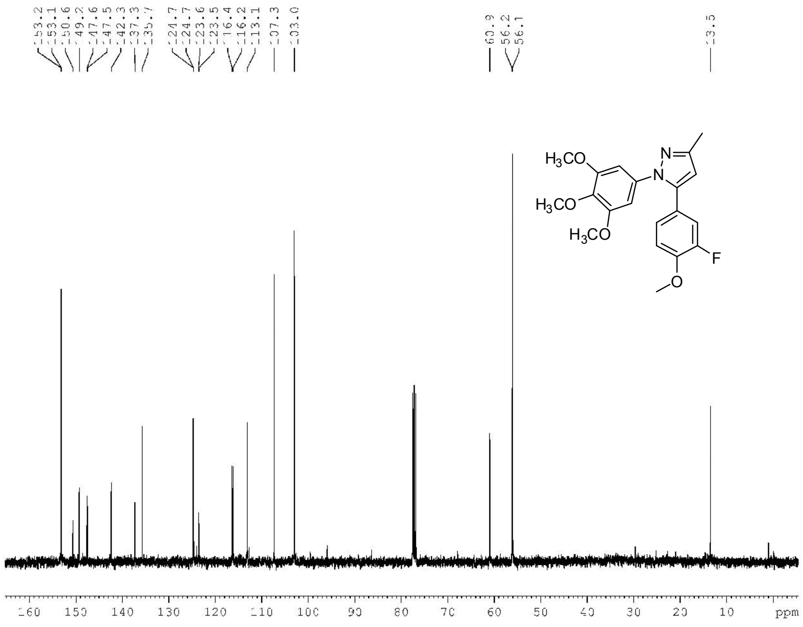

3-Methyl-5-(4-allyloxy-3-methoxyphenyl)-1-(3,4,5-trimethoxyphenyl)-1*H*-pyrazole (7f)

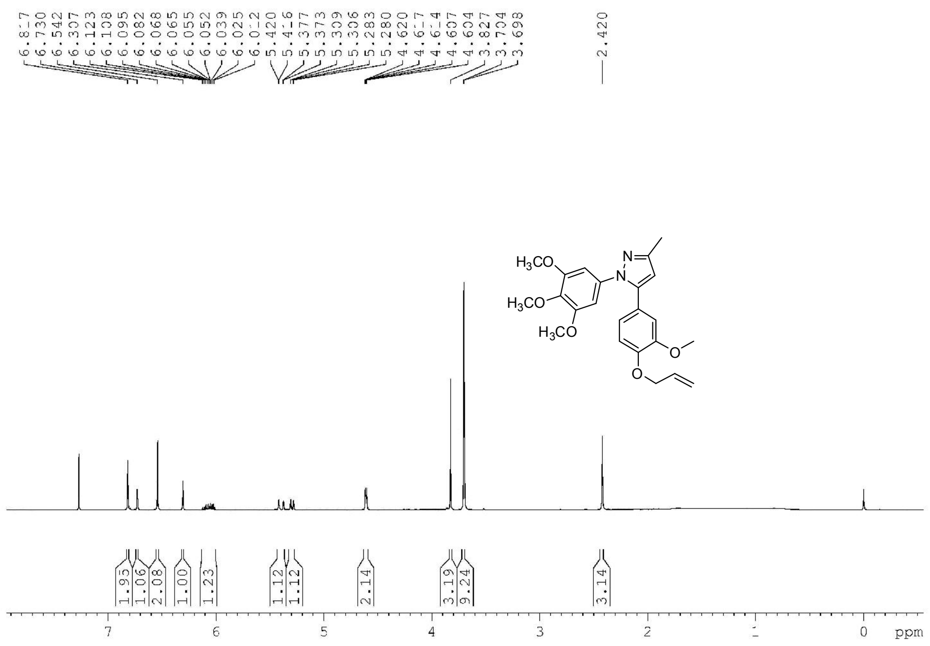

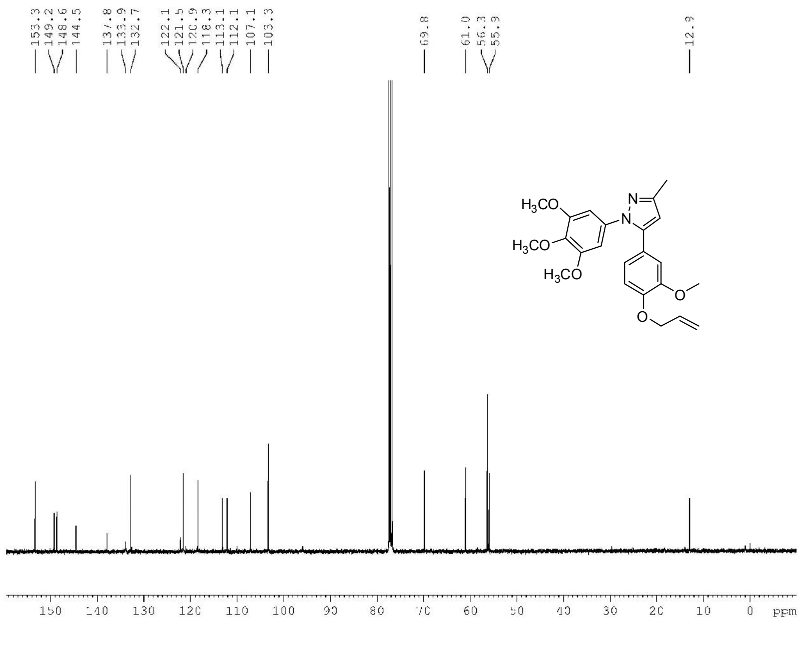

3-Methyl-5-(3-allyloxy-4-methoxyphenyl)-1-(3,4,5-trimethoxyphenyl)-1*H*-pyrazole (7g)

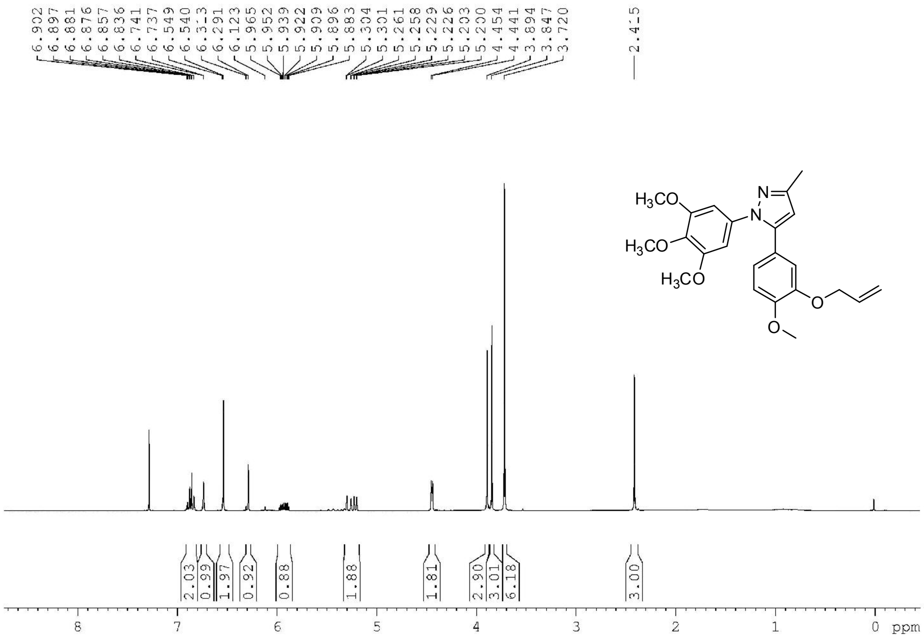

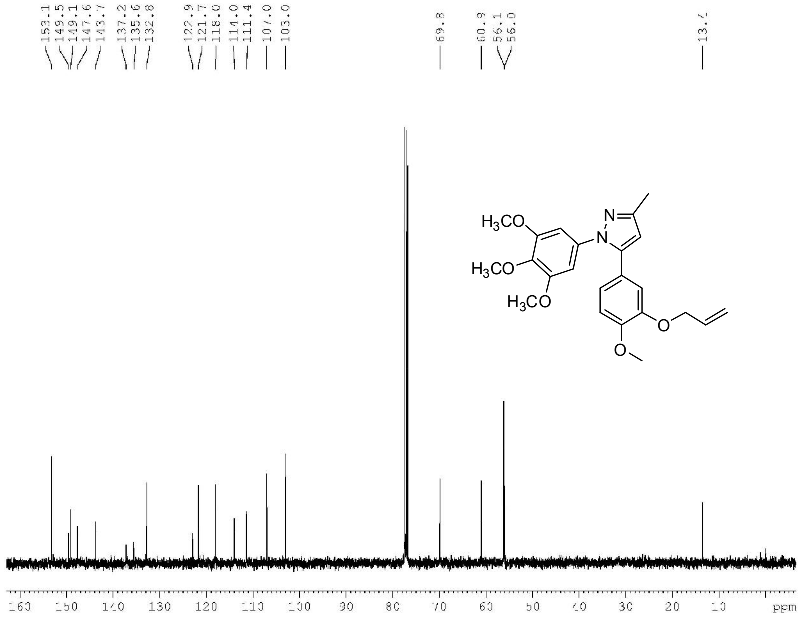

3-Methyl-5-(4-methoxy-3-nitrophenyl)-1-(3,4,5-trimethoxyphenyl)-1*H*-pyrazole (7h)

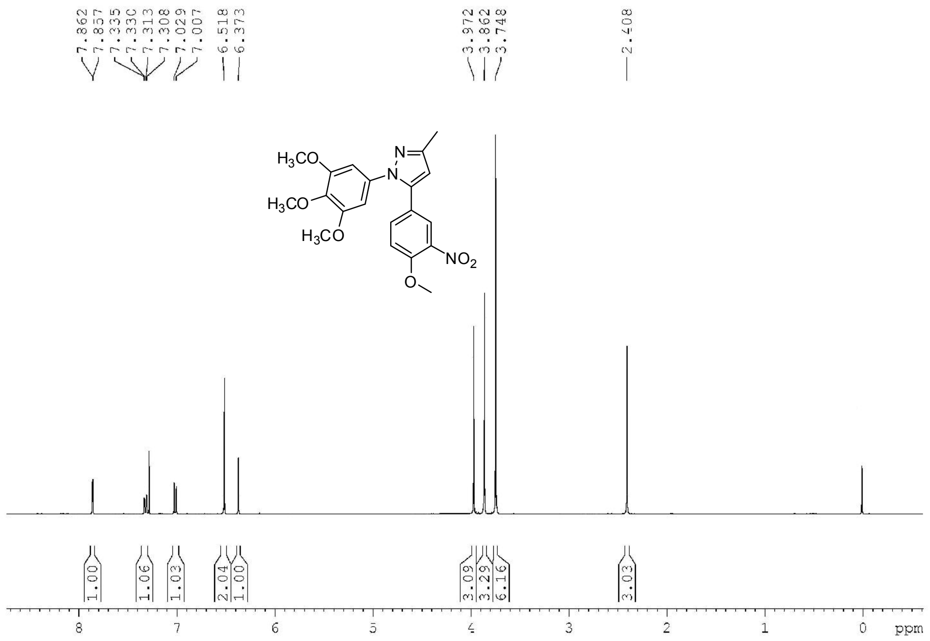

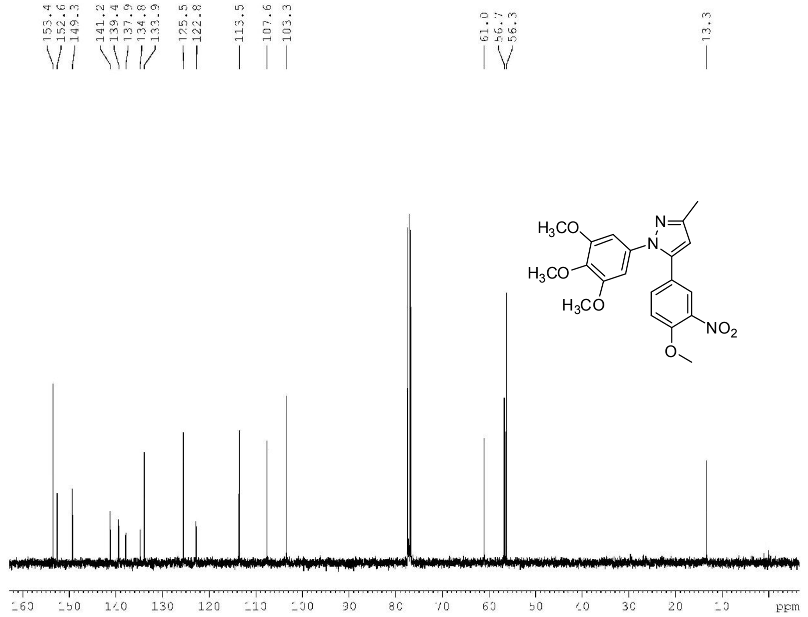

3-Methyl-5-(3-hydroxy-4-methoxyphenyl)-1-(3,4,5-trimethoxyphenyl)-1*H*-pyrazole (7i)

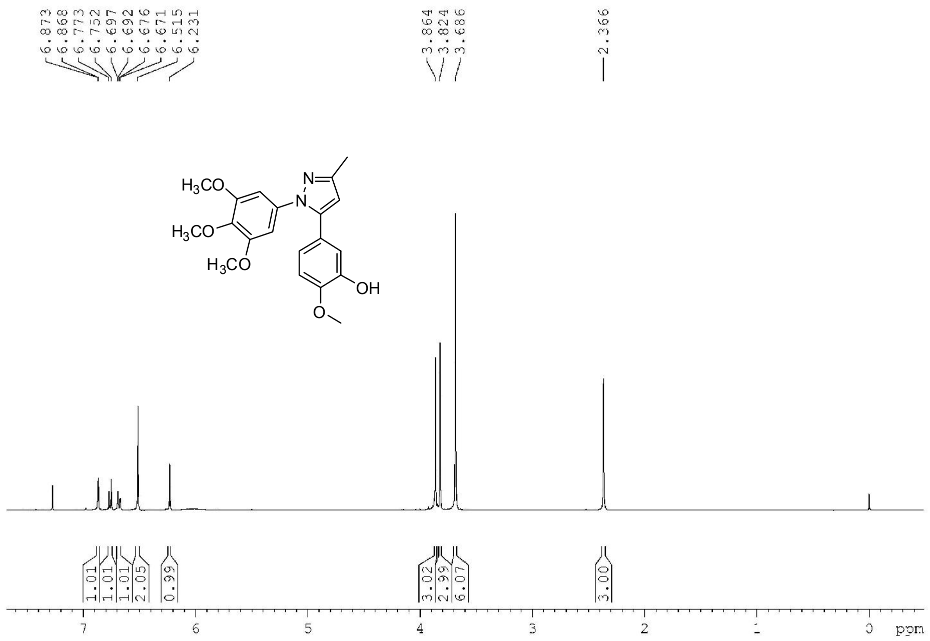

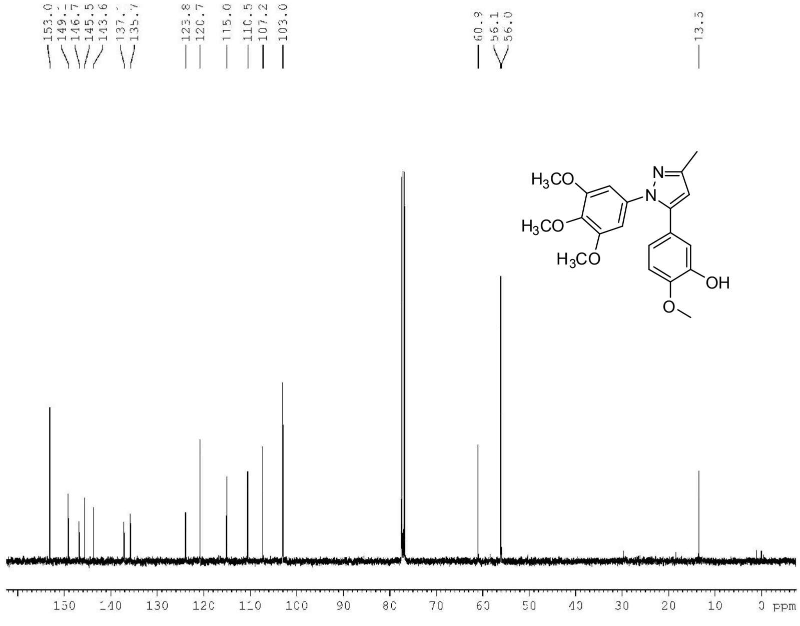

3-Methyl-5-(4-hydroxy-3-methoxyphenyl)-1-(3,4,5-trimethoxyphenyl)-1*H*-pyrazole (7j)

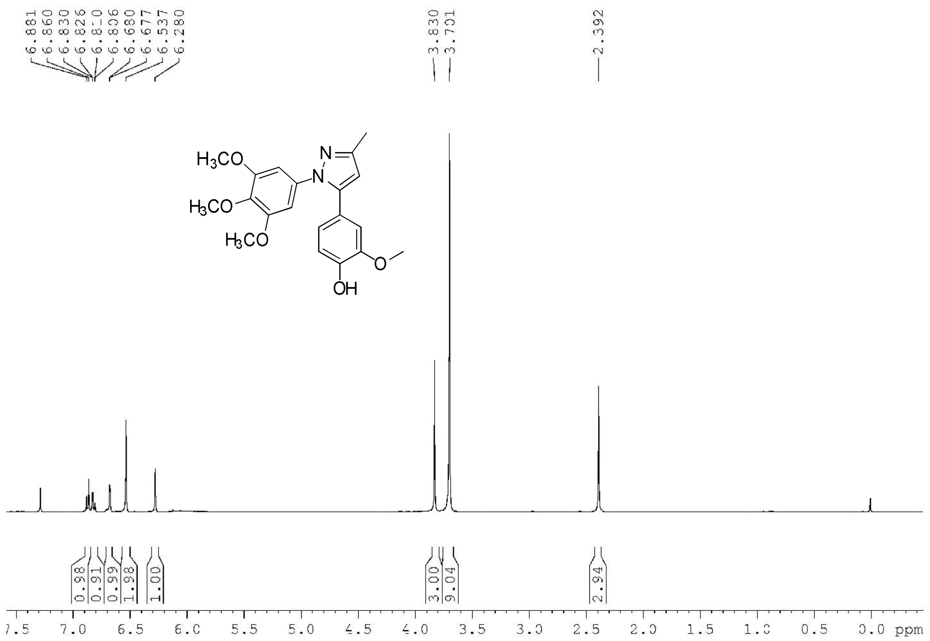

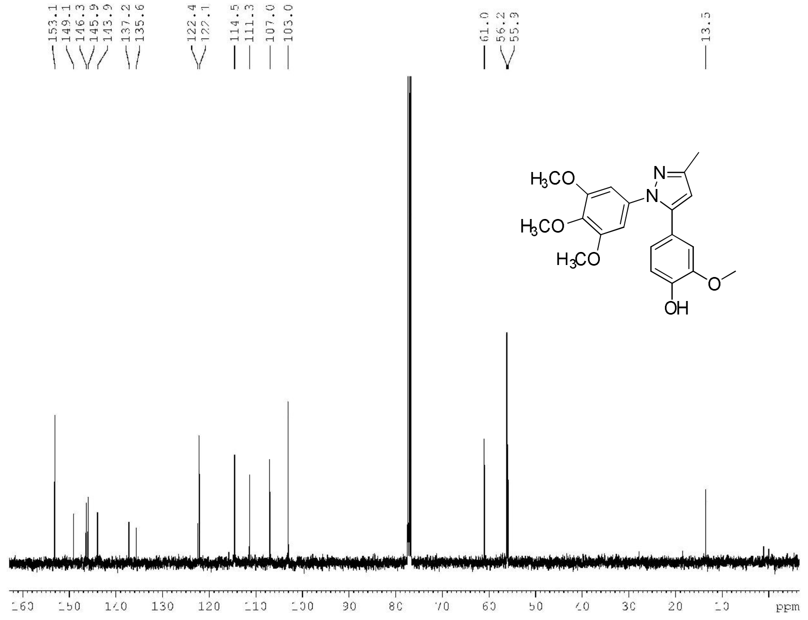

3-Methyl-5-(3-amino-4-methoxyphenyl)-1-(3,4,5-trimethoxy-phenyl)-1*H*-pyrazole (7k)

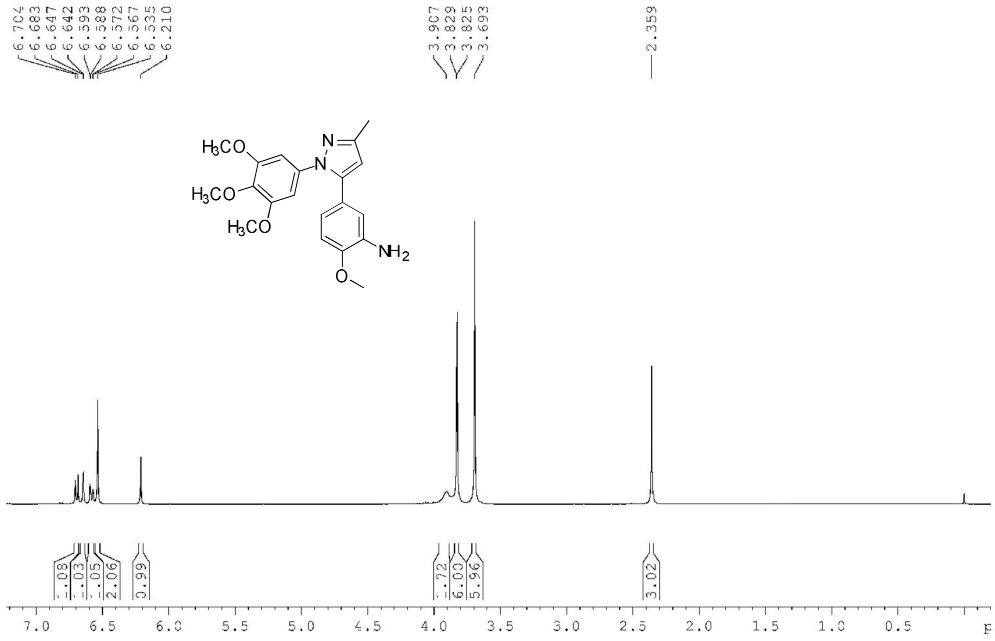

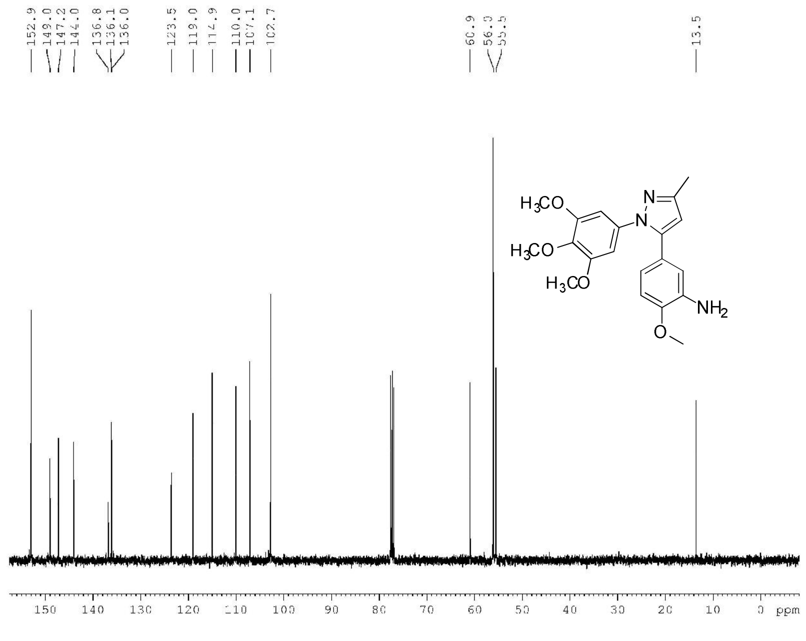

4-Formyl-3-methyl-5-(3-hydroxy-4-methoxyphenyl)-1-(3,4,5-trimethoxyphenyl)-1*H*-pyrazole (7l)

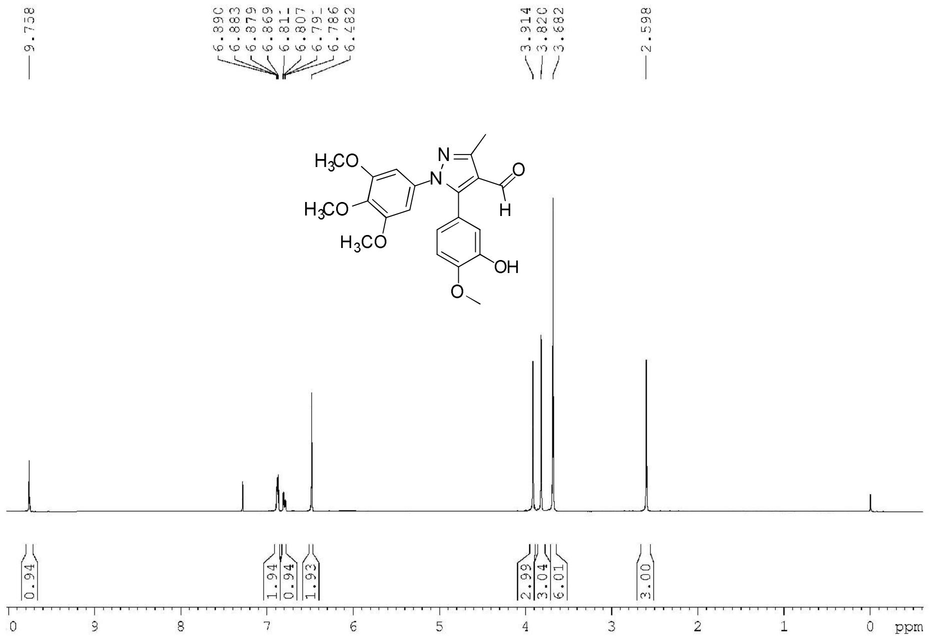

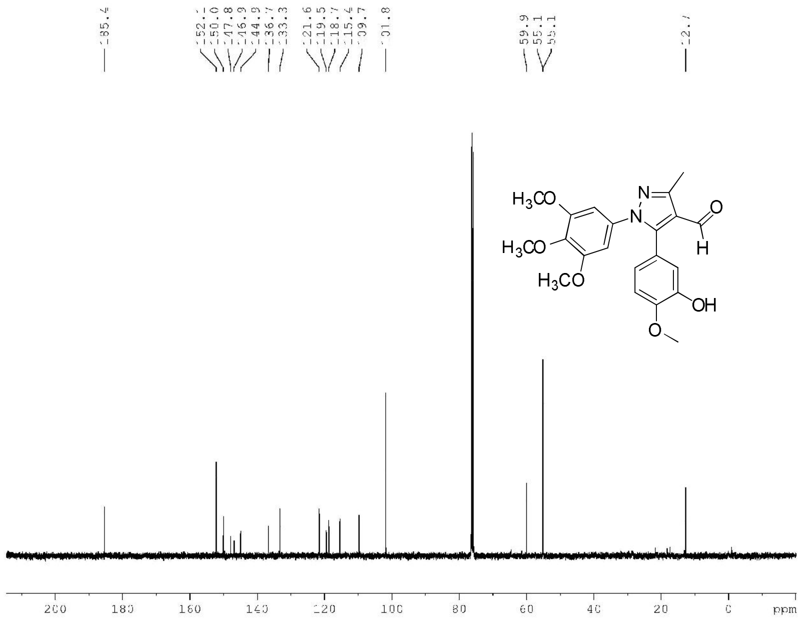

4-Hydroxymethyl-3-methyl-5-(3-hydroxy-4-methoxyphenyl)-1-(3,4,5-trimethoxyphenyl)-1*H*-pyrazole (7m)

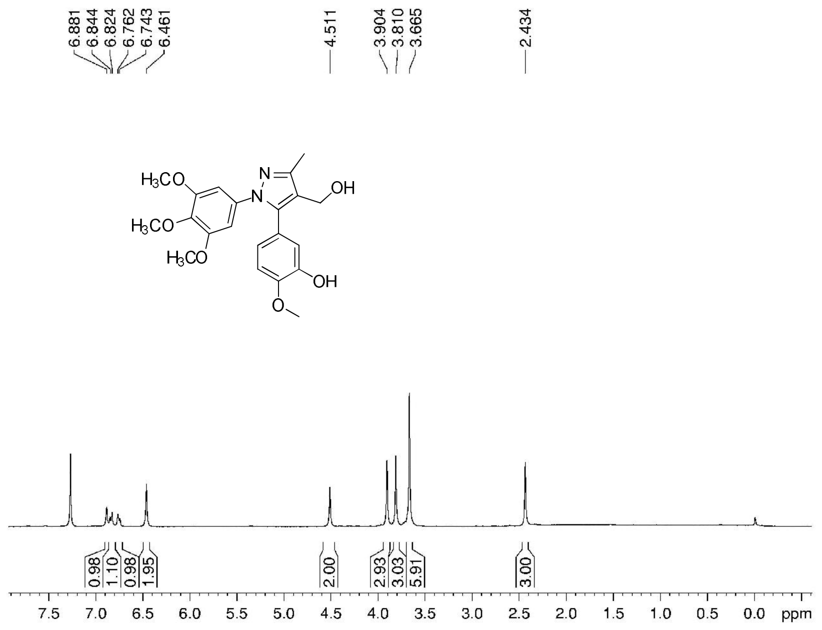

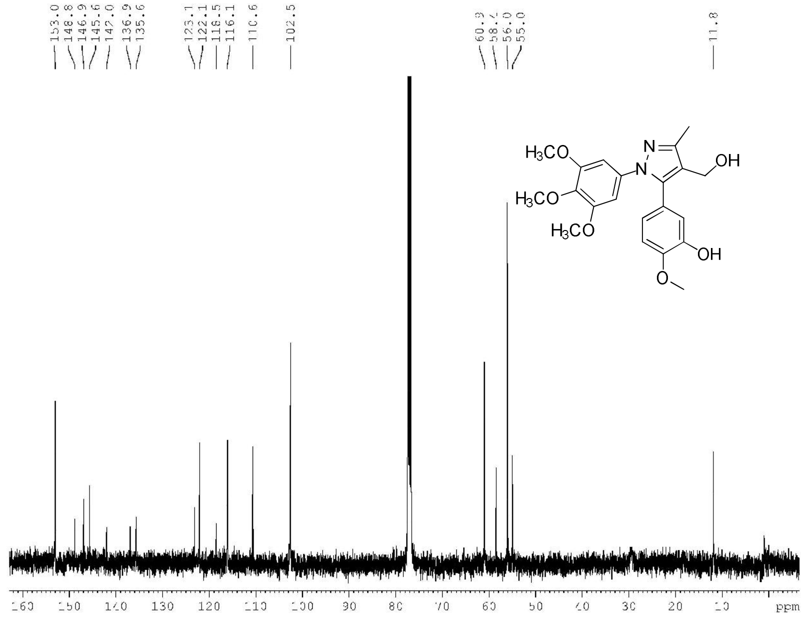

4-Bromo-3-methyl-5-(4-methoxy-3-nitrophenyl)-1-(3,4,5-trimethoxyphenyl)-1*H*-pyrazole (7n)

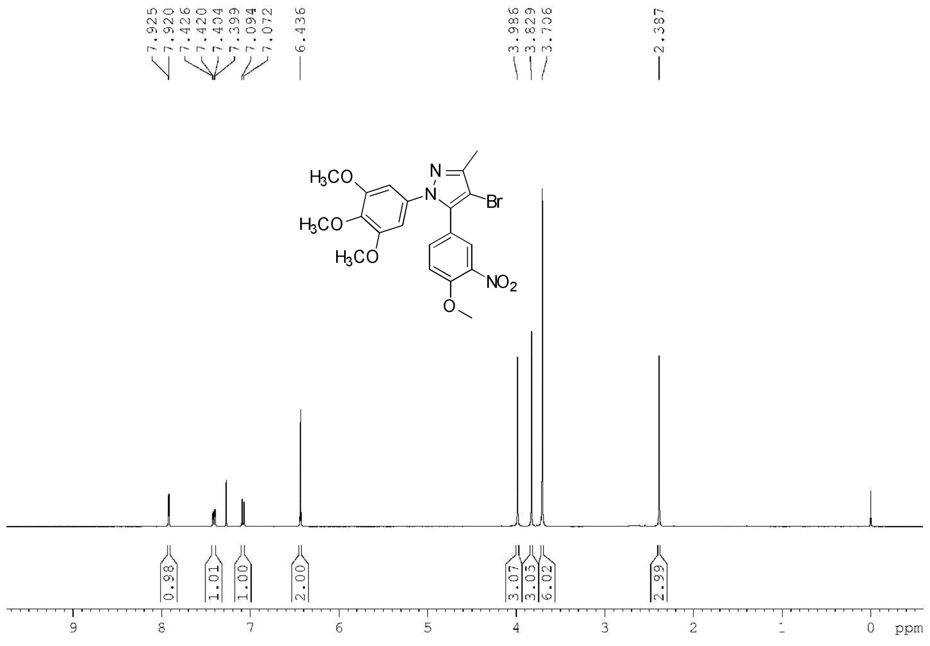

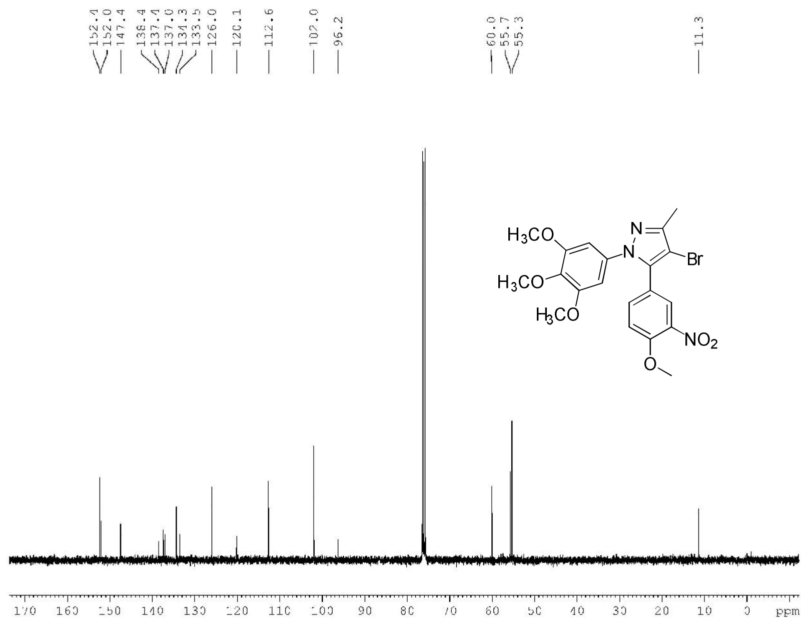

4-Bromo-3-methyl-5-(3-amino-4-methoxyphenyl)-1-(3,4,5-trimethoxyphenyl)-1*H*-pyrazole (7o)

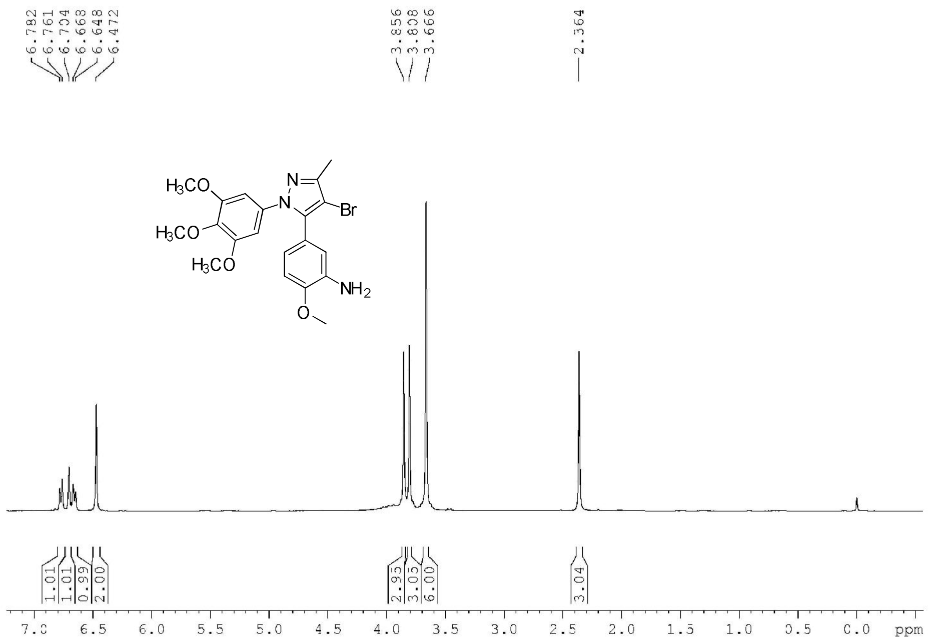

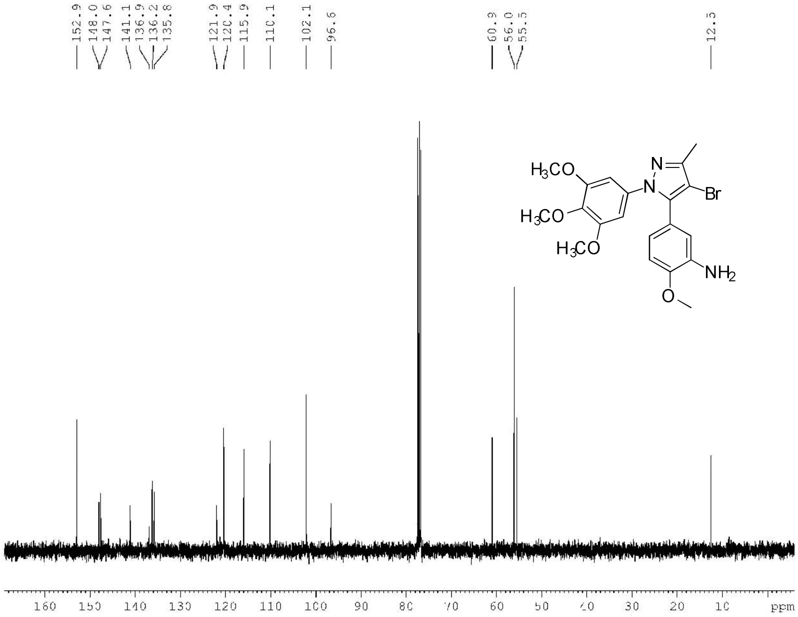

3-Ethyl-5-(4-methoxy-3-nitrophenyl)-1-(3,4,5-trimethoxyphenyl)-1*H*-pyrazole (7p)

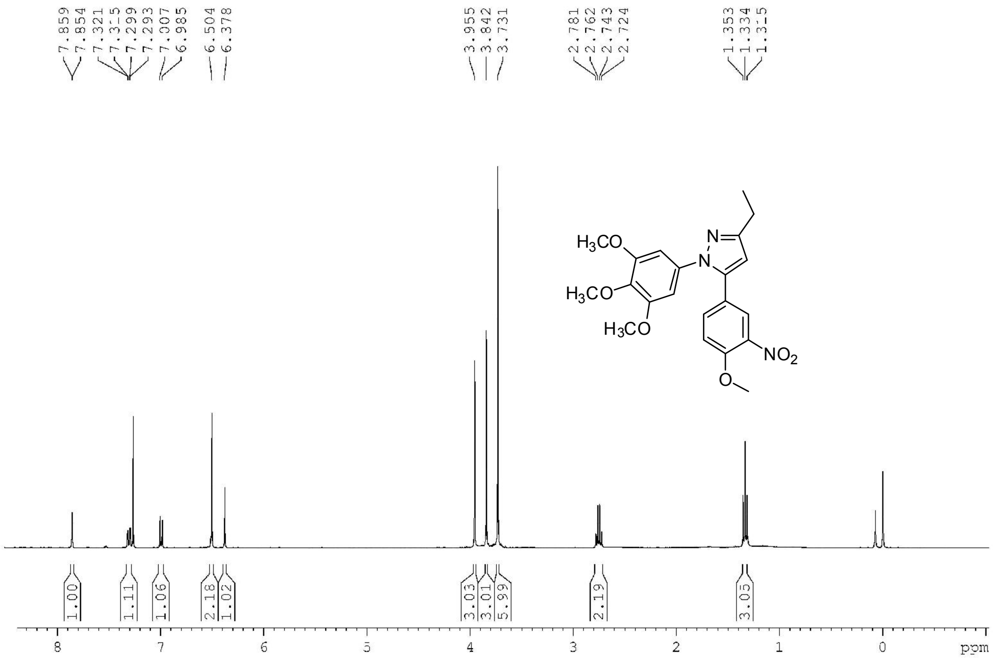

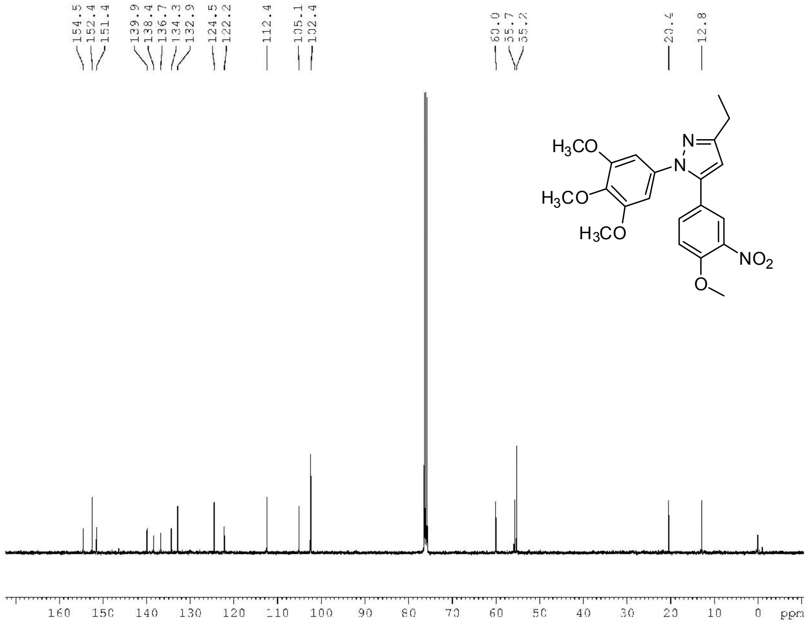

3-Propyl-5-(4-methoxy-3-nitrophenyl)-1-(3,4,5-trimethoxyphenyl)-1*H*-pyrazole(7q)

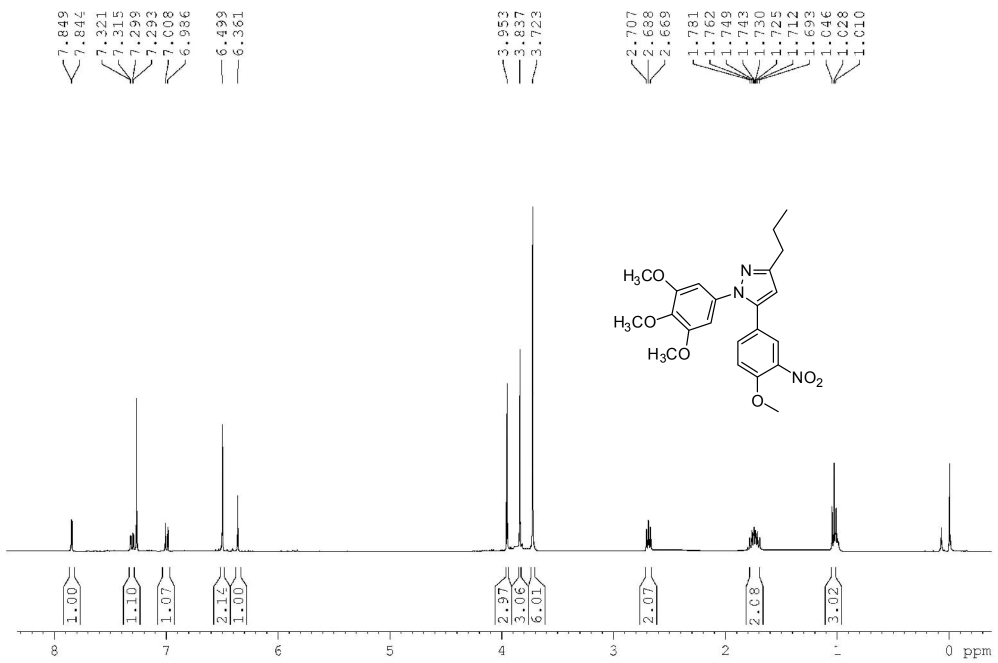

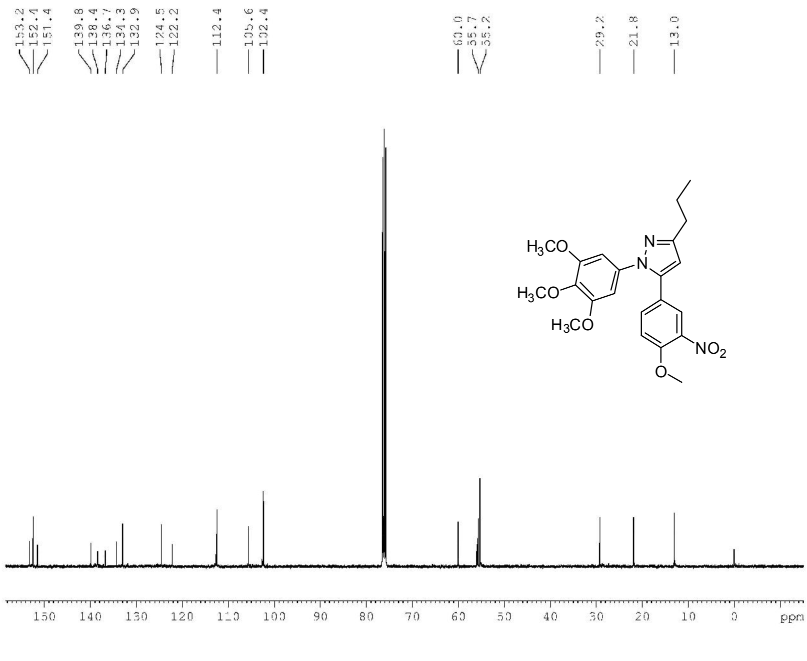

3-Ethyl-5-(3-amino-4-methoxyphenyl)-1-(3,4,5-trimethoxyphenyl)-1*H*-pyrazole (7r)

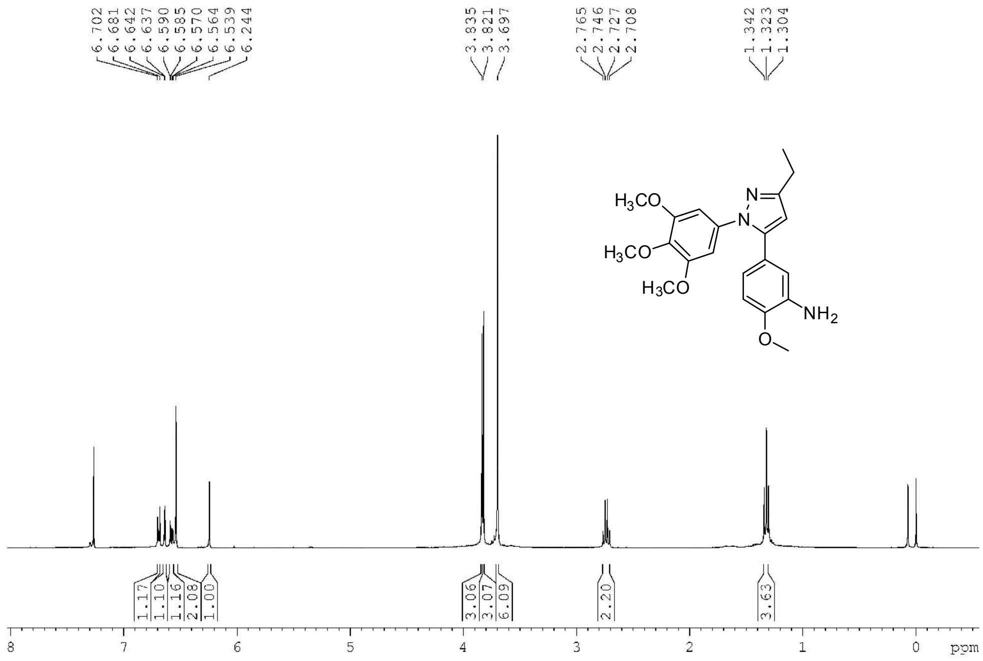

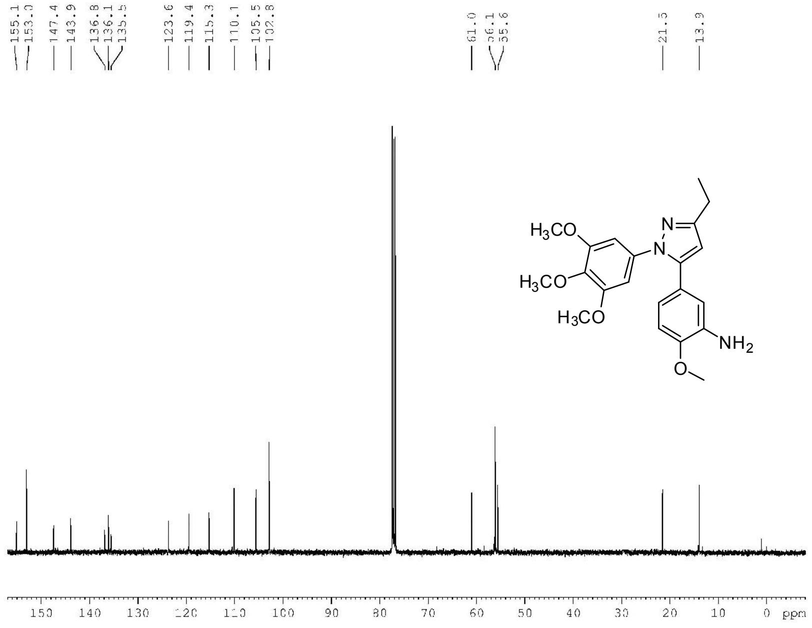

3-Propyl-5-(3-amino-4-methoxyphenyl)-1-(3,4,5-trimethoxyphenyl)-1*H*-pyrazole(7s)

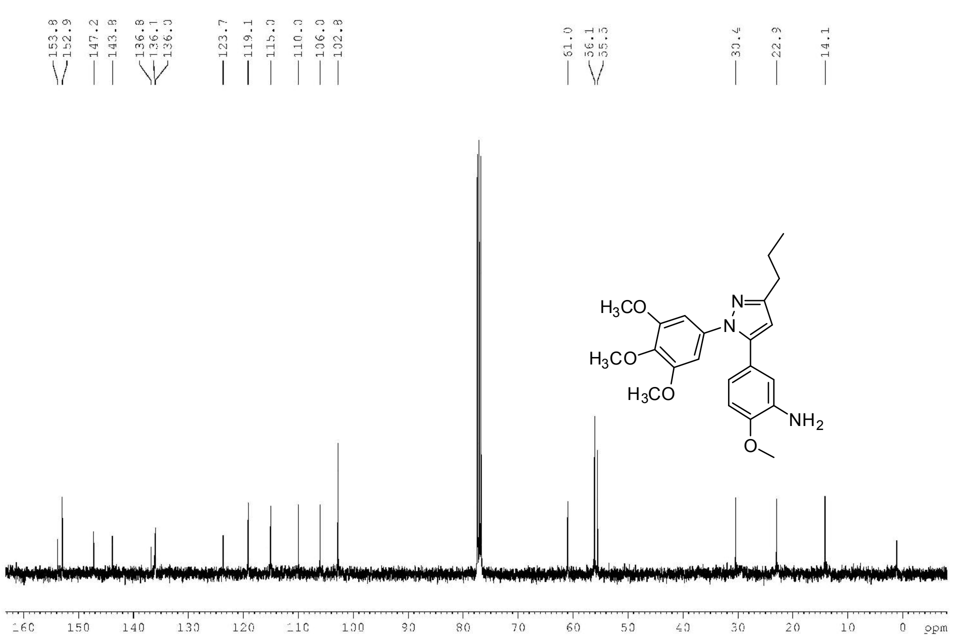

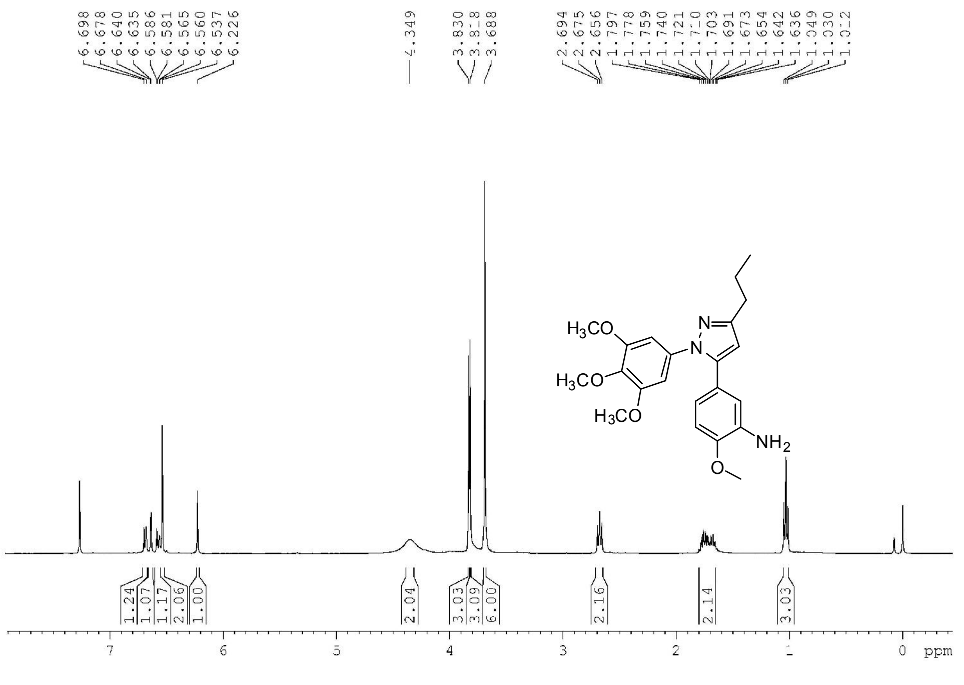

3-Methyl-1-phenyl-5-(3,4,5-trimethoxyphenyl)-1*H*-pyrazole (8a)

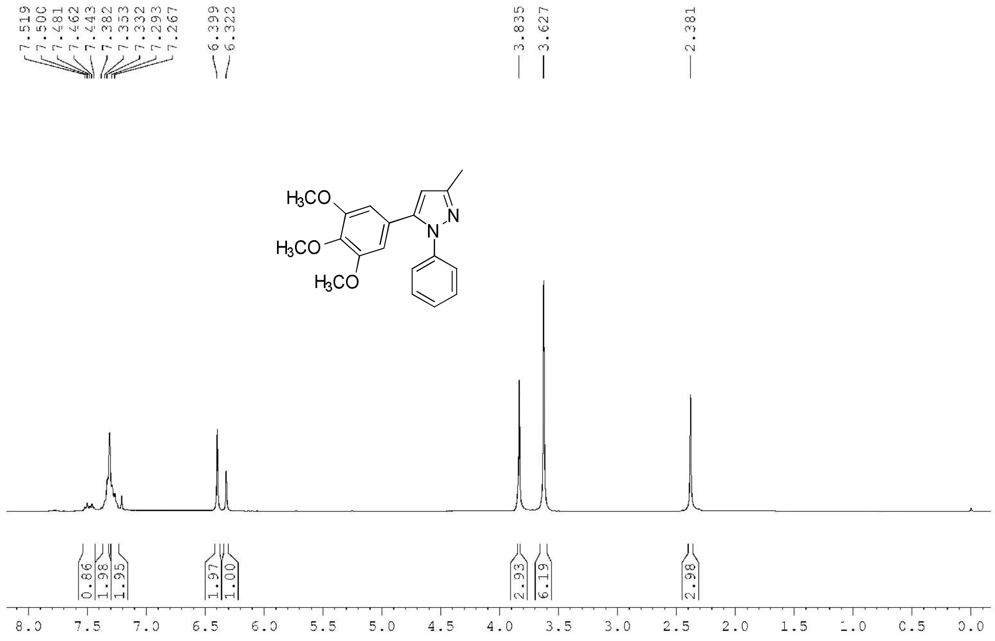

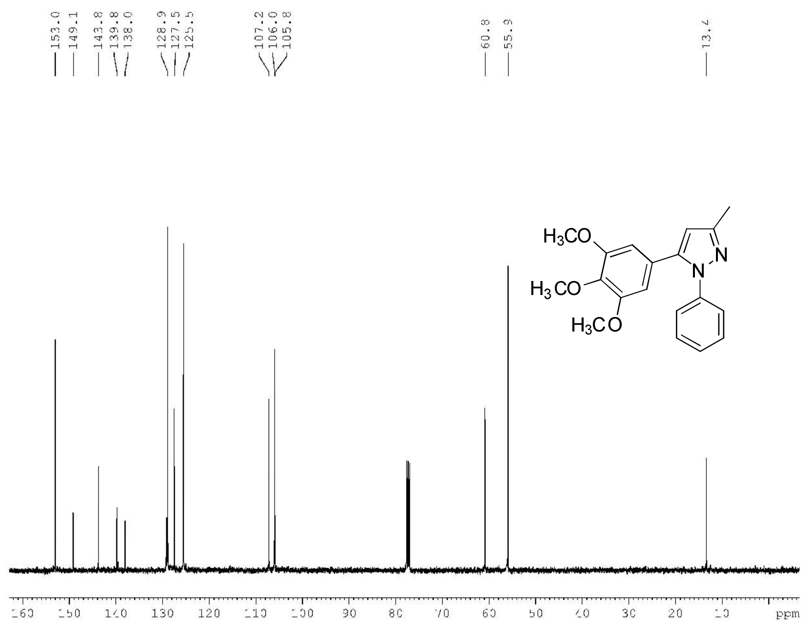

3-Methyl-1-(4-methoxyphenyl)-5-(3,4,5-trimethoxyphenyl)-1*H*-pyrazole (8b)

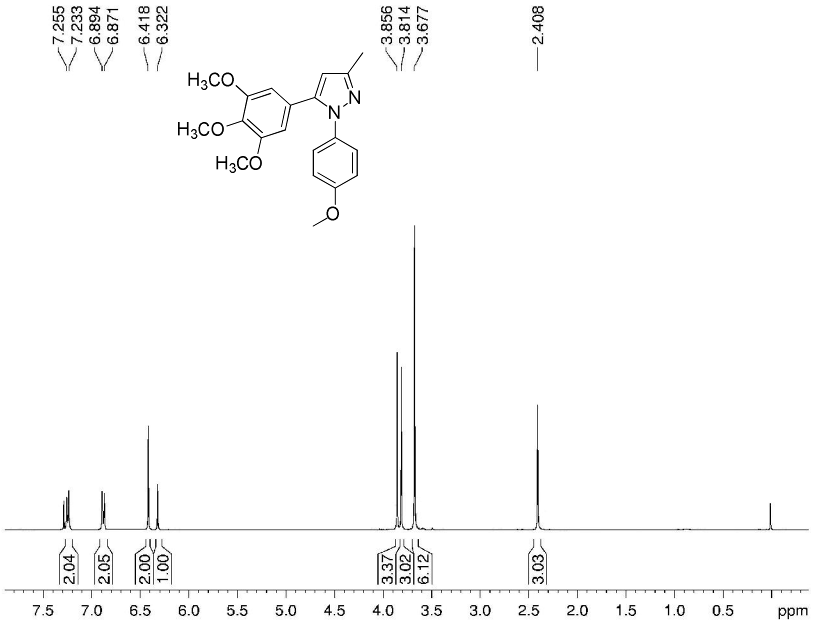

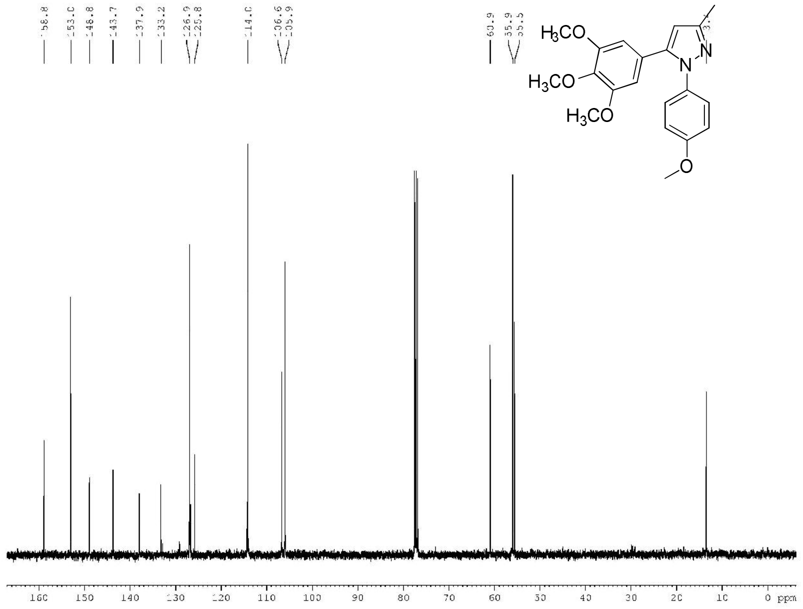

3-Methyl-1-(4-methylphenyl)-5-(3,4,5-trimethoxyphenyl)-1*H*-pyrazole (8c)

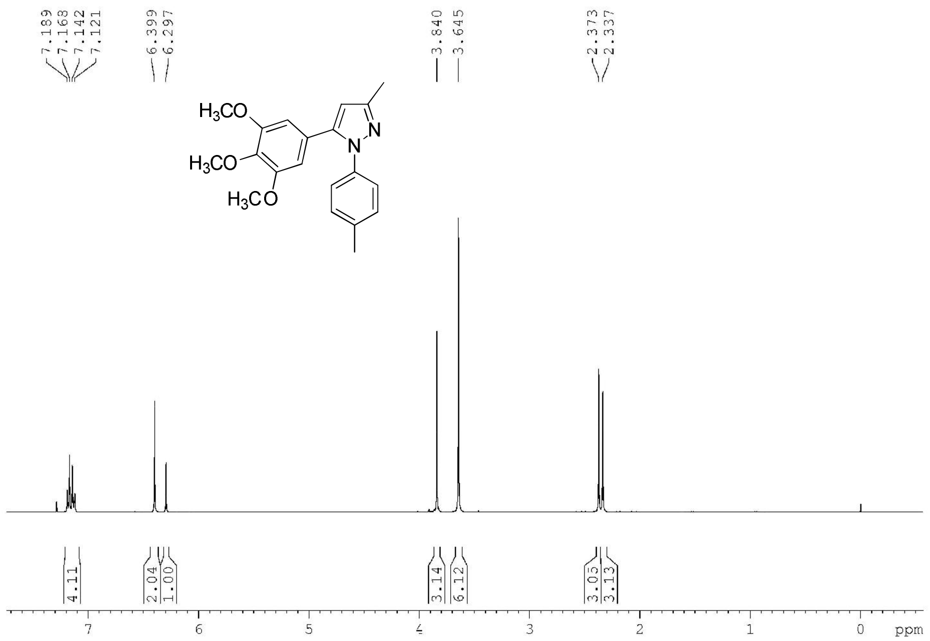

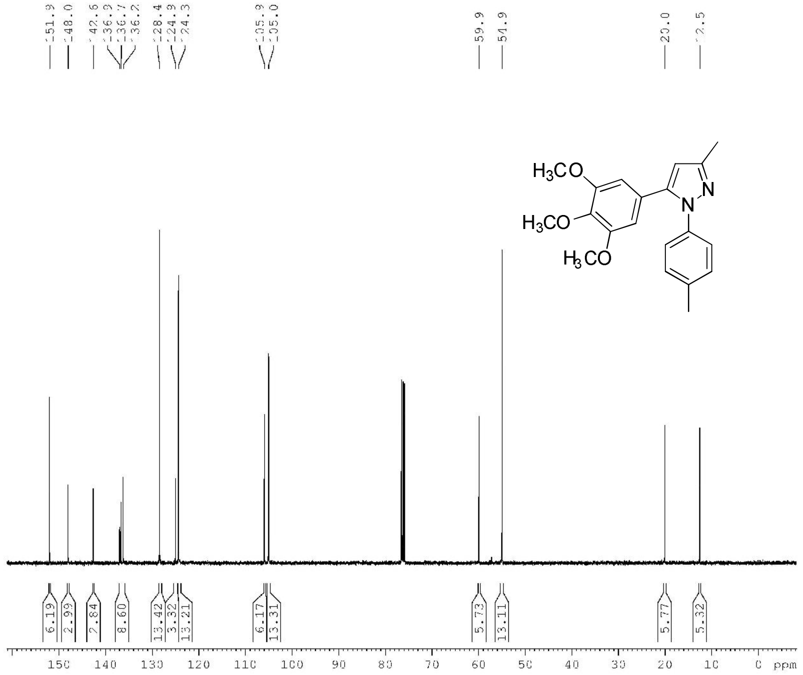

3-Methyl-1-(2-methyl-5-nitrophenyl)-5-(3,4,5-trimethoxyphenyl)-1*H*-pyrazole (8d)

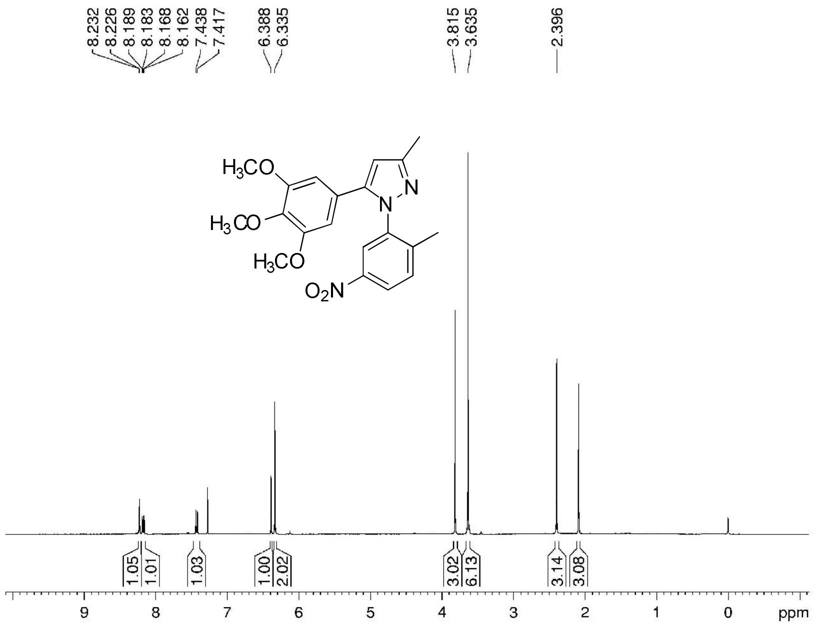

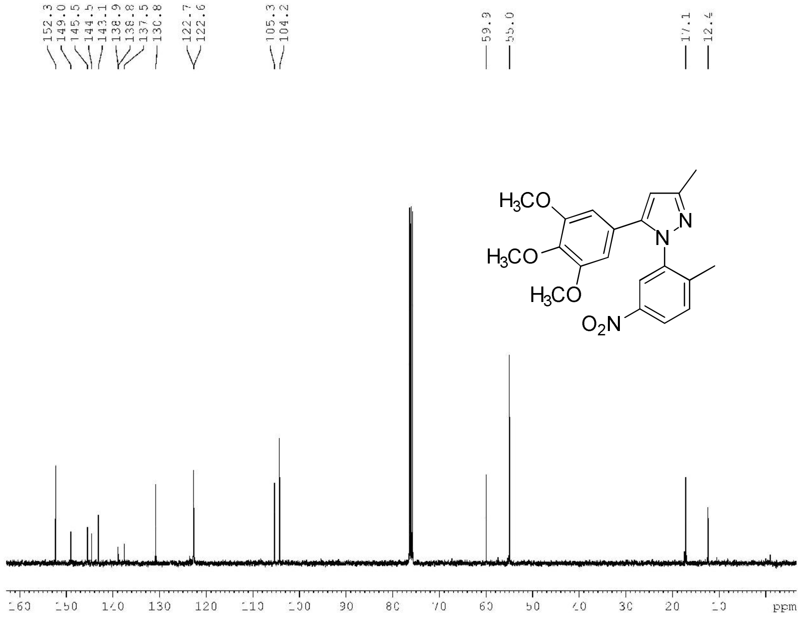

3-Methyl-1-(5-amino-2-methylphenyl)-5-(3,4,5-trimethoxyphenyl)-1*H*-pyrazole (8e)

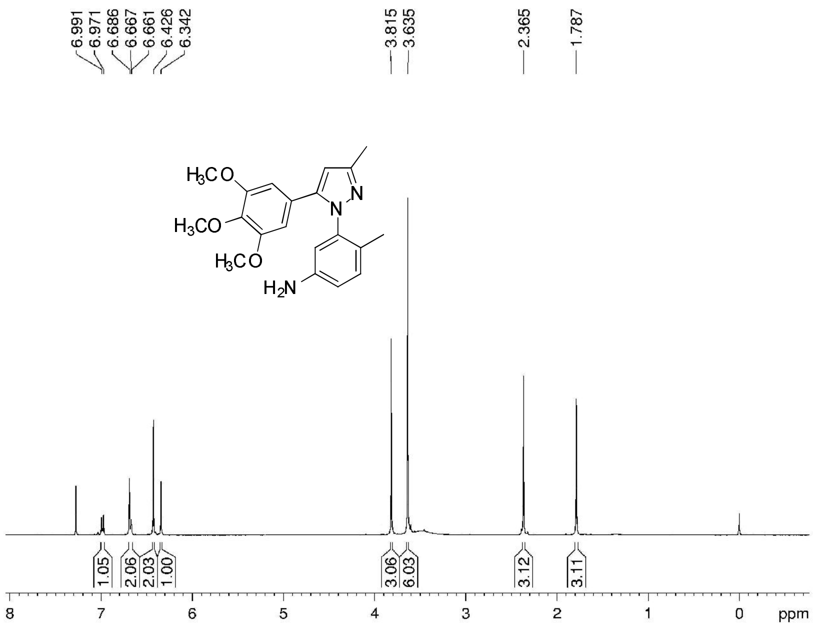

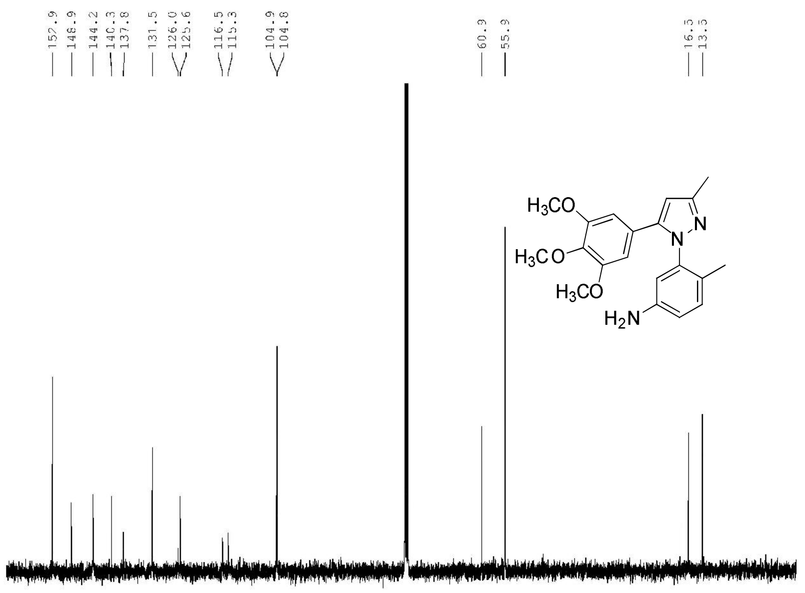

3-Methyl-1-(4-methoxy-3-nitrophenyl)-5-(3,4,5-trimethoxyphenyl)-1*H*-pyrazole (8f)

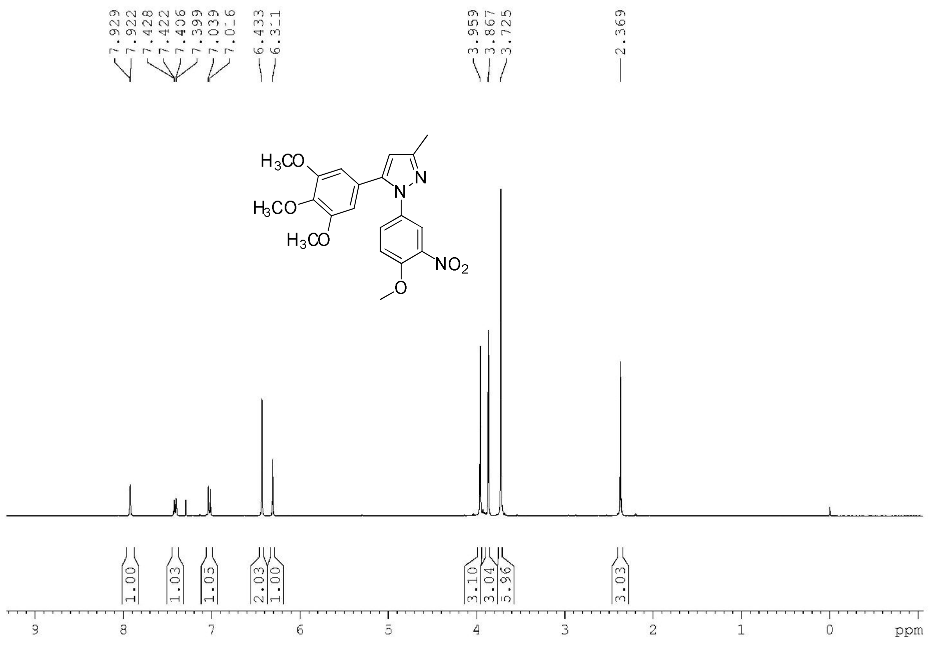

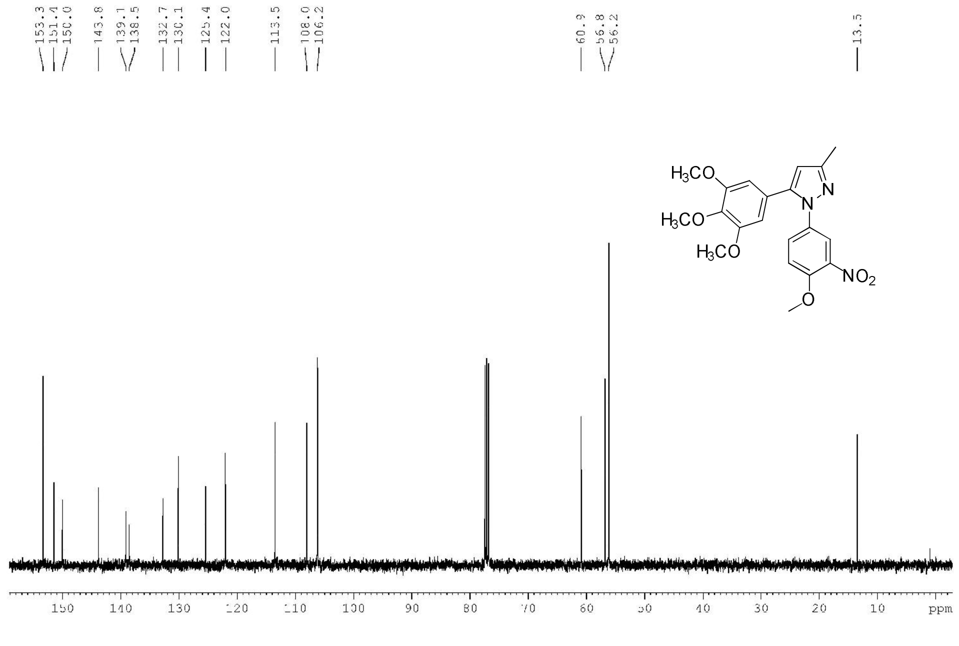

3-Methyl-1-(3-amino-4-methoxyphenyl)-5-(3,4,5-trimethoxyphenyl)-1*H*-pyrazole (8g)

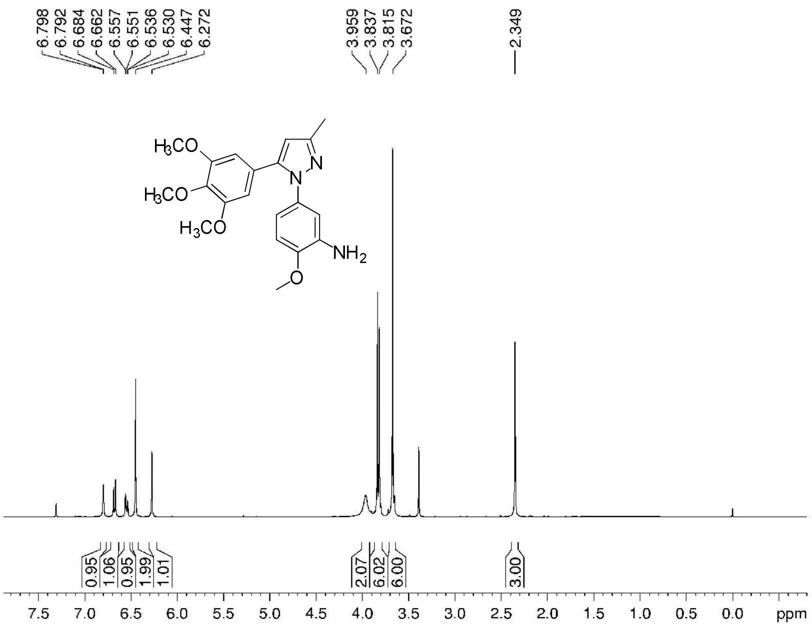

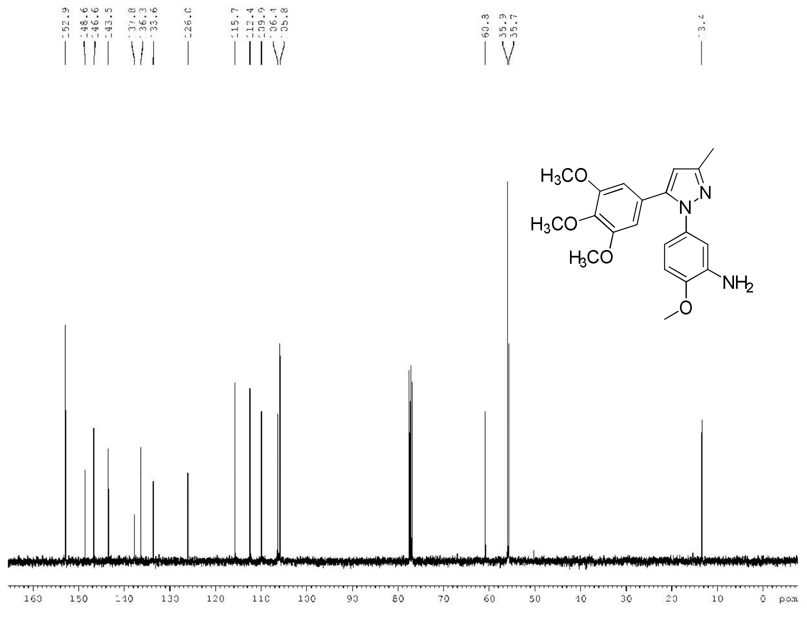

3-Methyl-1-(3-allyloxy-4-methoxyphenyl)-5-(3,4,5-trimethoxy-phenyl)-1*H*-pyrazole (8h)

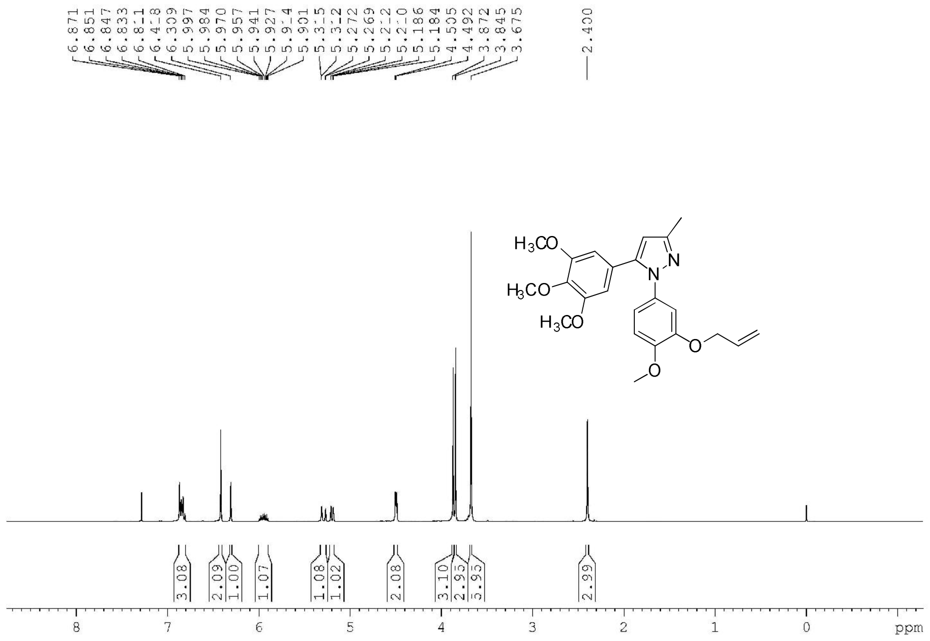

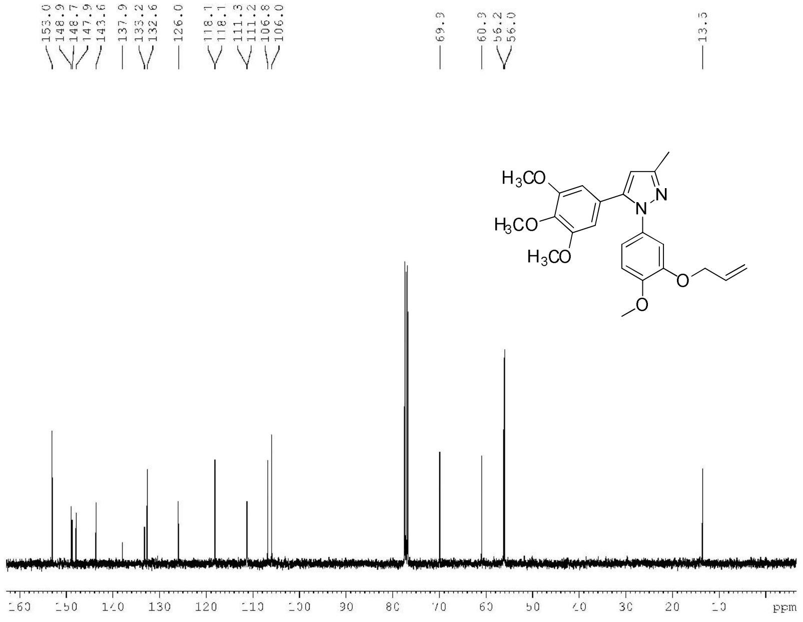

3-Methyl-1-(3-hydroxy-4-methoxyphenyl)-5-(3,4,5-trimethoxyphenyl)-1*H*-pyrazole (8i)

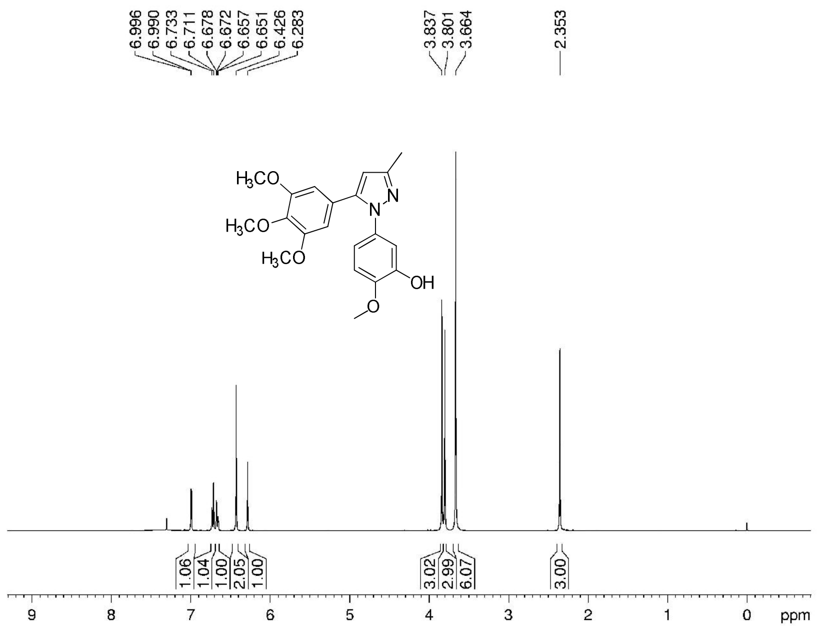

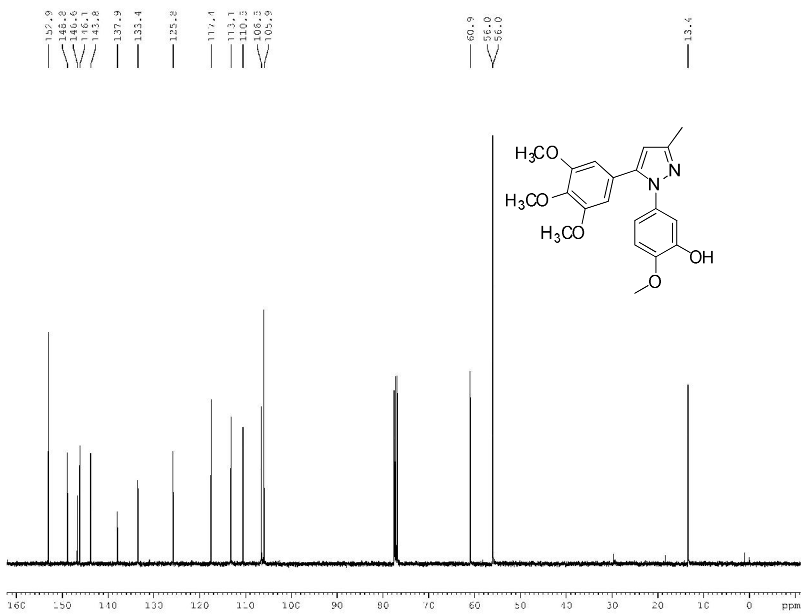

Intermediates

1-(3,4,5-Trimethoxyphenyl)butane-1,3-dione(16)

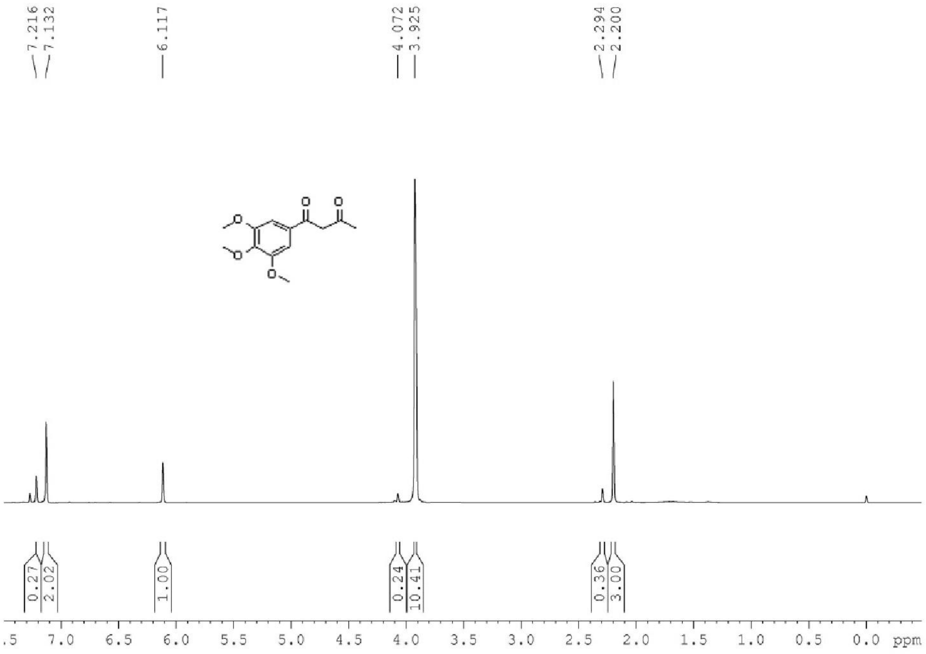

1-Phenylbutane-1,3-dione(14a)

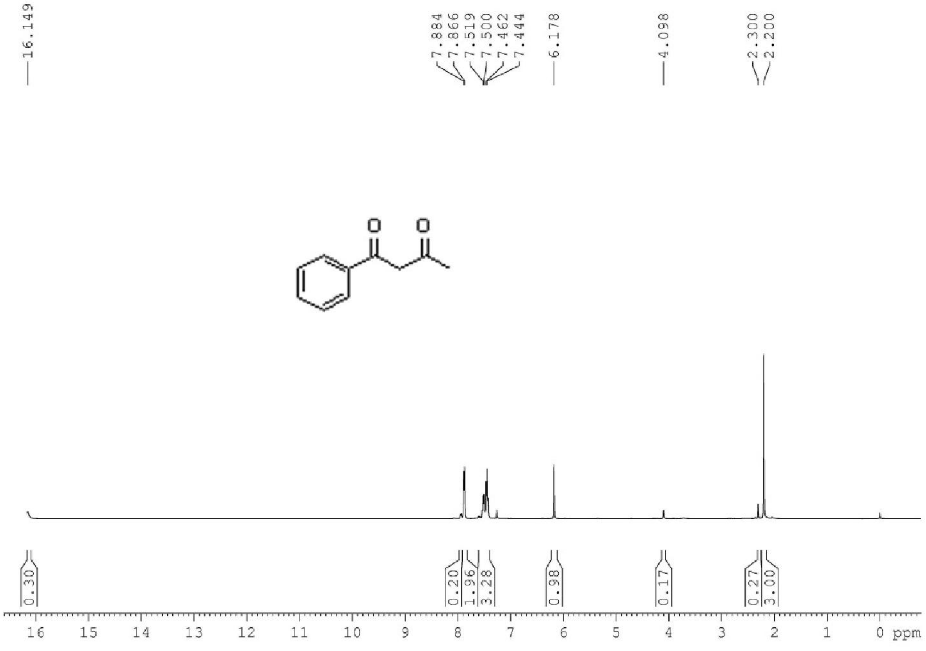

1-(4-Methoxyphenyl)butane-1,3-dione(14b)

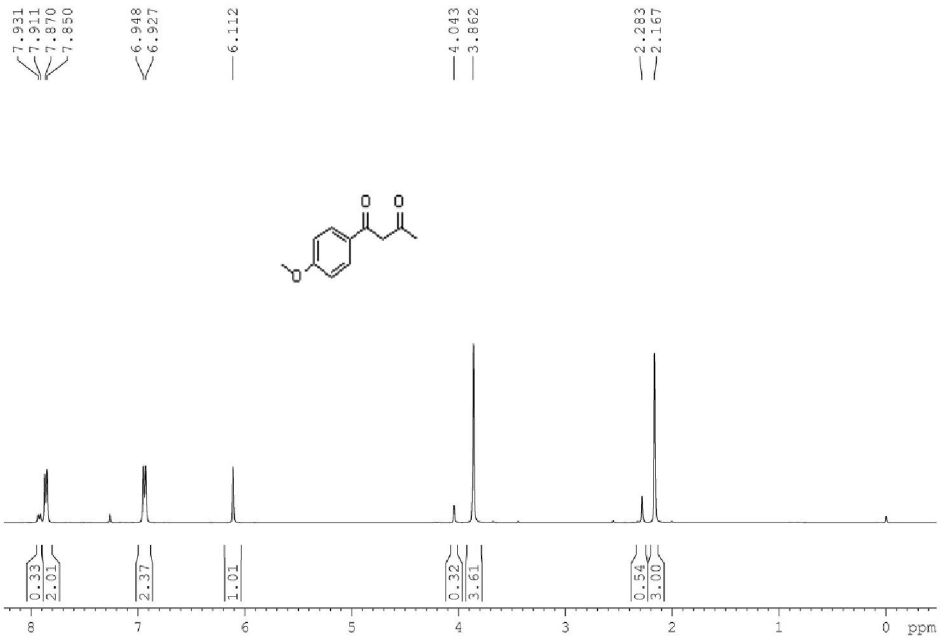

1-P-tolylbutane-1,3-dione(14c)

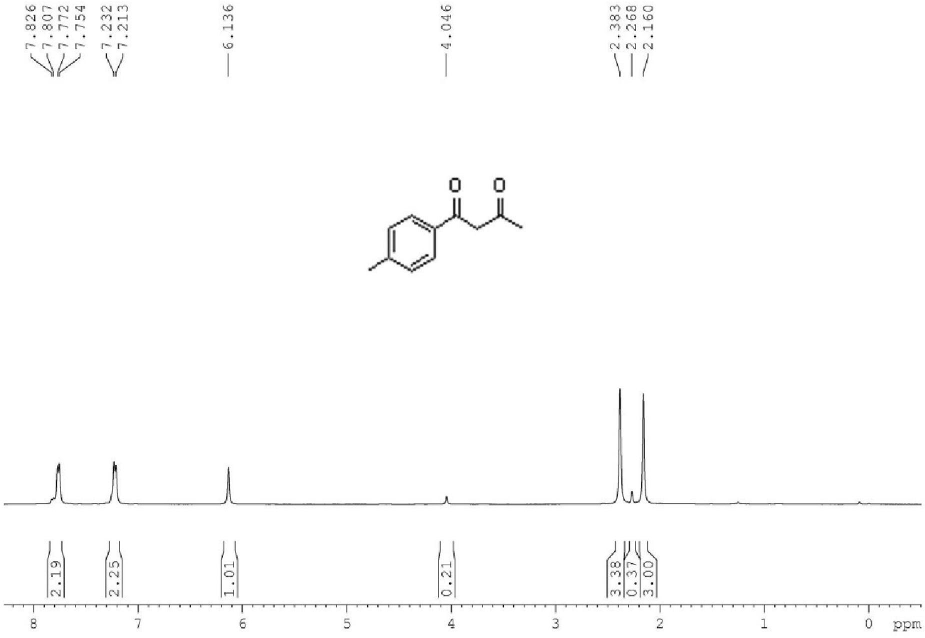

1-(4-Fluorophenyl)butane-1,3-dione(14d)

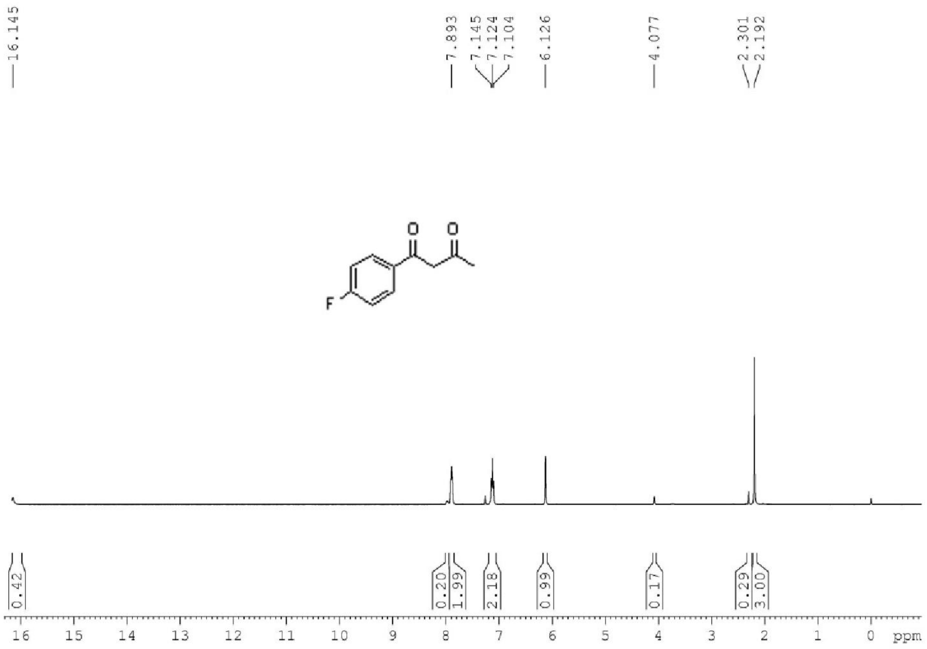

1-(3-Fluoro-4-methoxyphenyl)butane-1,3-dione(14e)

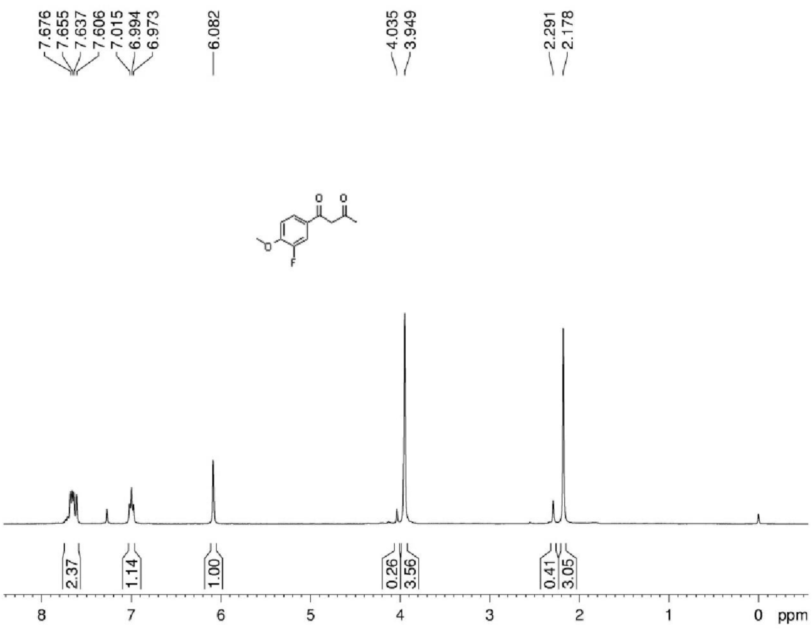

1-(4-(Allyloxy)-3-methoxyphenyl)butane-1,3-dione(14f)

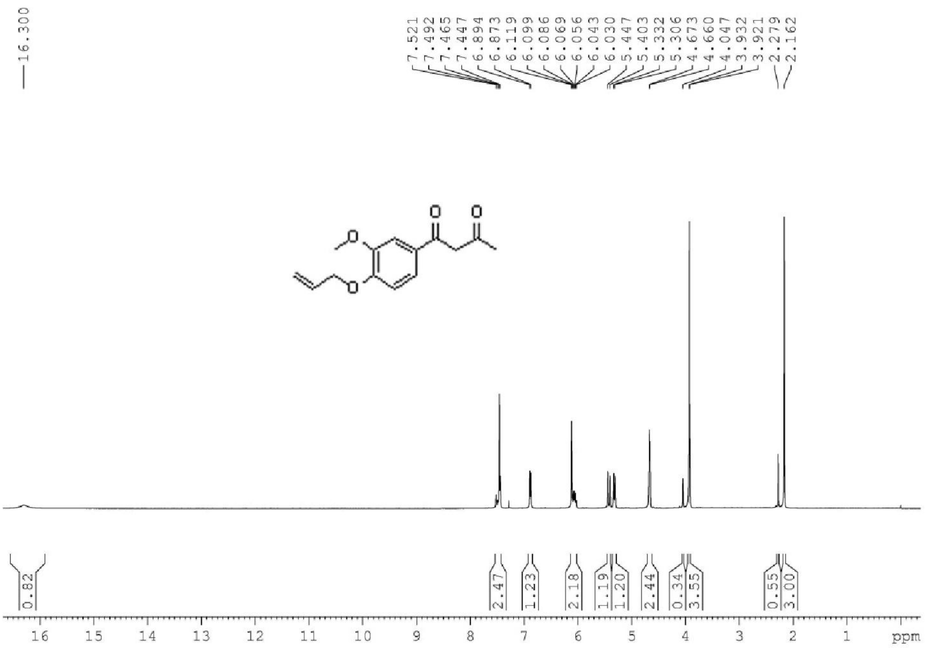

1-(3-(Allyloxy)-4-methoxyphenyl)butane-1,3-dione(14g)

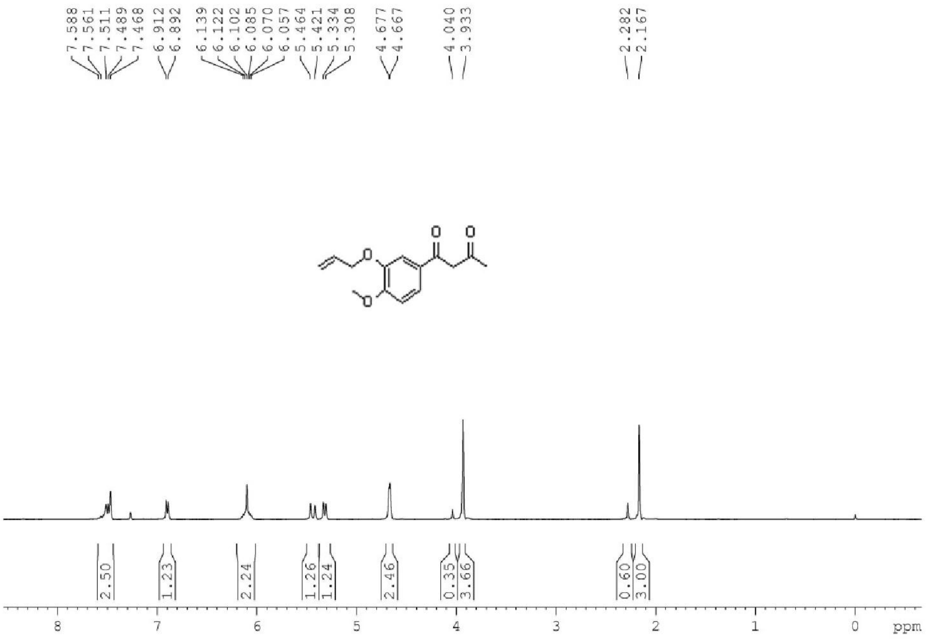

1-(4-Methoxy-3-nitrophenyl)butane-1,3-dione(14j)

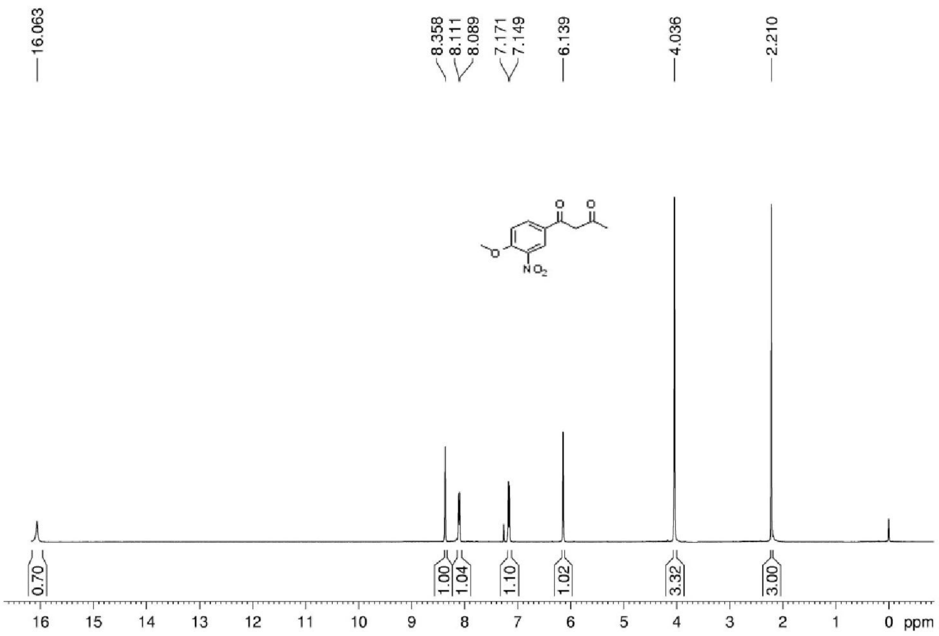

Supplement: S1 File — Supporting Information include the NMR (1H and 13C) spectra of the synthesized pyrazole derivatives and representative intermediates. (PDF) [file pone.0128710.s001.pdf]
